# Unveiling the Phosphine-Mediated *N*-Transfer from Azide to Isocyanide en Route to Carbodiimides and 4-Imino-1,3,2-diazaphosphetidines

**DOI:** 10.1021/acs.orglett.4c03902

**Published:** 2024-12-21

**Authors:** Aurelia Pastor, Carmen Lopez-Leonardo, Guillermo Cutillas-Font, Alberto Martinez-Cuezva, Marta Marin-Luna, Jose-Antonio Garcia-Lopez, Isabel Saura-Llamas, Mateo Alajarin

**Affiliations:** † Departamento de Química Orgánica, Facultad de Química, Regional Campus of International Excellence “Campus Mare Nostrum”, Universidad de Murcia, E-30100Murcia, Spain; ‡ Departamento de Química Inorgánica, Facultad de Química, Regional Campus of International Excellence “Campus Mare Nostrum”, Universidad de Murcia, E-30100Murcia, Spain

## Abstract

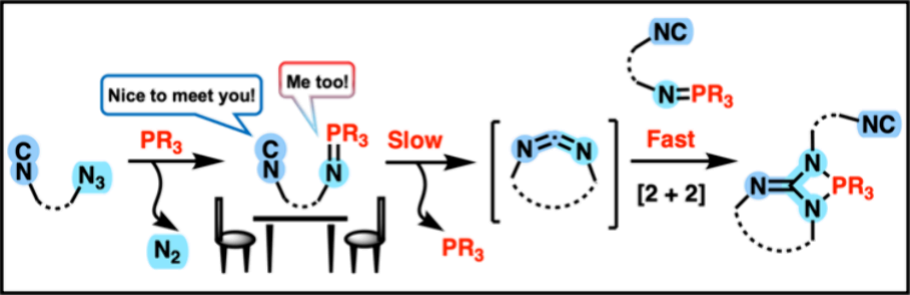

Intramolecular reactions between isocyano and iminophosphorane functions yield species containing an embedded 1,3,2-diazaphosphetidine ring, as result of the [2 + 2] cycloaddition of the primary reactive product, the cyclic carbodiimide, with a second unit of reactant. DFT studies reveal a first rate-determining step entailing a [2 + 1] cycloaddition involving the isocyanide carbon atom and the P=N double bond, with the further intervention of a dipolar precursor of the intermediate carbodiimide. The 1,3,2-diazaphosphetidine ring of the final products is shown to be hydrolytically and thermally labile.

Isocyanides, with its peculiar divalent carbon atom, have captured the attention of chemists since ancient times.^1^ This carbon enables its chameleonic reactivity, the simultaneous α-addition of a strong electrophile and a nucleophile.^2^ The best-known chemical behavior of isocyanides relies in its participation in multicomponent reactions.^3^ Some of us have recently reported the synthesis of a series of functionalized cyanides and isocyanides bearing an azido group at a six-bond distance of the (iso)cyano group, compounds **1a** and **2a**–**d** (Scheme 1a), and studied their cyclization by the interaction between both functions.^4^ Whereas the thermally activated reaction of the azido-cyanide **1a** occurred as expected, yielding a new fused tetrazole ring, compound **3a**, the analogous isocyanides **2a**–**d** only cyclized under the activation of the azide anion as a catalyst, unexpectedly giving rise to the fused cyanamides **4a**–**d**. With compounds **2** in hand, we next studied the reaction of isocyanides with a nearby iminophosphorane function, a process not previously disclosed neither in intramolecular nor intermolecular versions (Scheme 1b).^5^ Herein we reveal the results of such a study showing a novel reactivity mode of the isocyanide function, inserting into the N=P double bond of iminophosphoranes in a formal [2 + 1] cycloaddition. Although the putative products, CNP three-membered rings, are acquainted by computational methods as shown below, these species do not survive under the reaction conditions, leading to heterotetracycles containing a CN_2_P four-membered ring as the final products of such processes.

**Scheme 1:**
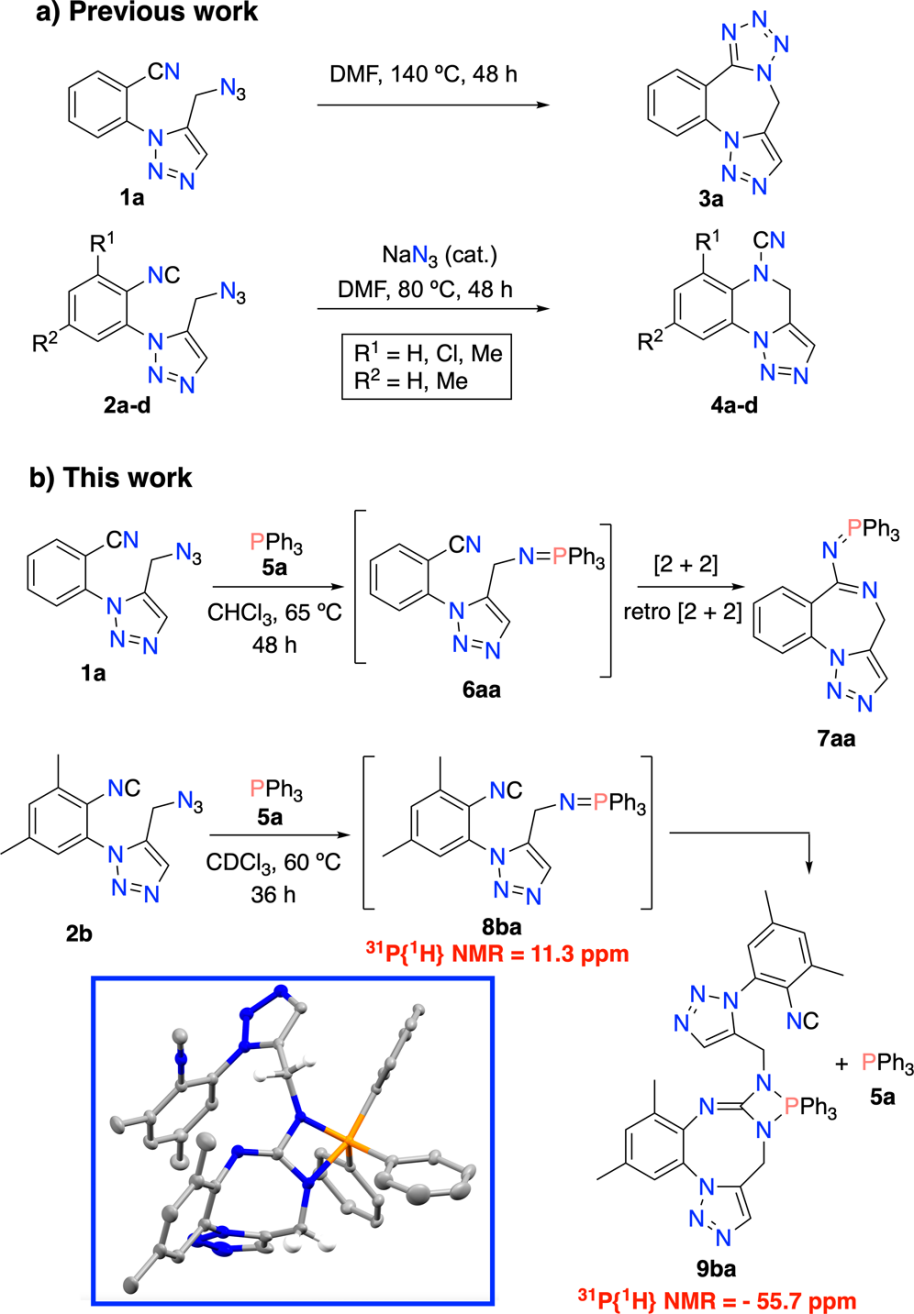
(a) Cyclization of (Iso)cyano Compounds 1a and 2a–d Previously Reported by Some of Us;^4^ (b) Reaction of (Iso)cyano Compounds 1a and 2b with Triphenylphosphine

We first checked the reaction between the *cyanide* and iminophosphorane functional groups, a known process leading to the (*N*-imidoyl)iminophosphoranes through a [2 + 2]-retro [2 + 2] cycloaddition protocol (Scheme 1b).^6^ As desired, in situ cyano-iminophosphorane **6aa** prepared from the reaction of **1a** with triphenylphosphine (**5a**), was converted by heating at 65 °C in CHCl_3_ solution under N_2_ into the new imidoyl-phosphazene **7aa** in moderate yield (60%) (Figure S1, Supporting Information). By contrast, the thermal activation at 60 °C of *isocyano*-iminophosphorane **8ba** prepared from **2b** unexpectedly resulted in the recovery of PPh_3_ and the formation of **9ba**, a species containing a fused 1,3,2-diazaphosphetidine ring, a scarcely reported organophosphorus small cycle (Scheme 1b). Next, we monitored the reaction of an equimolecular mixture of azidoisocyanide **2b** and PPh_3_ in dry CDCl_3_ at −40 °C by ^1^H and ^31^P NMR spectroscopy (Figures S2 and S3, Supporting Information). Reaching −10 °C, we detected the first reaction product, the PN_3_ adduct^7^ (^31^P signal at +19.4 ppm), which rapidly decays by increasing the temperature to 25 °C, converting into the expected iminophosphorane (λ^5^-phosphazene) by N_2_ extrusion (^31^P at +11.3 ppm, C*H*_2_^1^H at 4.36 ppm, ^3^*J*_HP_ = 17.9 Hz). By increasing the temperature to 60 °C, we observed the slow decrease with time of the iminophosphorane signal in the ^31^P spectra and the progressive appearance of two others, one at −55.7 ppm,^8^ which we initially attributed to a three-membered CNP ring^9^ but finally identified as **9ba**, and a second one at −7.8 ppm corresponding to PPh_3_. After 36 h at 60 °C, only these two latter signals remained in the ^31^P NMR spectrum of the final reaction mixture in a 1:1 ratio. The identity of **9ba** was corroborated by an X-ray determination (Scheme 1b).

To verify the general scope of the reaction, we next checked the reactions of a range of azido isocyanides **2b**–**d** with phosphines **5a**–**e** (Table 1). We first studied these processes under the reaction conditions used in our successful preparation of **9ba**. In this way, we could prepare only a new additional sample of our target compounds (**9be**, entry 4). However, other reactions yielded complex mixtures of products, most probably due to further degradation of the expected products (see below). Taking this into account, we next carried out the reactions into the NMR probe for controlling the optimal reaction time of each process by ^1^H and ^31^P spectroscopy. In this manner, we successfully prepared up to 12 examples of compounds **9** in yields ranging 40–95% (Table 1). The notable influence of the substituents at the phenyl ring bearing the isocyano group in the speed of these reactions is shown in this table (reaction times ranging 1–36 h). Note that the processes with the *o*-Cl substrate (**2d**) were rather rapid (entries 8–12), whereas *o-* and *p-*CH_3_ groups slow these reactions (entries 1–4).

**Table 1:**
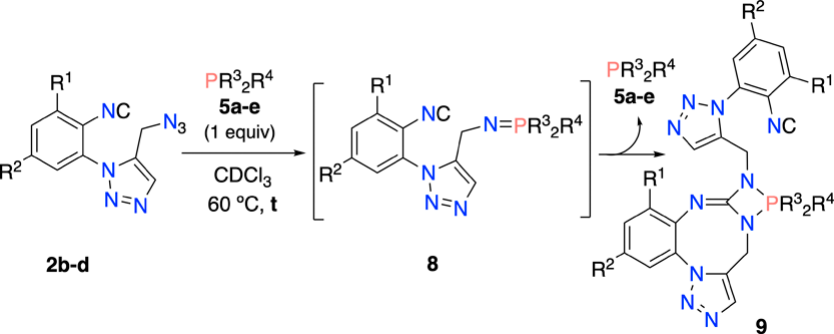
Scope of the Reaction of Azidoisocyanides 2b–d with Phosphines 5a–e To Give Compounds 9 (DAP)^a^

**Entry**	**DAP (9)**	**R**^**1**^	**R**^**2**^	**R**^**3**^	**R**^**4**^	**Yield (9) (reaction time)**
1	**9ba**	Me	Me	C_6_H_5_	C_6_H_5_	94% **(36 h)**
2	**9bb**	Me	Me	*m*-Me-C_6_H_4_	*m*-Me-C_6_H_4_	74% (32 h)
3	**9bd**	Me	Me	3,5-Me_2_-C_6_H_3_	3,5-Me_2_-C_6_H_3_	78% (28 h)
4	**9be**	Me	Me	C_6_H_5_	*p*-Me-C_6_H_4_	86% (36 h)
5	**9ca**	Me	H	C_6_H_5_	C_6_H_5_	80% (20 h)
6	**9cb**	Me	H	*m*-Me-C_6_H_4_	*m*-Me-C_6_H_4_	65% (12 h)
7	**9cd**	Me	H	3,5-Me_2_-C_6_H_3_	3,5-Me_2_-C_6_H_3_	93% (15 h)
8	**9da**	Cl	Me	C_6_H_5_	C_6_H_5_	85% (1.75 h)
9	**9db**	Cl	Me	*m*-Me-C_6_H_4_	*m*-Me-C_6_H_4_	72% (1.5 h)
10	**9dc**	Cl	Me	*p*-Cl-C_6_H_4_	*p*-Cl-C_6_H_4_	40% (4 h)
11	**9dd**	Cl	Me	3,5-Me_2_-C_6_H_3_	3,5-Me_2_-C_6_H_3_	95% (1 h)
12	**9de**	Cl	Me	C_6_H_5_	*p*-Me-C_6_H_4_	89% **(1.75 h)**

a Reaction times (*t*) are given in each entry.

At this point, we should remark that several unsuccessful experiments were run with other phosphines, for instance P(C_6_H_4_-Me-*o*)_3_, not reacting with the azido group due to steric reasons, and PBu^*n*^_3_ yielding a stable PN_3_ phosphazide not converting into the iminophosphorane under the general reaction conditions. In other cases, the expected products **9** were detected by ^1^H (four characteristic multiplets in the CH_2_ region) and ^31^P NMR but apparently degraded very rapidly, precluding their isolation in pure form (Table S1, Supporting Information). Such decomposition processes resulted in very complex aliphatic regions at the ^1^H NMR spectra (appearing signals attributable to at least two new species, besides those of **9**) and in the rising of the signals corresponding to the respective phosphine and its oxide in their ^31^P NMR spectra, both increasing at the expense of the resonance attributed to **9**. These were the cases of iminophosphoranes derived from electron-rich phosphines such as P(C_6_H_4_-OMe-*p*)_3_, P(C_6_H_4_-Me-*p*)_3_ and PPh_2_Me, resulting in rapid reactions and subsequent degradations, but also of some attempts with less nucleophilic phosphines as P(C_6_H_4_-Cl-*p*)_3_ reacting slowly but showing rapid degradation of the putative species **9**. As summarized in Table 1, triphenylphosphine and other triaryl partners with similar electronic characteristics, such as P(C_6_H_4_-Me-*m*)_3_, P(C_6_H_3_-Me_2_-3,5)_3_ and PPh_2_(C_6_H_4_-Me-*p*), gave the best results in terms of yield and stability of the reaction products **9**.

Species with the key 4-imino-1,3,2-diazaphosphetidine fragment, present in compounds **9**, have been scarcely documented (Scheme 2). They were first proposed as nonisolable reactive intermediates by R. Huisgen^10^ and later on by Boedeker,^11^ in a reaction between a carbodiimide and an iminophosphorane leading to another pair of similar species via a [2 + 2]-retro[2 + 2] cycloaddition sequence.

**Scheme 2:**
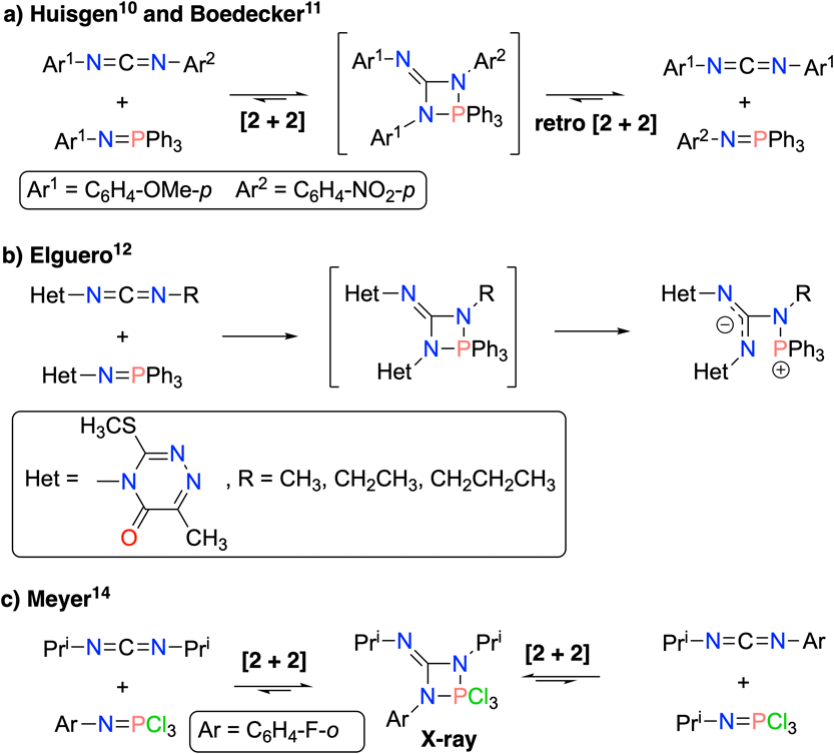
Previous Examples of Reactions between Carbodiimides and Iminophosphoranes Affording 4-Imino-1,3,2-diazaphosphetidines as Either Proposed Intermediates or Isolated Products

Some of us also proposed 4-imino-1,3,2-diazaphosphetidines as necessary, nonisolable intermediates for explaining the formation of phosphonium betaines by reaction of heterocyclic *N*-iminophosphoranes with some alkyl isocyanates.^12,13^ Finally, Meyer could isolate the first example of these small rings by reaction of diisopropylcarbodiimide with *o*-F–C_6_H_4_–N=PCl_3_ and determined its crystal structure by X-ray diffraction.^14^

Consistent with those precedents, the more reasonable explanation for the formation of compounds **9** is the reaction of iminophosphoranes **8** with the cyclic carbodiimide intermediates **12** in a regioselective [2 + 2] cycloaddition manner, involving the most nucleophilic nitrogen atom (*N*-CH_2_) of the heterocumulene function (Figure 1, upper box). In a previous step, the key intramolecular reaction between the isocyano and R^3^_2_R^4^P=N functionalities of the starting materials should yield the cyclic carbodiimides **12** and the corresponding phosphine.^15^ This step should occur at such low rate that the reactive carbodiimides **12** always find, in the reaction medium, a high molar ratio of its precursory isocyano-iminophosphorane, thus leading to the fused 4-imino-1,3,2-diazaphosphetidines **9** through a formal intermolecular [2 + 2] cycloaddition (Figure 1, upper box). [2 + 2] Cycloaddition reactions of carbodiimides are well-known and common processes,^16^ especially when confronted with heterocumulenes such as (thio)ketenes, iso(thio, seleno)cyanates and ketenimines. Carbodiimides have been also shown to undergo [2 + 2] cyclodimerizations yielding 1,3-diazetidine-2,4-diimines.^17^

**Figure 1:**
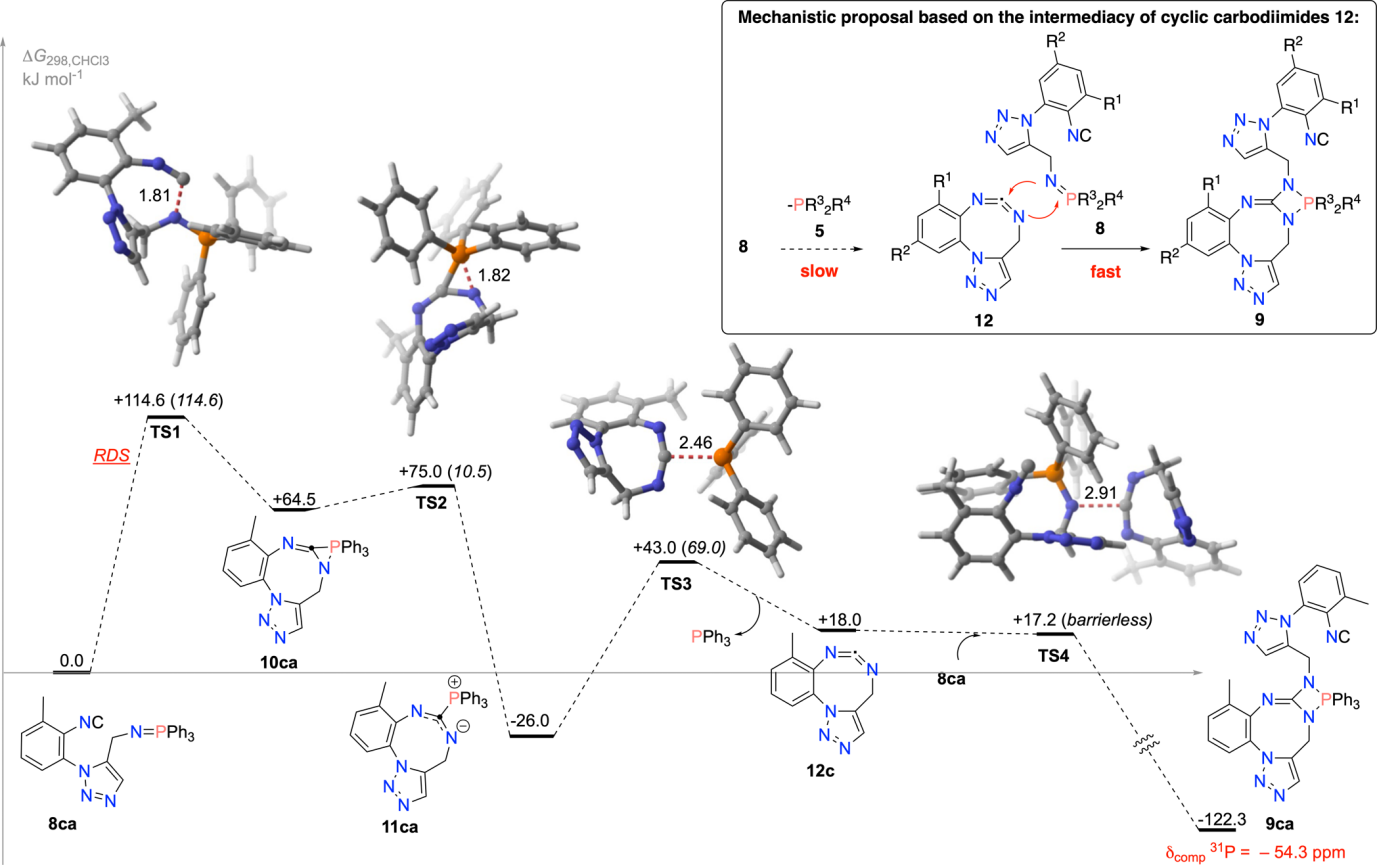
Computed mechanism for the conversion of isocyano-iminophosphorane **8ca** into the fused 4-imino-1,3,2-diazaphosphetidine **9ca**. Energy barriers (kJ mol ^–1^) are shown in parentheses. Upper box: Mechanistic proposal based on previous knowledge.

To explore the above mechanistic proposal, we carried out a computational DFT study of the reaction path leading from the isocyano-iminophosphorane **8ca** to the cyclic carbodiimide and then to the [2 + 2] cycloadduct **9ca** (Figure 1). As shown, the reaction between the isocyano- and iminophosphorane functions is rate-determining (RDS), with an energy barrier of 114.6 kJ mol^–1^.

To reach its transition state **TS1**, the nucleophilic N atom of the N=PPh_3_ fragment approaches the carbon atom of the isocyanide function, therefore playing an electrophilic role. The IRC analysis of **TS1** reveals its progress toward the three-membered azaphosphiridine **10ca**, thus completing the insertion of the isocyanide carbon into the N=P bond for a formal, highly asynchronous [2 + 1] cycloaddition step.^18^ Intermediate **10ca** quickly converts into stabilized *N*,*P*-betaine **11ca** overcoming an energy barrier of 10.5 kJ mol^–1^. Next, the cyclic carbodiimide **12c** is formed by extrusion of a PPh_3_ molecule through **TS3**. The final stage of the process is the nucleophilic addition of **8ca**, through the N atom of its N=PPh_3_ fragment, into the electrophilic central carbon of the cyclic carbodiimide, whereas the more nucleophilic nitrogen of the heterocumulene *N*-CH_2_ binds to the phosphorus atom of that fragment for completing a formal [2 + 2] cycloaddition, by surpassing a small energy barrier.^19^ The computed ^31^P NMR shift of **9ca** is −54.3 ppm, which is in agreement with the experimental value.

To the best of our knowledge, not even in any other formulation the isocyanide plus iminophosphorane reaction has been previously reported.^20^ Note that even for the entropically favored intramolecular cyclization of **8ca**, our calculations show a high energy barrier justifying its low rate under the experimental conditions.^21^ As our DFT results revealed that the isocyanide function behaves as the electrophile in the rate-determining step, electron-withdrawing groups at the phenyl ring supporting the isocyano group should contribute to accelerating the whole process (as is the case, see entries 8–12 of Table 1).^22^ The computed rate-determining insertion of the isocyanide carbon into the N=P double bond adds to the rarely reported [2 + 1] cycloaddition reactions of isocyanides.^23^ In its general formulation, the isocyanide plus iminophosphorane reaction could be also labeled as the cross-coupling of two 1,1-dipolar synthons,^24^ residing one at the isocyanide carbon and the second at the iminophosphorane nitrogen, yielding a new C=N double bond.^25^

Finally, to further demonstrate the application potential of this reaction, we conducted a 1 mmol-scale reaction of **2b** with triphenylphosphine (**5a**). In this case, 0.331 g of 4-imino-1,3,2-diazaphosphetidine **9ba** was obtained, which corresponds to a yield of 93% (Scheme 3).

**Scheme 3:**
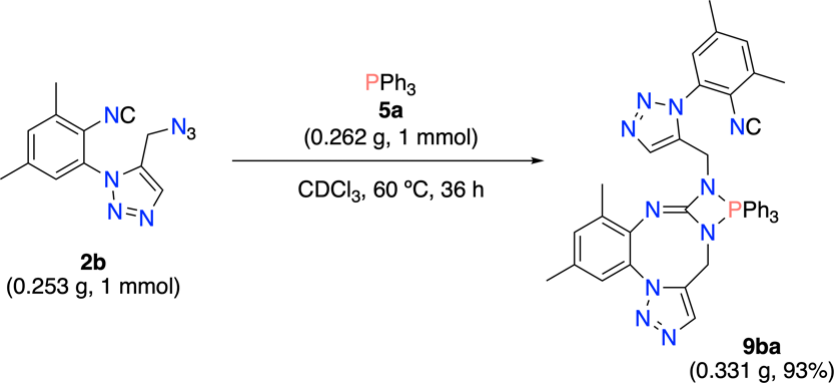
Scale-up Synthesis of 9ba

The degradation processes of compounds **9** observed in the NMR scale experiments prompted us to assay: (*i*) the hydrolysis of **9ba**, perhaps justifying the formation of OPPh_3_; and (*ii*) its degradation by heating in solution (Scheme 4). Thus, when **9ba** was submitted to a hydrolytic treatment with silica gel in CHCl_3_-ethanolic solution, it cleanly converted into the guanidine derivative **13b** and triphenylphosphine oxide in practically quantitative yield. On the other hand, prolonged heating of **9ba** in CDCl_3_ solution at 50 °C led to the formation of PPh_3_ and a non-P-containing compound.

**Scheme 4:**
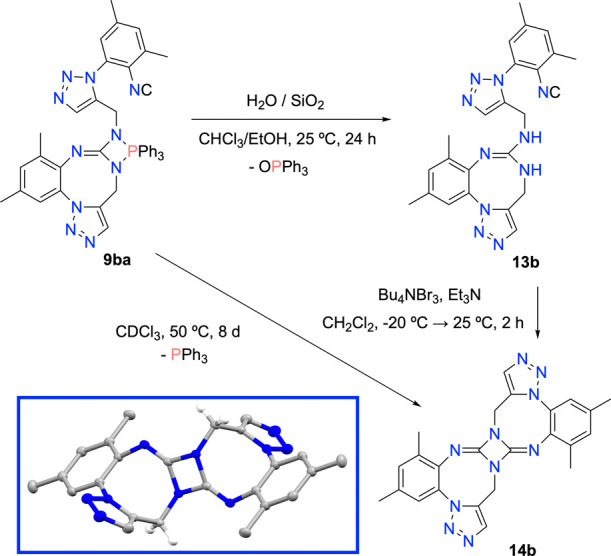
Hydrolysis and Thermal Treatment of 9ba To Give 13b and 14b Conversion of **13b** into **14b** by treatment with tetrabutylammonium tribromide.

After its separation and purification, this new species was identified as centrosymmetric, fused 2,4-diimino-1,3-diazetidine **14b** as confirmed by its X-ray structure determination. Finally, we envisioned an innovative route potentially leading also to diazetidine **14b** starting from guanidine **13b**. In fact, we successfully converted **13b** into **14b** by treatment with tetrabutylammonium tribromide in the presence of triethylamine, presumably through the respective isocyanide dibromide (−N=CBr_2_) intermediate.

In conclusion, the first examples of the reaction between the isocyanide and iminophosphorane functional groups are herein shown, occurring in an entropically favored intramolecular way. The gradual release of the phosphine fragment during the reaction was acquainted for the formation of a carbodiimide as the primary reaction product. This reaction can be regarded as a phosphine-mediated transfer of a nitrene from the azide to the isocyanide carbon atom resulting in the formation of a new N=C bond, a chemical transformation that is usually achieved by transition-metal catalysis.^26^ As soon as formed, the reactive carbodiimide couples with the starting organophosphorus compound to yield [2 + 2] cycloadducts containing the scarcely reported 1,3,2-diazaphosphetidine ring, and the reaction products were actually isolated. This stepwise mechanism is supported by DFT calculations, revealing also the [2 + 1] coupling of the two reactive fragments in the computed rate-determining first step. The global process, a tandem intramolecular–intermolecular sequence, allows the preparation of complex structures containing two units of the starting bifunctional reactant, from which isocyanoguanidines and 2,4-dimino-1,3-diazetidines are easily derived. The scope of the novel primary reaction, isocyanide plus λ^5^-phosphazene, is being currently more widely studied in our laboratories.

## Data Availability

The data underlying this study are available in the published article and its Supporting Information.

## Supporting Information Available

Synthetic procedures, experimental data, ^1^H and ^31^P NMR monitoring of the reaction of **1a** and **2b** with triphenylphosphine, ^31^P NMR data of other reactions where diazaphosphetidines were detected, crystal data and structure refinement for **9ba** (CCDC 2387940) and **14b** (CCDC 2387939), copies of the NMR spectra compounds, computational methods, computational data and Cartesian coordinates (PDF)
