# Supplementary material for: Unveiling the Phosphine-Mediated N-Transfer from Azide to Isocyanide en Route to Carbodiimides and 4-Imino-1,3,2-diazaphosphetidines
Source: Org Lett. 2024 Dec 21;27(1):73–9. doi: 10.1021/acs.orglett.4c03902 (PMC11731383; doi:10.1021/acs.orglett.4c03902)
Supplement: Supplementary file 1 — ol4c03902_si_001.pdf [file ol4c03902_si_001.pdf]

Supporting Information

Unveiling the phosphine-mediated *N*-transfer from azide to isocyanide en route to carbodiimides and 4-imino-1,3,2-diazaphosphetidines

Aurelia Pastor*,[a] Carmen Lopez-Leonardo*,[a] Guillermo Cutillas-Font,[a] Alberto Martinez-Cuezva,[a] Marta Marin-Luna,[a] Jose-Antonio Garcia-Lopez,[b] Isabel Saura-Llamas,[b] Mateo Alajarin*[a]

[a] Departamento de Química Orgánica, Facultad de Química, Regional Campus of International Excellence “Campus Mare Nostrum”, Universidad de Murcia, E-30100, Murcia, Spain

[b] Departamento de Química Inorgánica, Facultad de Química, Regional Campus of International Excellence “Campus Mare Nostrum”, Universidad de Murcia, E-30100, Murcia, Spain

E-mail: aureliap@um.es, melill@um.es, alajarin@um.es

Table of Contents page

1. General experimental section…… S3

2. Synthesis of *N*-(triphenylphosphoranylidene)-4*H*-benzo[*f*][1,2,3]triazolo[1,5-*a*][1,4]diazepin-6-amine (**7aa**)…… S4

3. General procedure for the preparation of fused diazaphosphetidines **9**…… S5

4. Synthesis of guanidine **13b**…… S11

5. Synthesis of diazetidine **14b** from guanidine **13b**…… S12

6. Synthesis of diazetidine **14b** by thermal treatment of the diazaphosphetidine **9ba**…… S13

7. $^{31}P\{^{1}H\}$ NMR monitoring of the reaction mixture of 5-azidomethyltriazole **1a** with PPh_3 (**5a**) (Figure S1)…… S14

8. ^{1}H and $^{31}P\{^{1}H\}$ NMR monitoring of the reaction mixture of 5-azidomethyltriazole **2b** with PPh_3 (**5a**) (Figures S2 and S3)…… S15

9. Other unsuccessful reactions between 5-azidomethyltriazoles **2** and phosphines **5** (Table 1)…… S17

10. X-Ray crystal data and structure refinement of **9ba** and **14b**…… S18

10.1 Crystal data and structure refinement for **9ba** (Table S2 and Figure S4)…… S18

10.2 Crystal data and structure refinement for **14b** (Table S3 and Figures S5-7)…… S19

11. Computational details…… S22

11.1 Methods…… S22

11.2 Data (Table S4)…… S22

12. NMR spectra of all new synthesized compounds…… S40

13. References…… S79

1. General experimental section.

Unless otherwise stated, all reagents were purchased from commercial sources and used without further purification. HPLC grade solvents were nitrogen saturated and were dried and deoxygenated using an Innovative Technology Inc. Pure-Solv 400 Solvent Purification System. The $CDCl_3$ (stored with 4Å molecular sieve) used for monitoring the reactions was passed through a pipette containing anhydrous $MgSO_4$ and subsequently bubbled with nitrogen for 10 minutes. Column chromatography was carried out using silica gel (60 Å, 70-200 µm, SDS) as stationary phase, and TLC was performed on precoated silica gel on aluminum cards (0.25 mm thick, with fluorescent indicator 254 nm) and observed under UV light. All melting points were determined on a Kofler hot-plate melting point apparatus and are uncorrected. NMR Spectra were recorded on Bruker Avance 300, 400 and 600 MHz instruments.

^1H NMR chemical shifts are reported relative to Me_4Si and were referenced via residual proton resonances of deuterated chloroform, whereas ^{13}C NMR spectra are reported relative to Me_4Si using the carbon signals of the same solvent. Before recording the NMR spectra of samples **6aa** and **7aa** deuterated chloroform was treated with $CaCO_3$ to remove any traces of hydrochloric acid. ^{31}P NMR chemical shifts were externally referenced to 85% H_3PO_4. Coupling constants (*J*) are expressed in Hz. The assignments of the methylene protons of compounds **9** were determined with the aid of $^1H\{^{31}P\}$ NMR. The signals in the ^{13}C NMR spectra were assigned by means of DEPT-135 experiments. All these spectra joined to the $^1H,^1H$-COSY and HMQC spectra of **9ba** and **9da** have been included at the end of the file. On the other hand, the assignments of the carbon atoms in the aromatic rings bonded to the phosphorous atoms in compounds **9** were made based on previous data described in the literature.[1] Abbreviations of coupling patterns are as follows: s, singlet; d, doublet; dd, double doublet t, triplet; m, multiplet. High-resolution mass spectra (HRMS) were recorded on Agilent HPLC 1200/MS TOF 6220, Agilent HPLC 1290 Infinity II/MS Q-TOF 6550 or Agilent 6546 LC/Q-TOF mass spectrometers with ESI sources. The preparation of cyanide **1a** and isocyanides **2b-d** were carried out by experimental procedures previously described in the literature.[2] The notation of the diazaphosphetidines described in this article consist of the number **9** followed by two letters. The first letter is associated with the substituents at the aromatic ring bonded to the isocyanide function (**b**: R^1= R^2= Me; **c**: R^1= Me, R^2= H, **d**: R^1= Cl, R^2= Me) whereas the second one is associated to the substituents at the phosphorous atom (**a**, R^3= R^4= C_6H_5; **b**: R^3= R^4= 3-MeC_6H_4; **c**: R^3= R^4= 4-ClC_6H_4; **d**: R^3= R^4= 3,5-$Me_2C_6H_3$; **e**: R^3= C_6H_5, R^4= 4-MeC_6H_4).

Caution!: Organic azides are potentially hazardous and explosive. Appropriate protective measures should always be applied when handling these chemicals. At the azidation stages, complete removal of residual halogenated solvent should be in mind. Otherwise, the generation of explosive species such as diazidomethane from dichloromethane is possible.

2. Synthesis of *N*-(triphenylphosphoranylidene)-4*H*-benzo[*f*][1,2,3]triazolo[1,5-*a*][1,4]diazepin-6-amine (7aa).

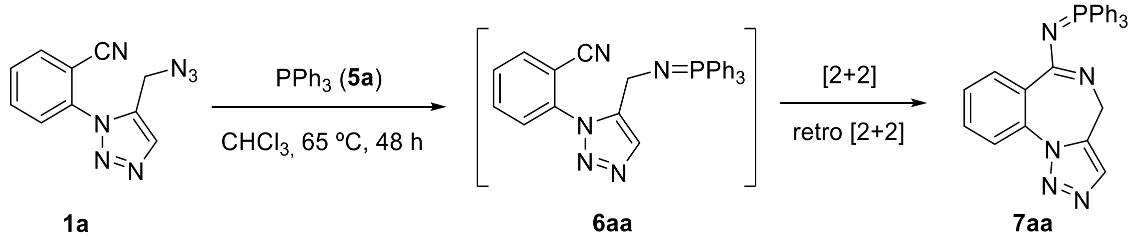

A mixture of 2-[5-(azidomethyl)-1*H*-1,2,3-triazol-1-yl]benzonitrile (**1a**) (0.15 g, 0.67 mmol, 1 equiv) and triphenylphosphine (0.19 g, 0.7 mmol, 1.1 equiv) in anhydrous $CHCl_3$ (100 mL) was heated at 65 ºC, in an oil bath, for 48 h. After cooling to room temperature, the solvent was removed under reduced pressure. The obtained residue was purified by silica-gel (deactivated with 5% Et_3N in hexane) column chromatography (Et_2O, R_f= 0.5) to give the titled compound as a white solid, which was crystallized from Et_2O (white prisms, 0.18 g, 59%); mp 186-188 ºC; ^{1}H NMR (400 MHz, $CDCl_3$, 298 K): δ = 8.37 (dd, *J*= 7.7, 1.6 Hz, 1 H, H_{Ar}), 7.95 (dd, *J*= 7.9, 1.4 Hz, 1 H, H_{Ar}), 7.77-7.69 (m, 6 H, H_m), 7.57 (td, *J*= 7.7, 1.7 Hz, 1 H, H_{Ar}), 7.54-7.47 (m, 4 H, H_p + H_{Ar}), 7.44-7.36 (m, 7 H, H_o + H_{Ar}), 4.13 (s, 2 H, CH_2); ^{13}C{^{1}H} NMR (100 MHz, $CDCl_3$, 298 K): δ = 165.5 (d, $^2J_{CP}$= 5.8 Hz, C), 139.4 (C), 134.3 (d, $^3J_{CP}$= 2.1 Hz, C), 133.1 (d, $^2J_{CP}$= 9.6 Hz, HC_o-PPh), 131.7 (d, $^4J_{CP}$= 2.8, HC_p-PPh), 131.5 (CH), 130.4 (CH), 130.0 (d, $^1J_{CP}$= 91.4 Hz, C_i-PPh), 129.5 (CH), 128.6 (C), 128.3 (d, $^3J_{CP}$= 12.1 Hz, HC_m-PPh), 127.9 (CH), 122.2 (CH), 40.5 (CH_2); ^{31}P{^{1}H} NMR (162 MHz, $CDCl_3$, 298 K): δ = 13.7; HRMS (ESI) calcd for $C_{28}H_{23}N_5P$ $[M + H]^+$ 460.1686, found 460.1691.

The intermediate **6aa** (2-[5-(triphenylphosphoranylidenaminomethyl)-1*H*-1,2,3-triazol-1-yl]benzonitrile) was detected under the reaction mixture and further characterized by ^{1}H and ^{13}C NMR.

6aa: ^{1}H NMR (400 MHz, $CDCl_3$, 298 K): δ = 7.69-7.63 (m, 2 H, H_{Ar}), 7.61-7.57 (m, 1 H, H_{Ar}), 7.55 (s, 1 H, H_{Ar}), 7.53 (dd, *J*= 7.7, 1.3 Hz, 1 H, H_{Ar}), 7.51-7.35 (m, 15 H, Ph_3P), 4.39 (d, $^3J_{HP}$= 17.9 Hz, 2 H, CH_2); ^{13}C{^{1}H} NMR (100 MHz, $CDCl_3$, 298 K): δ = 142.7 (d, $^3J_{CP}$= 20.8 Hz, C), 139.2 (C), 133.4 (CH), 133.2 (CH), 132.6 (CH), 132.2 (d, $^2J_{CP}$= 9.2 Hz, HC_o-PPh), 131.6 (d, $^4J_{CP}$= 2.7, HC_p-PPh), 130.2 (d, $^1J_{CP}$= 96.5 Hz, C_i-PPh), 129.5 (CH), 128.5 (d, $^3J_{CP}$= 11.6 Hz, HC_m-PPh), 128.0 (CH), 115.2 (CN), 110.8 (*C*-CN), 38.6 (d, $^2J_{CP}$= 2.2 Hz, CH_2); ^{31}P{^{1}H} NMR (162 MHz, $CDCl_3$, 298 K): δ = 11.3.

3. General procedure for the preparation of fused diazaphosphetidines 9.

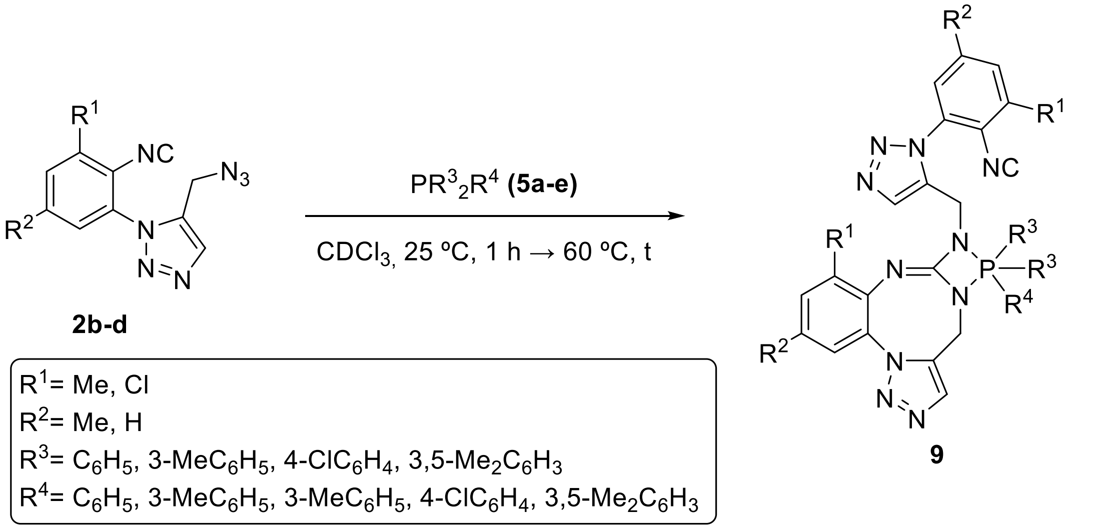

The corresponding azidomethyltriazole **2** (0.4 mmol, 1 equiv) and phosphine **5** (0.4 mmol, 1 equiv) were introduced into a Schlenk flask and successive vacuum and nitrogen cycles were performed. $CDCl_3$ (3 mL) was added, and the reaction mixture stirred until no nitrogen evolution was observed (~ 1 h). The reaction mixture was agitated at 60 ºC, in an oil bath, for the time specified in each example. After this time, anhydrous Et_2O was added until the formation of a small amount of an amorphous precipitate, which was removed by filtration. The filtrate was then concentrated under reduced pressure to a volume of 1-2 mL, and anhydrous Et_2O was slowly added with stirring until the formation of a white precipitate. After cooling, the final mixture was kept at 0 ºC for 1 h, and the white precipitate was filtered and dried to give the corresponding diazaphosphetidine **9**.

*Diazaphosphetidine **9ba*** (R^1= R^2= CH_3, R^3=R^4= C_6H_5).

Prepared from **2b** (101 mg) and **5a** (105 mg). Reaction time: 36 h. Colorless prisms (134 mg, 94%); mp 183-185 ºC; ^{1}H NMR (300 MHz, $CDCl_3$, 298 K): δ = 7.53-7.45 (m, 3 H, H_{Ar}), 7.31-7.21 (m, 13 H, H_{Ar}), 7.12 (s, 1 H, H_{Ar}), 7.10 (s, 1 H, H_{Ar}), 6.84 (s, 1 H, H_{Ar}), 6.77 (s, 1 H, H_{Ar}), 5.99 (s, 1 H, H_{Ar}), 4.42 (dd, $^3J_{HP}$= 20.2 Hz, $^2J_{HH}$= 17.0 Hz, 1 H, CH_2), 4.13-3.92 (m, 2 H, CH_2), 3.26 (dd, $^2J_{HH}$= 16.0 Hz, $^3J_{HP}$= 7.4 Hz, 1 H, CH_2), 2.28 (s, 3 H, CH_3), 2.27 (s, 3 H, CH_3), 2.25 (s, 3 H, CH_3), 2.12 (s, 3 H, CH_3); $^{13}C\{^1H\}$ NMR (150 MHz, $CDCl_3$, 298 K): δ = 171.7 (NC), 146.4 (d, $^2J_{CP}$= 7.8 Hz, C), 140.2 (C), 138.5 (C), 137.5 (C), 136.9 (d, $^1J_{CP}$= 105.8 Hz, C_i-PPh), 136.5 (C), 134.9 (C), 134.0 (C), 133.0 (CH), 132.8 (CH), 132.4 (CH), 132.1 (d, $^2J_{CP}$= 10.6 Hz, HC_o-PPh), 131.7 (C), 131.5 (C), 130.6 (d, $^4J_{CP}$= 1.4 Hz, HC_p-PPh), 130.3 (CH), 128.6 (d, $^3J_{CP}$= 13.0 Hz, HC_m-PPh), 127.3 (C), 125.9 (CH), 125.6 (CH), 121.0 (*C*-NC), 35.6 (CH_2), 35.5 (CH_2), 21.2 (CH_3), 20.3 (CH_3), 19.3 (CH_3), 18.4 (CH_3); $^{31}P\{^1H\}$ NMR (121.5 MHz, $CDCl_3$, 298 K): δ = - 55.7; HRMS (ESI) calcd for $C_{42}H_{38}N_{10}P$ $[M + H]^+$ 713.3013, found 713.3014.

The following synthetic procedure can be conducted to scale up the preparation of diazaphosphetidine **9ba** by starting from 1 mmol of **2b**:

The azidomethyltriazole **2b** (0.253 g, 1.0 mmol) and triphenylphosphine **5a** (0.262 g, 1.0 mmol) were introduced into a Schlenk flask and successive vacuum and nitrogen cycles were performed. $CDCl_3$ (7 mL) was added, and the reaction mixture stirred until no nitrogen evolution was observed (~ 1 h). The reaction mixture was agitated for 36 h more at 60 ºC in an oil bath. After this time, anhydrous Et_2O was added until the formation of an amorphous precipitate, which was removed by filtration. The filtrate was then concentrated under reduced pressure to a volume of 2-3 mL, and anhydrous Et_2O was slowly added with stirring until the formation of a white precipitate. After cooling, the final mixture was kept at 0 ºC for 1 h, and the white precipitate was filtered and dried to give the corresponding diazaphosphetidine **9ba** (0.331 g, 93%).

*Diazaphosphetidine **9bb*** (R^1= R^2= CH_3, R^3= R^4= 3-MeC_6H_4).

Prepared from **2b** (101 mg) and **5b** (121.6 mg). Reaction time: 32 h. Colorless prisms (112 mg, 74%); mp 154-156 ºC; ^{1}H NMR (600 MHz, $CDCl_3$, 298 K): δ = 7.33 (s, 1 H, H_{Ar}), 7.30-7.27 (m, 3 H, H_{Ar}), 7.26-7.22 (m, 3 H, H_{Ar}), 7.13-7.04 (m, 8 H, H_{Ar}), 6.84 (s, 1 H, H_{Ar}), 6.71 (s, 1 H, H_{Ar}), 6.02 (s, 1 H, H_{Ar}), 4.39 (dd, $^3J_{HP}$= 20.5 Hz, $^2J_{HH}$= 17.1 Hz, 1 H, CH_2), 3.99 (dd, $^2J_{HH}$= 16.0 Hz, $^3J_{HP}$= 10.4 Hz, 1 H, CH_2), 3.90 (t, $^2J_{HH}$= $^3J_{HP}$= 16.3 Hz, 1 H, CH_2), 3.28 (dd, $^2J_{HH}$= 16.0 Hz, $^3J_{HP}$= 7.1 Hz, 1 H, CH_2), 2.273 (s, 15 H, CH_3), 2.268 (s, 3 H, CH_3), 2.09 (s, 3 H, CH_3); $^{13}C\{^1H\}$ NMR (150 MHz, $CDCl_3$, 298 K): δ = 171.7 (NC), 146.7 (d, $^2J_{CP}$= 7.8 Hz, C), 140.2 (C), 138.7 (C), 138.4 (d, $^3J_{CP}$= 12.8 Hz, C_m-PAr), 137.9 (C), 136.7 (d, $^1J_{CP}$= 103.5 Hz, C_i-PAr), 136.4 (C), 135.0 (C), 134.0 (C), 132.9 (CH), 132.8 (CH), 132.44 (d, $^2J_{CP}$= 10.2 Hz, HC_o-PAr), 132.38 (CH), 131.8 (C), 131.4 (C), 131.3 (CH), 130.3 (CH), 129.3 (d, $^2J_{CP}$= 10.6 Hz, HC_o-PAr), 128.3 (d, $^3J_{CP}$= 14.0 Hz, HC_m-PAr), 127.4 (C), 125.9 (CH), 125.5 (CH), 120.9 (*C*-NC), 35.7 (CH_2), 35.5 (CH_2), 21.7 (CH_3), 21.1 (CH_3), 20.3 (CH_3), 19.3 (CH_3), 18.5 (CH_3); $^{31}P\{^1H\}$ NMR (243 MHz, $CDCl_3$, 298 K): δ = - 51.7; HRMS (ESI) calcd for $C_{45}H_{44}N_{10}P$ $[M + H]^+$ 755.3483, found 755.3488.

*Diazaphosphetidine **9bd*** (R^1= R^2= CH_3, R^3= R^4= 3,5-$Me_2C_6H_3$).

Prepared from **2b** (101 mg) and **5d** (138.6 mg). Reaction time: 28 h. Colorless prisms (124 mg, 78%); mp 160-162 ºC; ^{1}H NMR (600 MHz, $CDCl_3$, 298 K): δ = 7.38 (s, 1 H, H_{Ar}), 7.11 (s, 1 H, H_{Ar}), 7.09 (s, 4 H, H_{Ar}), 6.88 (s, 3 H, H_{Ar}), 6.86 (s, 3 H, H_{Ar}), 6.85 (s, 1 H, H_{Ar}), 6.65 (s, 1 H, H_{Ar}), 6.06 (s, 1 H, H_{Ar}), 4.36 (dd, $^3J_{HP}$= 20.7 Hz, $^2J_{HH}$= 17.1 Hz, 1 H, CH_2), 3.97 (dd, $^2J_{HH}$= 15.9 Hz, $^3J_{HP}$= 10.2 Hz, 1 H, CH_2), 3.77 (t, $^2J_{HH}$= $^3J_{HP}$= 15.8 Hz, 1 H, CH_2), 3.29 (dd, $^2J_{HH}$= 16.0 Hz, $^3J_{HP}$= 7.0 Hz, 1 H, CH_2), 2.29 (s, 3 H, CH_3), 2.27 (s, 6 H, CH_3), 2.24 (s, 18 H, CH_3), 2.08 (s, 3 H, CH_3); $^{13}C\{^1H\}$ NMR (150 MHz, $CDCl_3$, 298 K): δ = 171.8 (NC), 146.8 (d, $^2J_{CP}$= 8.1 Hz, C), 140.2 (C), 138.9 (C), 138.1 (C), 137.9 (d, $^3J_{CP}$= 13.6 Hz, C_m-PAr), 136.5 (d, $^1J_{CP}$= 103.5 Hz, C_i-PAr), 136.4 (C), 135.1 (C), 134.1 (C), 132.9 (CH), 132.7 (CH), 132.3 (CH), 132.1 (CH), 131.9 (C), 131.2 (C), 130.3 (CH), 129.7 (d, $^2J_{CP}$= 10.2 Hz, HC_o-PAr),

127.4 (C), 125.8 (CH), 125.5 (CH), 121.0 (*C*-NC), 35.7 (CH_2), 35.5 (CH_2), 21.5 (CH_3), 21.1 (CH_3), 20.3 (CH_3), 19.3 (CH_3), 18.5 (CH_3); $^{31}P\{^1H\}$ NMR (243 MHz, $CDCl_3$, 298 K): δ = - 50.0; HRMS (ESI) calcd for $C_{48}H_{50}N_{10}P$ $[M + H]^+$ 797.3952, found 797.3957.

Diazaphosphetidine ***9be*** (R^1= R^2= CH_3, R^3= C_6H_5 R^4= 4-MeC_6H_4).

Prepared from **2b** (101 mg) and **5e** (110.5 mg). Reaction time: 36 h. Colorless prisms (125 mg, 86%); mp 150-152 ºC; ^1H NMR (600 MHz, $CDCl_3$, 298 K): δ = 7.50 (t, *J*= 7.0 Hz, 1 H, H_{Ar}), 7.44 (t, *J*= 7.5 Hz, 1 H, H_{Ar}), 7.40-7.35 (m, 2 H, H_{Ar}), 7.34-7.15 (m, 11 H, H_{Ar}), 7.13-7.09 (m, 2 H, H_{Ar}), 6.83 (s, 1 H, H_{Ar}), 6.76 (s, 1 H, H_{Ar}), 6.00 (s, 1 H, H_{Ar}), 4.41 (dd, $^3J_{HP}$= 20.5 Hz, $^2J_{HH}$= 17.1 Hz, 1 H, CH_2), 4.06-3.92 (m, 2 H, CH_2), 3.26 (dd, $^2J_{HH}$= 16.0 Hz, $^3J_{HP}$= 7.2 Hz, 1 H, CH_2), 2.41 (s, 3 H, CH_3), 2.27 (s, 3 H, CH_3), 2.26 (s, 3 H, CH_3), 2.25 (s, 3 H, CH_3), 2.10 (s, 3 H, CH_3); $^{13}C\{^1H\}$ NMR (150 MHz, $CDCl_3$, 298 K): δ = 171.6 (NC), 146.6 (d, $^2J_{CP}$= 7.8 Hz, C), 141.2 (C), 140.2 (C), 138.6 (C), 138.2 (d, $^1J_{CP}$= 98.3 Hz, C_i-PAr), 137.6 (C), 136.5 (C), 135.9 (d, $^1J_{CP}$= 113.0 Hz, C_i-PAr), 134.9 (C), 134.0 (C), 132.94 (CH), 132.89 (d, $^2J_{CP}$= 10.2 Hz, HC_o-PAr), 132.5 (CH), 131.9 (d, $^2J_{CP}$= 10.4 Hz, HC_o-PAr), 131.6 (C), 131.49 (C), 131.46 (d, $^2J_{CP}$= 8.7 Hz, HC_o-PAr), 130.5 (CH), 130.3 (CH), 129.2 (d, $^3J_{CP}$= 13.6 Hz, HC_m-PAr), 128.7 (d, $^3J_{CP}$= 13.2 Hz, HC_m-PAr), 128.53 (CH), 128.45 (CH), 128.4 (d, $^3J_{CP}$= 12.5 Hz, HC_m-PAr), 127.3 (C), 125.8 (CH), 125.5 (CH), 120.9 (*C*-NC), 35.7 (CH_2), 35.5 (CH_2), 21.4 (CH_3), 21.2 (CH_3), 20.3 (CH_3), 19.3 (CH_3), 18.5 (CH_3), only two quaternary carbon atoms C*i* were observed; $^{31}P\{^1H\}$ NMR (243 MHz, $CDCl_3$, 298 K): δ = - 52.9. After 6 h in solution a small amount of the phosphine oxide (29.5 ppm) was observed; HRMS (ESI) calcd for $C_{43}H_{40}N_{10}P$ $[M + H]^+$ 727.3170, found 727.3182.

Diazaphosphetidine ***9ca*** (R^1= CH_3, R^2= H, R^3=R^4= C_6H_5).

Prepared from **2c** (95.6 mg) and **5a** (105 mg). Reaction time: 20 h. Colorless prisms (109.6 mg, 80%); mp 178-180 ºC; ^1H NMR (400 MHz, $CDCl_3$, 298 K): δ = 7.52-7.45 (m, 3 H, H_{Ar}), 7.40-7.32 (m, 6 H, H_{Ar}), 7.30-7.21 (m, 8 H, H_{Ar}), 7.17 (d, $^3J_{HH}$= 7.7 Hz, 1 H, H_{Ar}), 6.99 (d, $^3J_{HH}$= 7.8 Hz, 1 H, H_{Ar}), 6.97-6.88 (m, 3 H, H_{Ar}), 6.00 (s, 1 H, H_{Ar}), 4.42-4.20 (m, 2 H, CH_2), 3.96 (dd, $^2J_{HH}$= 16.0 Hz, $^3J_{HP}$= 10.4 Hz, 1 H, CH_2), 3.26 (dd, $^2J_{HH}$= 16.0 Hz, $^3J_{HP}$= 7.2 Hz, 1 H, CH_2), 2.25 (s, 3 H, CH_3), 2.15 (s, 3H, CH_3); $^{13}C\{^1H\}$ NMR (75 MHz, $CDCl_3$, 298 K): δ = 172.0 (NC), 146.3 (d, $^2J_{CP}$= 8.2 Hz, C), 141.1 (C), 137.3 (C), 137.2 (C), 136.7 (C), 136.6 (d, $^1J_{CP}$= 105.8 Hz, C_i-PPh), 134.8 (C), 133.9 (C), 133.3 (CH), 132.0 (CH), 131.9 (d, $^2J_{CP}$= 10.6 Hz, HC_o-PPh), 131.2 (CH), 130.5 (d, $^4J_{CP}$= 2.5 Hz, HC_p-PPh), 130.2 (CH), 129.5 (CH), 128.5 (d, $^3J_{CP}$= 13.1 Hz, HC_m-PPh), 127.4 (C), 125.24 (CH), 125.16 (CH), 122.3 (*C*-NC), 121.7 (CH), 35.3 (CH_2), 35.2 (CH_2), 19.3 (CH_3), 18.4 (CH_3); $^{31}P\{^1H\}$ NMR (162 MHz, $CDCl_3$, 298 K): δ = - 53.3; HRMS (ESI) calcd for $C_{40}H_{34}N_{10}P$ $[M + H]^+$ 685.2700, found 685.2706.

Diazaphosphetidine ***9cb*** (R^1= CH_3, R^2= H, R^3= R^4= 3-MeC_6H_4).

Prepared from **2c** (95.6 mg) and **5b** (121.6 mg). Reaction time: 12 h. Colorless prisms (94.5 mg, 65%); mp 171-173 ºC; ^1H NMR (600 MHz, $CDCl_3$, 298 K): δ = 7.31-7.22 (m, 8 H, H_{Ar}), 7.16 (d, $^3J_{HH}$=

7.7 Hz, 1 H, H_{Ar}), 7.07-7.00 (m, 7 H, H_{Ar}), 6.96 (s, 1 H, H_{Ar}), 6.94 (t, $^3J_{HH}$= 7.6 Hz, 1 H, H_{Ar}), 6.89 (d, $^3J_{HH}$= 7.6 Hz, 1 H, H_{Ar}), 6.07 (s, 1 H, H_{Ar}), 4.29 (d, $^3J_{HP}$= 17.0 Hz, 2 H, CH_2), 3.94 (dd, $^2J_{HH}$= 16.0 Hz, $^3J_{HP}$= 10.3 Hz, 1 H, CH_2), 3.27 (dd, $^2J_{HH}$= 16.0 Hz, $^3J_{HP}$= 7.1 Hz, 1 H, CH_2), 2.29 (s, 9 H, CH_3), 2.27 (s, 3 H, CH_3), 2.15 (s, 3 H, CH_3); $^{13}C\{^1H\}$ NMR (150 MHz, $CDCl_3$, 298 K): δ = 172.0 (NC), 146.6 (d, $^2J_{CP}$= 8.1 Hz, C), 141.3 (C), 138.4 (d, $^3J_{CP}$= 12.9 Hz, C_m-PAr), 137.5 (C), 136.64 (d, $^1J_{CP}$= 105.4 Hz, C_i-PAr), 136.61 (C), 135.0 (C), 134.1 (C), 133.4 (CH), 132.4 (d, $^2J_{CP}$= 10.7 Hz, HC_o-PAr), 131.9 (CH), 131.3 (2 x CH), 131.2 (CH), 130.3 (CH), 129.6 (CH), 129.2 (d, $^2J_{CP}$= 10.8 Hz, HC_o-PAr), 128.3 (d, $^3J_{CP}$= 14.0 Hz, HC_m-PAr), 127.5 (C), 125.3 (d, $^4J_{CP}$= 6.7 Hz, HC_p-PAr), 123.5 (*C*-NC), 121.5 (CH), 35.4 (CH_2), 35.3 (CH_2), 21.7 (CH_3), 19.4 (CH_3), 18.5 (CH_3), the resonance of one quaternary carbon atom was not observed; $^{31}P\{^1H\}$ NMR (162 MHz, $CDCl_3$, 298 K): δ = - 51.3; HRMS (ESI) calcd for $C_{43}H_{40}N_{10}P$ $[M + H]^+$ 727.3170, found 727.3173.

Diazaphosphetidine ***9cd*** (R^1= CH_3, R^2= H, R^3= R^4= 3,5-$Me_2C_6H_3$).

Prepared from **2c** (95.6 mg) and **5d** (138.6 mg). Reaction time: 15 h. Colorless prisms (150.8 mg, 93%); mp 182-184 ºC; ^1H NMR (600 MHz, $CDCl_3$, 298 K): δ = 7.27 (d, *J*= 7.4 Hz, 1 H, H_{Ar}), 7.24 (d, *J*= 7.9 Hz, 1 H, H_{Ar}), 7.16 (d, *J*= 7.9 Hz, 1 H, H_{Ar}), 7.10 (s, 3 H, H_{Ar}), 7.03 (dd, *J*= 7.9, 1.1 Hz, 1 H, H_{Ar}), 6.98 (s, 1 H, H_{Ar}), 6.93 (t, *J*= 7.7 Hz, 1 H, H_{Ar}), 6.87-6.83 (m, 4 H, H_{Ar}), 6.82 (s, 3 H, H_{Ar}), 6.16 (s, 1 H, H_{Ar}), 4.31-4.19 (m, 2 H, CH_2), 3.92 (dd, $^2J_{HH}$= 16.0 Hz, $^3J_{HP}$= 10.2 Hz, 1 H, CH_2), 3.28 (dd, $^2J_{HH}$= 16.0 Hz, $^3J_{HP}$= 7.0 Hz, 1 H, CH_2), 2.27 (s, 3 H, CH_3), 2.26 (s, 18 H, CH_3), 2.17 (s, 3 H, CH_3); $^{13}C\{^1H\}$ NMR (150 MHz, $CDCl_3$, 298 K): δ = 172.9 (NC), 147.7 (d, $^2J_{CP}$= 8.1 Hz, C), 142.5 (C), 139.0 (d, $^3J_{CP}$= 13.6 Hz, C_m-PAr), 138.8 (C), 137.6 (C), 137.5 (d, $^1J_{CP}$= 105.0 Hz, C_i-PAr), 136.0 (C), 135.3 (C), 134.4 (CH), 133.4 (C), 133.1 (2 x CH), 132.9 (CH), 132.2 (CH), 131.4 (CH), 130.6 (d, $^2J_{CP}$= 10.5 Hz, HC_o-PAr), 130.5 (CH), 128.5 (C), 126.3 (CH), 124.6 (*C*-NC), 122.3 (CH), 36.4 (CH_2), 36.3 (CH_2), 22.6 (CH_3), 20.5 (CH_3), 19.5 (CH_3); $^{31}P\{^1H\}$ NMR (243 MHz, $CDCl_3$, 298 K): δ = - 49.4; HRMS (ESI) calcd for $C_{46}H_{46}N_{10}P$ $[M + H]^+$ 769.3639, found 769.3644.

Diazaphosphetidine ***9da*** (R^1= Cl, R^2= CH_3, R^3=R^4= C_6H_5).

Prepared from **2d** (109 mg) and **5a** (105 mg). Reaction time: 1.75 h. Colorless prisms (128 mg, 85%); mp 184-186 ºC; ^1H NMR (400 MHz, $CDCl_3$, 298 K): δ = 7.54-7.48 (m, 3 H, H_{Ar}), 7.42-7.35 (m, 6 H, H_{Ar}), 7.34-7.24 (m, 8 H, H_{Ar}), 7.02 (s, 1 H, H_{Ar}), 6.98 (s, 1 H, H_{Ar}), 6.91 (d, $^4J_{HH}$= 4 Hz, 1 H, H_{Ar}), 6.04 (s, 1 H, H_{Ar}), 4.50 (t, $^2J_{HH}$= $^3J_{HP}$= 16.6 Hz, 1 H, CH_2), 4.27 (t, $^2J_{HH}$= $^3J_{HP}$= 16.6 Hz, 1 H, CH_2), 3.94 (dd, $^2J_{HH}$= 16.0 Hz, $^3J_{HP}$= 9.3 Hz, 1 H, CH_2), 3.26 (dd, $^2J_{HH}$= 16.1 Hz, $^3J_{HP}$= 6.4 Hz, 1 H, CH_2), 2.28 (s, 3 H, CH_3), 2.25 (s, 3 H, CH_3); $^{13}C\{^1H\}$ NMR (100 MHz, $CDCl_3$, 298 K): δ = 174.2 (NC), 147.6 (d, $^2J_{CP}$= 8.1 Hz, C), 141.5 (C), 137.6 (C), 137.1 (C), 136.1 (d, $^1J_{CP}$= 106.6 Hz, C_i-PPh), 134.0 (C), 133.5 (CH), 133.3 (C), 132.1 (C), 132.0 (d, $^2J_{CP}$= 10.7 Hz, HC_o-PPh), 131.7 (CH), 131.6 (CH), 131.5 (C), 131.0 (C), 130.8 (d, $^4J_{CP}$= 2.8 Hz, HC_p-PPh), 130.5 (CH), 128.7 (d, $^3J_{CP}$= 13.2 Hz, HC_m-PPh), 128.5 (C), 127.4 (CH), 126.5 (CH), 120.2 (*C*-NC), 35.4 (CH_2), 35.2 (d, $^2J_{CP}$= 2.1 Hz, CH_2), 21.2 (CH_3), 20.2 (CH_3);

$^{31}P\{^1H\}$ NMR (121.5 MHz, $CDCl_3$, 298 K): δ = - 52.0; HRMS (ESI) calcd for $C_{40}H_{32}Cl_2N_{10}P$ [M + H]$^+$ 753.1921, found 753.1925.

Diazaphosphetidine ***9db*** (R^1= Cl, R^2= CH_3, R^3= R^4= 3-$H_3CC_6H_4$).

Prepared from **2d** (109 mg) and **5b** (121.6 mg). Reaction time: 1.5 h. Colorless prisms (114.6 mg, 72%); mp 135-137 ºC; ^1H NMR (400 MHz, $CDCl_3$, 298 K): δ = 7.35-7.23 (m, 8 H, H_{Ar}), 7.12-6.98 (m, 8 H, H_{Ar}), 6.91 (dd, J= 2.0, 0.6 Hz, 1 H, H_{Ar}), 6.09 (s, 1 H, H_{Ar}), 4.46 (t, $^2J_{HH}$= $^3J_{HP}$= 17.7 Hz, 1 H, CH_2), 4.22 (t, $^2J_{HH}$= $^3J_{HP}$= 16.4 Hz, 1 H, CH_2), 3.92 (dd, $^2J_{HH}$= 16.0 Hz, $^3J_{HP}$= 9.1 Hz, 1 H, CH_2), 3.27 (dd, $^2J_{HH}$= 16.0 Hz, $^3J_{HP}$= 6.2 Hz, 1 H, CH_2), 2.29 (s, 9 H, CH_3), 2.28 (s, 3 H, CH_3), 2.26 (s, 3 H, CH_3); $^{13}C\{^1H\}$ NMR (100 MHz, $CDCl_3$, 298 K): δ = 174.3 (NC), 147.8 (d, $^2J_{CP}$= 8.3 Hz, C), 141.6 (C), 138.6 (d, $^3J_{CP}$= 13.0 Hz, C_m-PAr), 137.8 (C), 137.4 (C), 136.1 (d, $^1J_{CP}$= 106.1 Hz, C_i-PAr), 134.2 (C), 133.6 (CH), 133.5 (C), 132.3 (d, $^2J_{CP}$= 10.7 Hz, HC_o-PAr), 131.9 (C), 131.61 (CH), 131.56 (CH), 131.54(CH), 131.50 (C), 131.0 (C), 130.6 (CH), 129.2 (d, $^2J_{CP}$= 11.1 Hz, HC_o-PAr), 128.6 (C), 128.5 (d, $^3J_{CP}$= 13.2 Hz, HC_m-PAr), 127.4 (CH), 126.5 (CH), 120.1 (*C*-NC), 35.5 (CH_2), 35.3 (CH_2), 21.7 (CH_3), 21.2 (CH_3), 20.2 (CH_3); $^{31}P\{^1H\}$ NMR (162 MHz, $CDCl_3$, 298 K): δ = - 47.6; HRMS (ESI) calcd for $C_{43}H_{38}Cl_2N_{10}P$ [M + H]$^+$ 795.2390, found 795.2399.

Diazaphosphetidine ***9dc*** (R^1= Cl, R^2= CH_3, R^3= R^4= 4-ClC_6H_4).

Prepared from **2d** (109 mg) and **5c** (146.2 mg). Reaction time: 4 h. Colorless prisms (68.6 mg, 40%); mp 155-157 ºC; ^1H NMR (400 MHz, $CDCl_3$, 298 K): δ = 7.43-7.37 (m, 6 H, H_{Ar}), 7.36-7.34 (m, 1H, H_{Ar}), 7.30-7.28 (m, 1 H, H_{Ar}), 7.23-7.15 (m, 6 H, H_{Ar}), 7.14-7.11 (m, 1 H, H_{Ar}), 7.02-6.99 (m, 1 H, H_{Ar}), 6.95-6.93 (m, 1 H, H_{Ar}), 6.19 (s, 1 H, H_{Ar}), 4.38 (t, $^2J_{HH}$= $^3J_{HP}$= 17.7 Hz, 1 H, CH_2), 4.24 (t, $^2J_{HH}$= $^3J_{HP}$= 17.6 Hz, 1 H, CH_2), 3.96 (dd, $^2J_{HH}$= 16.1 Hz, $^3J_{HP}$= 10.6 Hz, 1 H, CH_2), 3.24 (dd, $^2J_{HH}$= 16.1 Hz, $^3J_{HP}$= 7.4 Hz, 1 H, CH_2), 2.30 (s, 3 H, CH_3), 2.28 (s, 3 H, CH_3); $^{13}C\{^1H\}$ NMR (100 MHz, $CDCl_3$, 298 K): δ = 174.8 (NC), 146.9 (d, $^2J_{CP}$= 7.8 Hz, C), 141.7 (C), 137.73 (d, $^4J_{CP}$= 3.4 Hz, C_p-PAr), 136.9 (C), 136.8 (C), 134.4 (d, $^1J_{CP}$= 108.0 Hz, C_i-PAr), 133.6 (C), 133.31 (CH), 133.25 (d, $^2J_{CP}$= 11.9 Hz, HC_o-PAr), 133.1 (C), 132.8 (C), 131.9 (CH), 131.81 (CH), 131.75 (C), 130.9 (C), 130.5 (CH), 129.3 (d, $^3J_{CP}$= 14.0 Hz, HC_m-PAr), 128.3 (C), 127.2 (CH), 126.7 (CH), 120.1 (*C*-NC), 35.4 (CH_2), 35.3 (d, $^2J_{CP}$= 1.9 Hz, CH_2), 21.2 (CH_3), 20.2 (CH_3); $^{31}P\{^1H\}$ NMR (162 MHz, $CDCl_3$, 298 K): δ = - 57.6; HRMS (ESI) calcd for $C_{40}H_{29}Cl_5N_{10}P$ [M + H]$^+$ 855.0751, found 855.0757.

Diazaphosphetidine ***9dd*** (R^1= Cl, R^2= CH_3, R^3= R^4= 3,5-$Me_2C_6H_3$).

Prepared from **2d** (109 mg) and **5d** (138.6 mg). Reaction time: 1 h. Colorless prisms (159 mg, 95%); mp 158-160 ºC; ^1H NMR (600 MHz, $CDCl_3$, 298 K): δ = 7.32 (d, J= 1.5 Hz, 1 H, H_{Ar}), 7.24 (s, 1 H, H_{Ar}), 7.12 (s, 3 H, H_{Ar}), 7.03 (s, 1 H, H_{Ar}), 6.99 (s, 1 H, H_{Ar}), 6.92 (d, J= 1.2 Hz, 1 H, H_{Ar}), 6.87 (s, 3 H, H_{Ar}), 6.85 (s, 3 H, H_{Ar}), 6.16 (s, 1 H, H_{Ar}), 4.41 (t, $^2J_{HH}$= $^3J_{HP}$= 17.1 Hz, 1 H, CH_2), 4.18 (t, $^2J_{HH}$= $^3J_{HP}$= 16.2 Hz, 1 H, CH_2), 3.90 (dd, $^2J_{HH}$= 16.0 Hz, $^3J_{HP}$= 8.8 Hz, 1 H, CH_2), 3.28 (dd, $^2J_{HH}$= 16.0 Hz, $^3J_{HP}$= 5.9 Hz,

1 H, CH_2), 2.29 (s, 3 H, CH_3), 2.26 (s, 21 H, CH_3); $^{13}C\{^1H\}$ NMR (150 MHz, $CDCl_3$, 298 K): δ = 174.2 (NC), 148.0 (d, $^2J_{CP}$= 8.1 Hz, C), 141.6 (C), 138.1 (d, $^3J_{CP}$= 13.7 Hz, C_m-PAr), 138.0 (C), 137.59 (C), 135.8 (d, $^1J_{CP}$= 105.8 Hz, C_i-PAr), 134.3 (C), 133.6 (CH), 132.4 (CH), 131.6 (C), 131.53 (CH), 131.46 (CH), 131.4 (C), 131.0 (C), 130.6 (CH), 129.5 (d, $^2J_{CP}$= 10.5 Hz, HC_o-PAr), 128.7 (C), 127.4 (CH), 126.5 (CH), 125.0 (*C*-NC), 120.1 (C), 35.5 (CH_2), 35.3 (CH_2), 21.5 (CH_3), 21.2 (CH_3), 20.2 (CH_3); $^{31}P\{^1H\}$ NMR (243 MHz, $CDCl_3$, 298 K): δ = - 45.0; HRMS (ESI) calcd for $C_{46}H_{44}Cl_2N_{10}P$ $[M + H]^+$ 837.2860, found 837.2865.

Diazaphosphetidine ***9de*** (R^1= Cl, R^2= CH_3, R^3= C_6H_5, R^4= 4-MeC_6H_4).

Prepared from **2d** (109 mg) and **5e** (110.5 mg). Reaction time: 1.75 h. Colorless prisms (136.6 mg, 89%); mp 149-151 ºC; ^1H NMR (600 MHz, $CDCl_3$, 298 K): δ = 7.56-7.45 (m, 2 H, H_{Ar}), 7.44-7.314 (m, 6 H, H_{Ar}), 7.31-7.28 (m, 1H, H_{Ar}), 7.28-7.24 (m, 2 H, H_{Ar}), 7.24-7.18 (m, 5 H, H_{Ar}), 7.03 (s, 1 H, H_{Ar}), 7.01 (s, 1 H, H_{Ar}), 6.91 (d, *J*= 1.8 Hz, 1 H, H_{Ar}), 6.05 (s, 1 H, H_{Ar}), 4.45 (t, $^2J_{HH}$= $^3J_{HP}$= 17.9 Hz, 1 H, CH_2), 4.26 (t, $^2J_{HH}$= $^3J_{HP}$= 16.7 Hz, 1 H, CH_2), 3.94 (dd, $^2J_{HH}$= 16.0 Hz, $^3J_{HP}$= 9.1 Hz, 1 H, CH_2), 3.26 (dd, $^2J_{HH}$= 16.1 Hz, $^3J_{HP}$= 6.2 Hz, 1 H, CH_2), 2.42 (s, 3 H, CH_3), 2.28 (s, 3 H, CH_3), 2.26 (s, 3 H, CH_3); $^{13}C\{^1H\}$ NMR (100 MHz, $CDCl_3$, 298 K): δ = 174.2 (NC), 147.8 (d, $^2J_{CP}$= 8.1 Hz, C), 141.50 (C), 141.48 (d, $^4J_{CP}$= 3.3 Hz, C_p-PAr), 137.7 (C), 137.21 (d, $^1J_{CP}$= 99.0 Hz, C_i-PAr), 137.17 (C), 135.4 (d, $^1J_{CP}$= 110.9 Hz, C_i-PAr), 134.1 (C), 133.5 (CH), 133.3 (C), 133.0 (C), 132.8 (d, $^2J_{CP}$= 11.4 Hz, HC_o-PAr), 132.0 (C), 131.9 (CH), 131.8 (CH), 131.6 (d, $^2J_{CP}$= 8.9 Hz, HC_o-PAr), 131.53 (C), 131.46 (d, $^2J_{CP}$= 10.8 Hz, HC_o-PAr), 131.0 (C), 130.8 (d, $^4J_{CP}$= 2.8 Hz, HC_p-PAr), 130.6 (d, $^4J_{CP}$= 2.6 Hz, HC_p-PAr), 130.5 (CH), 129.4 (d, $^3J_{CP}$= 13.9 Hz, HC_m-PAr), 128.8 (d, $^3J_{CP}$= 14.1 Hz, HC_m-PAr), 128.6 (d, $^3J_{CP}$= 13.3 Hz, HC_m-PAr), 127.4 (CH), 126.5 (CH), 120.2 (*C*-NC), 35.4 (CH_2), 35.3 (CH_2), 21.4 (CH_3), 21.2 (CH_3), 20.2 (CH_3), only two quaternary carbon atoms C*i* were observed; $^{31}P\{^1H\}$ NMR (162 MHz, $CDCl_3$, 298 K): δ = - 48.9; HRMS (ESI) calcd for $C_{41}H_{34}Cl_2N_{10}P$ $[M + H]^+$ 767.2077, found 767.2085.

4. Synthesis of guanidine 13b.

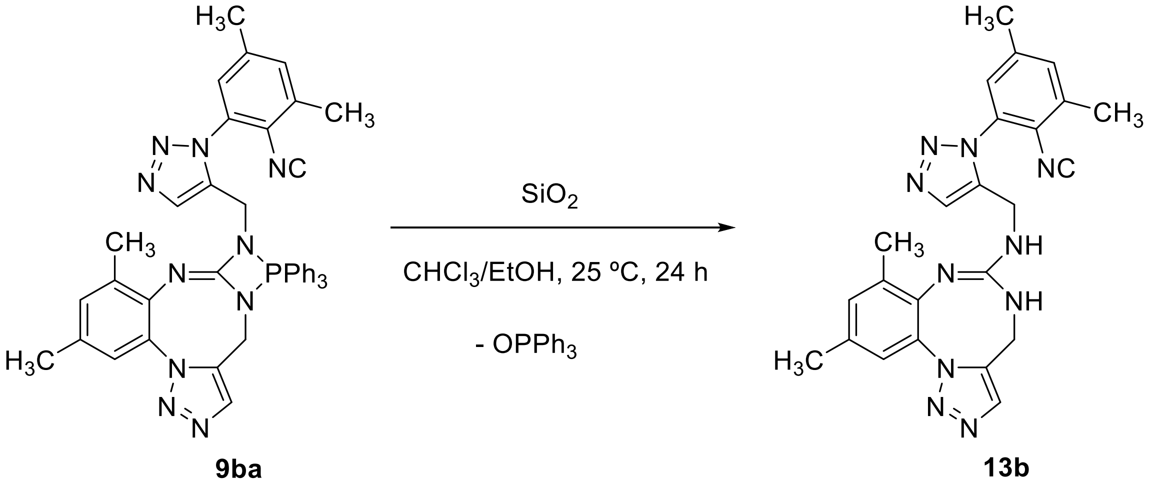

To a solution of the diazaphosphetidine **9ba** (356.4 mg, 0.5 mmol, 1 equiv) in $CHCl_3$/EtOH (1/1 v/v) (30 mL), silica gel (3 g) was added, and the reaction mixture was stirred at 25 ºC for 24 h. The liquid phase was decanted, and the remaining silica gel was stirred with a solution of Et_3N (5 mmol, 0.51 g, 0.7 mL, 10 equiv) in EtOH (20 mL) for 1h. The silica gel was separated by filtration and the combined organic phases were concentrated under reduced pressure. The residue was purified by silica-gel column chromatography (first AcOEt and then 7:3 AcOEt/EtOH) to give the titled compound as a white solid which was crystallized from EtOH/Et_2O (white prisms, 215 mg, 95%); mp 154-156 ºC; ^{1}H NMR (400 MHz, $CDCl_3$, 298 K): δ = 7.59 (s, 1 H, H_{Ar}), 7.56 (s, 1 H, H_{Ar}), 7.18 (s, 1 H, H_{Ar}), 7.08 (s, 1 H, H_{Ar}), 7.01 (s, 1 H, H_{Ar}), 6.96 (s, 1 H, H_{Ar}), 5.70 (br s, 2 H, NH), 4.50-4.32 (m, 2 H, CH_2), 4.22-3.95 (m, 2 H, CH_2), 2.38 (s, 3 H, CH_3), 2.29 (s, 3 H, CH_3), 2.24 (s, 3 H, CH_3), 1.92 (s, 3 H, CH_3); $^{13}C\{^1H\}$ NMR (100 MHz, $CDCl_3$, 298 K): δ = 171.1 (NC), 148.4 (C), 140.6 (C), 139.6 (C), 139.0 (C), 136.4 (C), 135.6 (C), 133.3 (C), 132.9 (CH), 132.5 (CH), 132.4 (CH), 132.0 (C), 131.2 (CH), 131.0 (C), 126.3 (CH), 125.9 (C), 123.8 (CH), 120.6 (C), 35.6 (CH_2), 35.0 (CH_2), 21.1 (CH_3), 20.4 (CH_3), 18.7 (CH_3), 18.6 (CH_3); HRMS (ESI) calcd for $C_{24}H_{25}N_{10}$ $[M + H]^+$ 453.2258, found 453.2260.

5. Synthesis of diazetidine 14b from guanidine 13b.

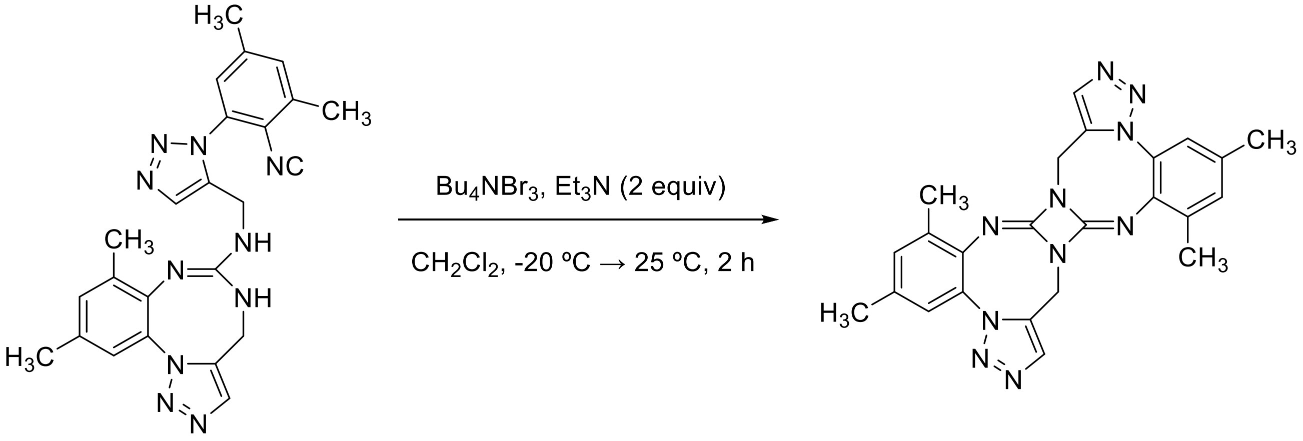

To a solution of the guanidine **13b** (226 mg, 0.5 mmol, 1 equiv) in anhydrous CH_2Cl_2 (20 mL) at -20 ºC, tetrabutylammonium tribromide (241 mg, 0.5 mmol, 1 equiv) was added under N_2. The mixture was stirred for 10 min, and then allowed to reach 0 ºC, and triethylamine (1 mmol, 101 mg, 0.14 mL, 2 equiv) was then added. The reaction mixture was stirred at room temperature for 2 hours, the solvent removed under reduced pressure and the residue purified by silica-gel column chromatography (7:3 AcOEt/hexane) to give the diazetidine **14b** as a white solid (135 mg, 60%); mp > 300 ºC. In solution, a mixture of diastereoisomers is observed in an 87:13 ratio. ^{1}H NMR (300 MHz, $CDCl_3$, 298 K): δ = 7.75 (s, 2 H, H_{Ar}, major), 7.26-7.23 (m, 4 H, 2 H_{Ar}, major and 2 H_{Ar}, minor), 7.12 (s, 2 H, H_{Ar}, minor), 7.05-7.01 (m, 4 H, 2 H_{Ar}, major and 2 H_{Ar}, minor), 4.70 (d, $^2J_{HH}$= 15.7 Hz, 2 H, $CH_A\mathit{H}_B$, major), 4.57 (d, $^2J_{HH}$= 15.8 Hz, 2 H, $CH_A\mathit{H}_B$, minor), 4.03 (dd, $^2J_{HH}$= 15.7, $^4J_{HH}$= 0.5 Hz, 2 H, $C\mathit{H}_AH_B$, major), 3.99 (d, $^2J_{HH}$= 15.3 Hz, 2 H, $C\mathit{H}_AH_B$, minor), 2.37 (s, 12 H, CH_3, major and CH_3, minor), 2.36 (s, 6 H, CH_3, major), 2.33 (s, 6 H, CH_3 minor); ^{13}C{^{1}H} NMR (75 MHz, $CDCl_3$, 298 K): δ = 146.8 (C, minor), 146.6 (C, major), 135.8 (C, major), 135.2 (C, major), 135.1 (C, minor), 133.63 (CH, major), 133.57 (C, minor), 133.38 (C, major), 133.35 (CH, minor), 133.24 (C, major), 133.15 (C, minor), 131.6 (CH, major), 130.5 (CH, minor), 126.5 (CH, major), 126.2 (C, major and CH minor), 126.0 (C, minor), 36.5 (CH_2, minor), 36.0 (CH_2, major), 20.6 (CH_3, major), 18.5 (CH_3, minor), 18.4 (CH_3, major); HRMS (ESI) calcd for $C_{24}H_{23}N_{10}$ $[M + H]^+$ 451.2102, found 451.2103.

6. Synthesis of diazetidine 14b by thermal treatment of the diazaphosphetidine 9ba.

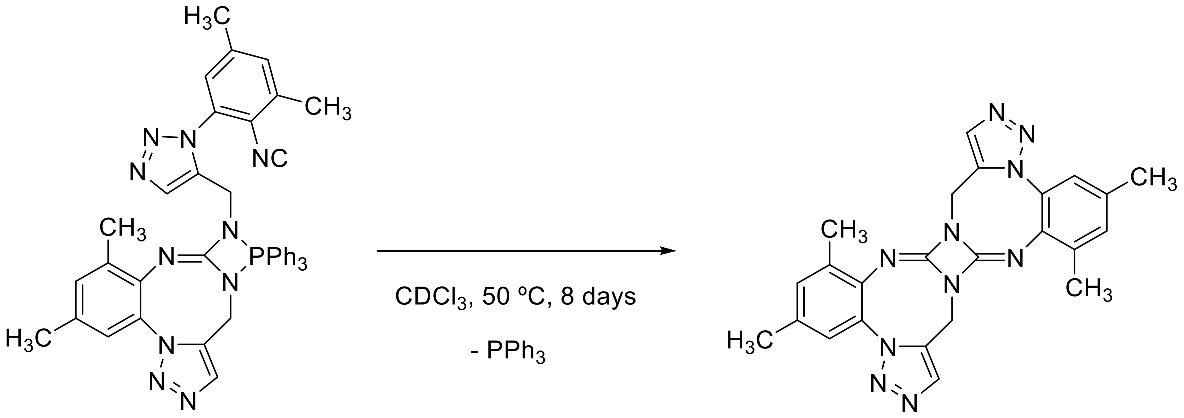

9ba **14b**

A solution of the diazaphosphetidine **9ba** (135 mg, 0.3 mmol) in anhydrous $CDCl_3$ (2 mL) was stirred at 50 ºC, in an oil bath, under N_2 for 8 days. Then, the solvent was removed under reduced pressure and the residue was purified by silica-gel column chromatography (7:3 AcOEt/hexane) to give the diazetidine **14b** as a white solid (111 mg, 82%).

7. $^{31}P\{^1H\}$ NMR monitoring of the reaction mixture of 5-azidomethyltriazole 1a with PPh_3 (5a).

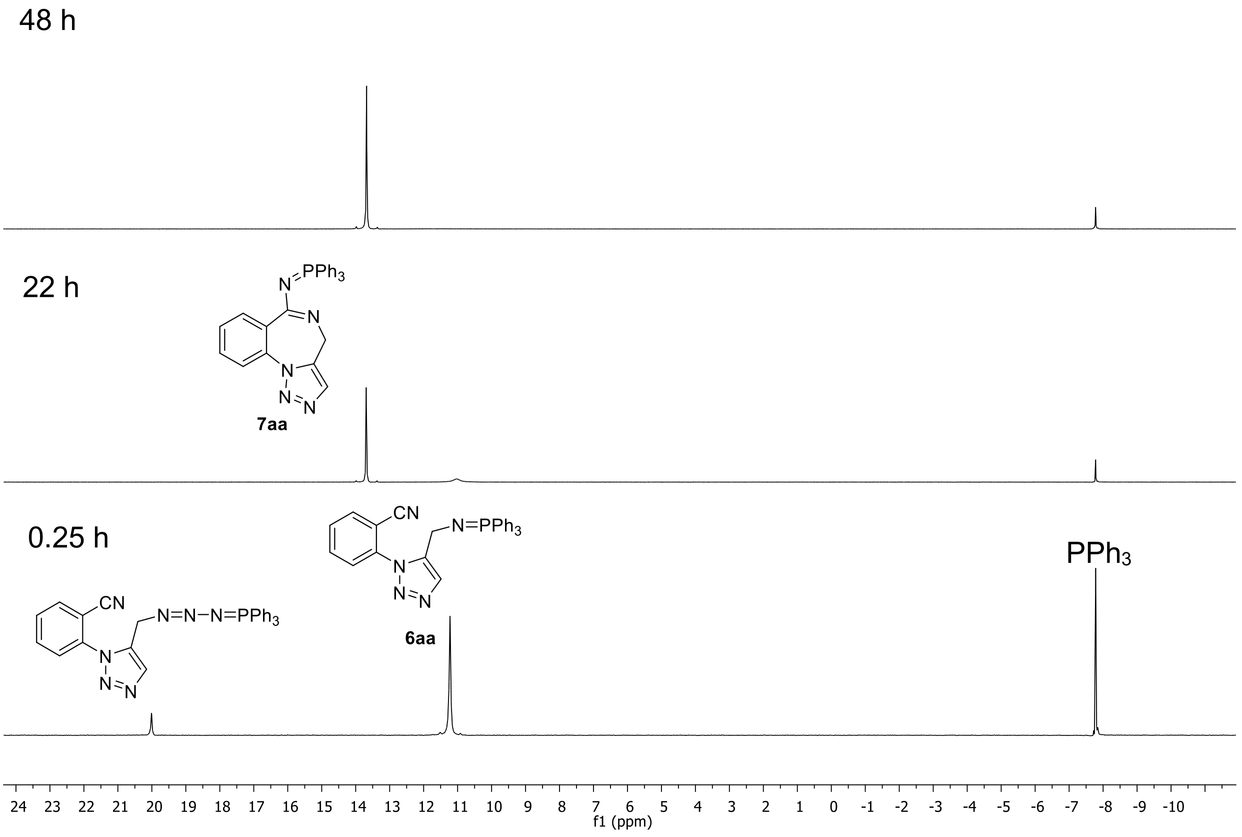

Figure S1. $^{31}P\{^1H\}$ NMR spectra (162 MHz) of the reaction mixture of 5-azidomethyltriazole **1a** with PPh_3 (1.1 equiv) in $CDCl_3$ at 60 ºC at different reaction times.

8. ^{1}H and $^{31}P\{^{1}H\}$ NMR monitoring of the reaction mixture of 5-azidomethyltriazole 2b with PPh_3 (5a).

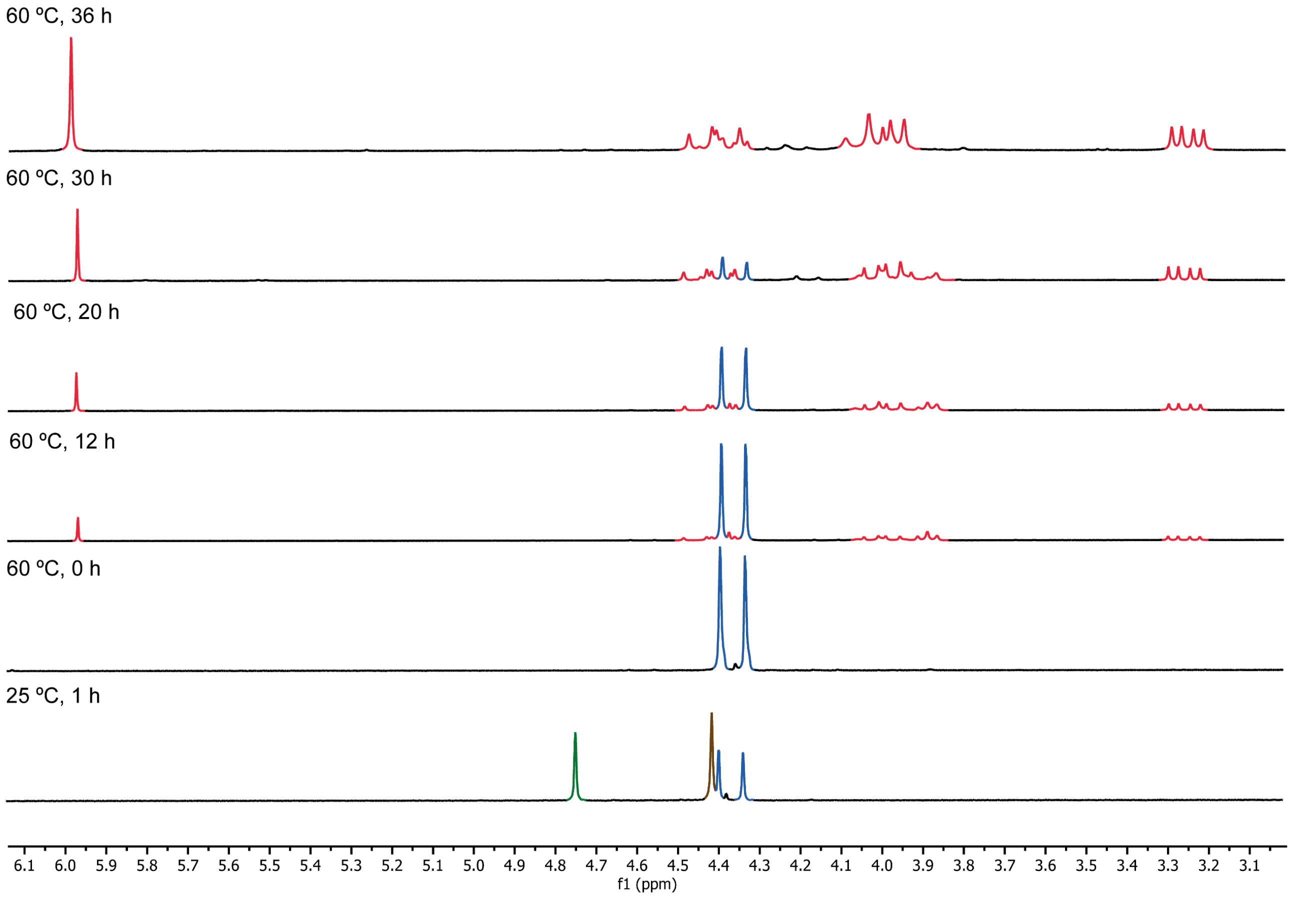

Figure S2. Selected region of the ^{1}H NMR spectra (300 MHz) showing the evolution of the reaction of 5-azidomethyltriazole **2b** with PPh_3 (**5a**) (1 equiv) in $CDCl_3$ at 60 ºC, and the formation of **8ba** and **9ba**. Colours: PN_3 adduct in green; iminophosphorane **8ba** in blue; 5-azidomethyltriazole **2b** in brown; diazaphosphetidine **9ba** in red.

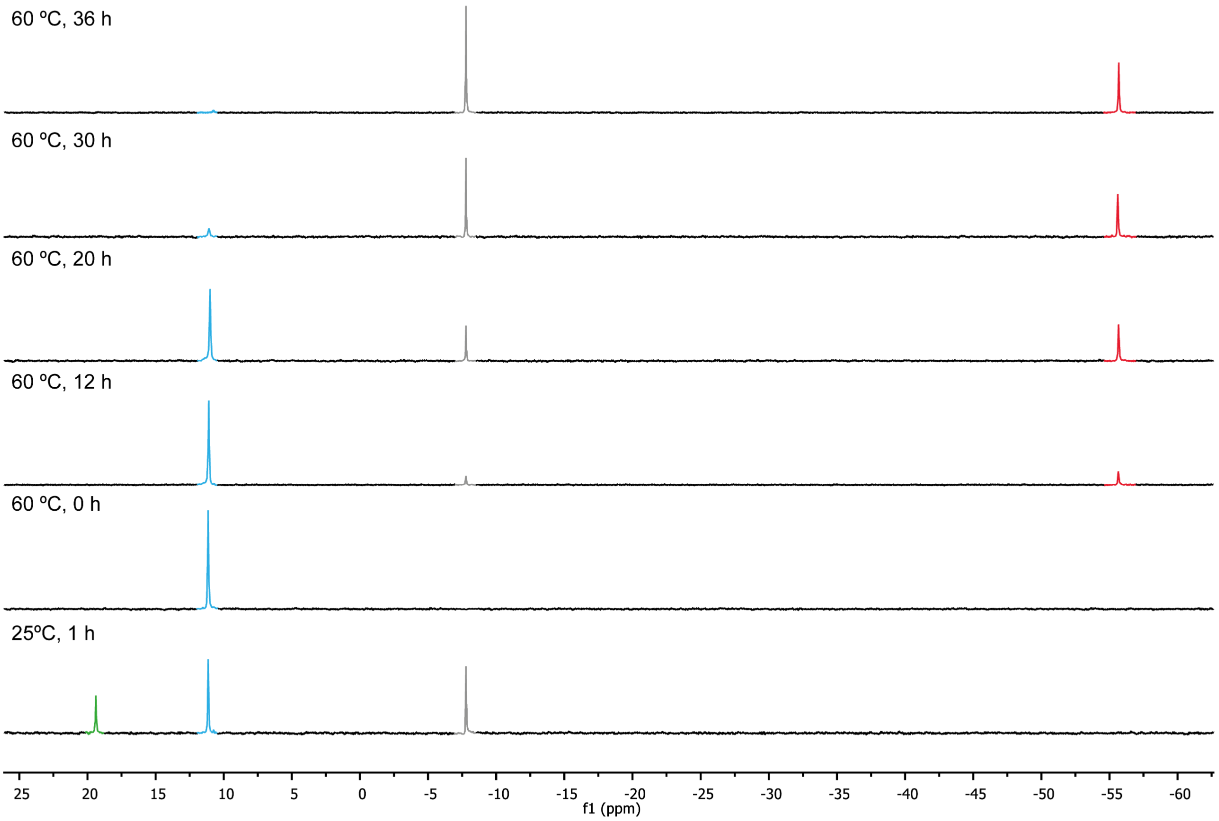

Figure S3. $^{31}P\{^1H\}$ NMR spectra (121.5 MHz) showing the evolution of the reaction of 5-azidomethyltriazole **2b** with PPh_3 (**5a**) (1 equiv) in $CDCl_3$ at 60 °C and the formation of **8ba** and **9ba**. Colours: PN_3 adduct in green; iminophosphorane **8ba** in blue; triphenylphosphine (**5a**) in grey; diazaphosphetidine **9ba** in red.

9. Other unsuccessful reactions between 5-azidomethyltriazoles 2 and phosphines 5.

Table S1. Other reactions between 5-azidomethyltriazoles **2** and phosphines **5** in which the corresponding diazaphosphetidine was detected but not isolated

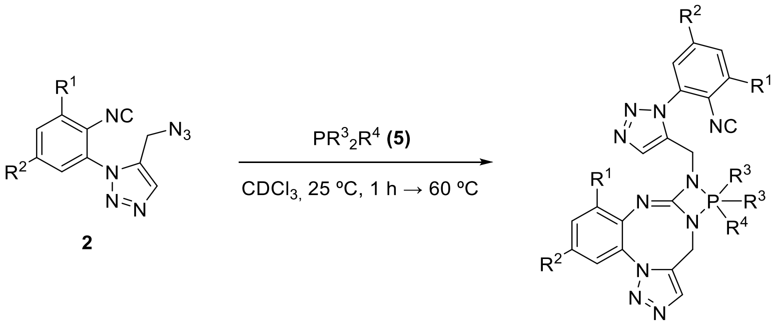

R^1	R^2	R^3	R^4	$^{31}P\{^1H\}$ NMR; δ (ppm) of the non-isolated diazaphosphetidine
CH_3	CH_3	4-$H_3COC_6H_4$	4-$H_3COC_6H_4$	- 54.50
CH_3	CH_3	4-$H_3CC_6H_4$	4-$H_3CC_6H_4$	- 55.64
CH_3	CH_3	4-ClC_6H_4	4-ClC_6H_4	- 60.30
CH_3	CH_3	C_6H_5	CH_3	- 57.14
CH_3	H	C_6H_5	4-$H_3CC_6H_4$	- 52.46
Cl	CH_3	4-$H_3COC_6H_4$	4-$H_3COC_6H_4$	- 48.59
Cl	CH_3	4-$H_3CC_6H_4$	4-$H_3CC_6H_4$	- 49.62
Cl	CH_3	C_6H_5	CH_3	- 51.12

10. X-Ray crystal data and structure refinement of 9ba and 14b.

10.1 Crystal data and structure refinement for 9ba.

Single crystals of $C_{42}H_{37}N_{10}P$ [Galo_452_2_0m] were obtained by slow diffusion of diethyl ether in a solution of $CHCl_3$. Intensities were registered at low temperature (100.0 K) on a Bruker D8 QUESTPHOTON II CMOS system equipped with a multilayer monochromator and a Mo K/a Incoatec microfocus sealed tube (λ = 0.71073 Å). Absorption corrections were based on multi-scans (program SADABS). Using Olex2,[3] the structure was solved with the SHELXT[4] structure solution program using Intrinsic Phasing and refined with the SHELXL[5] refinement package using Least Squares minimisation. Hydrogen atoms were included using a riding model. A solvent mask was calculated, and 57 electrons were found in a volume of 162 $Å^3$ in 1 void per unit cell. This is consistent with the presence of 1 molecule of $CHCl_3$ per unit cell, which account for 58 electrons per unit cell. The structure was deposited with CSD (deposition number CCDC 2387940).

Table S2. Crystal data and structure refinement for **9ba**

	**9ab**
Empirical formula	$C_{42}H_{37}N_{10}P$
Formula weight	712.78
T [K]	100(2)
Wavelength [Å]	0.71073
Crystal system	Triclinic
Space group	P-1
a (Å)	9.4834(9)
b (Å)	12.7331(12)
c (Å)	16.3676(15)
α (°)	82.869(4)
β (°)	86.165(4)
γ (°)	75.588(4)
V [$Å^3$]	1898.1(3)
Z	2
ρ [$g\cdot cm^{-3}$]	1.247
μ [mm^{-1}]	0.117
F_{000}	748
Crystal size [mm^3]	0.17 x 0.07 x 0.03
θ range (º)	3.324-61.17
h	-13 to 13
k	-18 to 18
l	-23 to 23
Reflections collected	155568

Independent reflections	11668
R(int)	0.0738
Refinement method	
Parameters	482
Restraints	0
Goodness-of-fit on F^2	1.090
$R1$ [I > 2σ(I)]	0.0536
$wR2$ [I > 2σ(I)]	0.0341
$R1$ (all data)	0.0687
$wR2$ (all data)	0.1240
$\Delta\rho$ [e·Å^{-3}]	0.44/-0.47

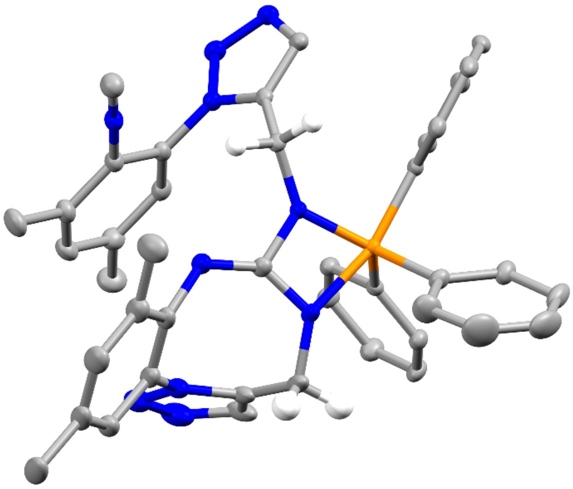

Figure S4. Molecular structure of **9ab** with thermal ellipsoids drawn at 50% probability. For clarity, selected hydrogens atoms have been deleted.

Comments on CHECKCIF:

Alert level B

PLAT315_ALERT_2_B Singly Bonded Carbon Detected (H-atoms Missing). C24 Check

Author Response: C24 is forming part of an isocyanide group, having a negative charge on and a positive charge on the contiguous N10. No H atom is missed on C24.

10.2 Crystal data and structure refinement for 14b.

Single crystals of $C_{24}H_{22}N_{10}$ [I_GCF_75_2_0msp1_a] were obtained by slow diffusion of Et_2O in a solution of $CHCl_3$. Intensities were registered at low temperature (100.0 K) on a Bruker D8

QUESTPHOTON II CMOS system equipped with a multilayer monochromator and a Mo K/a Incoatec microfocus sealed tube (λ = 0.71073 Å). Absorption corrections were based on multi-scans (program SADABS). Using Olex2,[3] the structure was solved with the SHELXT[4] structure solution program using Intrinsic Phasing and refined with the SHELXL[5] refinement package using Least Squares minimisation. Hydrogen atoms were included using a riding model. The structure was deposited with CSD (deposition number CCDC 2387939).

Table S3. Crystal data and structure refinement for **14b**

	**14b**
Empirical formula	$C_{24}H_{22}N_{10}$
Formula weight	450.51
T [K]	100(2)
Wavelength [Å]	0.71073
Crystal system	Monoclinic
Space group	$P2_1/c$
a (Å)	9.7219(5)
b (Å)	9.0498(5)
c (Å)	12.8029(7)
α (°)	90
β (°)	95.018(2)
γ (°)	90
V [Å^3]	1122.10(10)
Z	2
ρ [g·cm^{-3}]	1.333
μ [mm^{-1}]	0.087
F_{000}	472.0
Crystal size [mm^3]	0.15 x 0.11 x 0.08
θ range (°)	5.52 - 61.026
h	-13 to 13
k	-12 to 12
l	-18 to 18
Reflections collected	26170
Independent reflections	3416
R(int)	0.0389
Parameters	178
Restraints	0
Goodness-of-fit on F^2	1.044
*R*1 [I > 2σ(I)]	0.0439

*wR*2 [I > 2σ(I)]	0.1159
*R*1 (all data)	0.05
*wR*2 (all data)	0.1209
$\Delta\rho$ [e·Å^{-3}]	0.41/-0.20

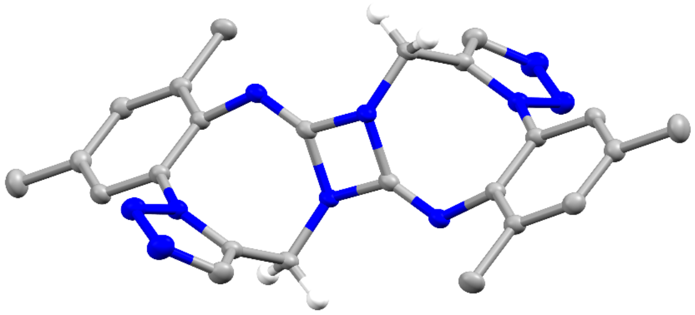

Figure S5. Molecular structure of **14b** (tilted view) with thermal ellipsoids drawn at 50% probability. For clarity, selected hydrogens atoms have been deleted.

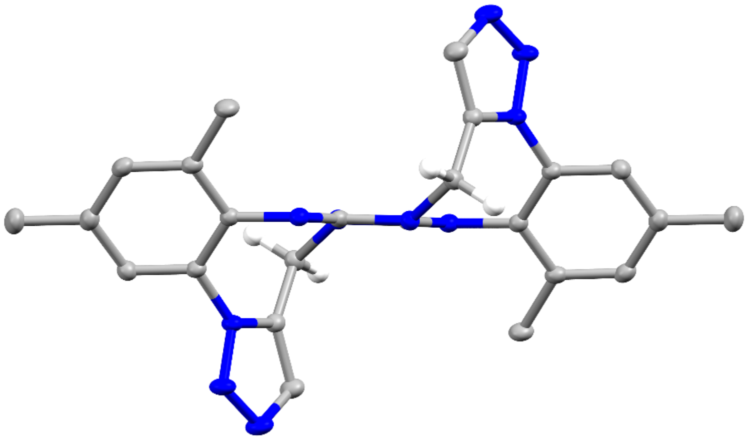

Figure S6. Molecular structure of **14b** (parallel view to the 1,3-diazetidine plane) with thermal ellipsoids drawn at 50% probability. For clarity, selected hydrogens atoms have been deleted.

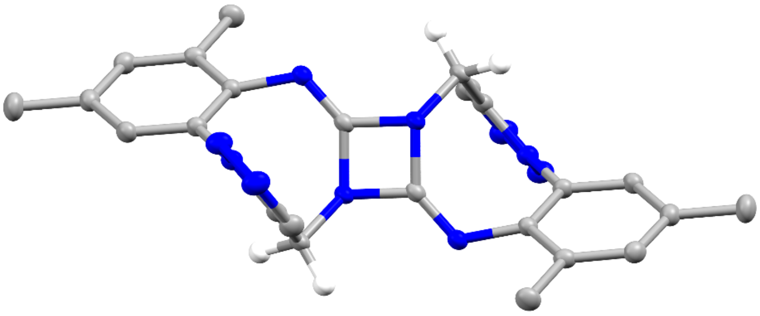

Figure S7. Molecular structure of **14b** (perpendicular view to the 1,3-diazetidine plane) with thermal ellipsoids drawn at 50% probability. For clarity, selected hydrogens atoms have been deleted.

11. Computational details.

11.1 Methods

Geometries of the molecules were optimized by using the wB97X-D[6] hybrid-functional with the Def2-SVP basis set.[7] The nature of minimum and transition structures of all stationary points on the potential energy surface was confirmed by frequency analysis at the same level of theory. The stability of the resulting wave functions was checked for all the optimized structures.[8] Solvent effects were calculated with the PCM solvation model with *chloroform* parameters.[9] The ultrafine grid implemented in Gaussian 16 C. 01 was used.[10] The computed thermochemical corrections at PCM($CHCl_3$)/wB97XD/Def2-SVP level were combined with single point energies at the PCM($CHCl_3$)/wB97xD/Def2-TZVPD//PCM($CHCl_3$)/wB97XD/Def2-SVP level to yield Gibbs free energies G at 298.15 K ($G_{298,sol}$). Computed ^{31}P NMR chemical shifts for the geometry optimized structures were computed at GIAO-PCM($CHCl_3$)/mpw1k/6-311++g(2d,2p) theoretical level.

11.2 Data

Table S4. Imaginary frequencies (cm^{-1}), Electronic ($E_{SCF,298.sol}$), Gibbs free ($G_{298,sol}$), and enthalpy ($H_{298,sol}$) energies (in Hartree) for the stationary points shown in Figure 1 computed at PCM($CHCl_3$)/wB97xD/Def2-TZVPD//PCM($CHCl_3$)/wB97XD/Def2-SVP level.

		wB97XD/Def2-SVP			wB97xD/Def2-TZVPD//wB97XD/Def2-SVP	
Filename	ImFreqs	$E_{tot,sol}$	$G_{298,sol}$	$H_{298,sol}$	$E_{tot,sol}$	$G_{298,sol}$
**PPh_3**	-	-1035.4699765	-1035.2393000	-1035.1767020	-1036.3152395	-1036.0845630
**8ca**		-1733.0097662	-1732.6020690	-1732.5092540	-1734.6165132	-1734.2088160
**TS1**	-370.70	-1732.9692367	-1732.5614770	-1732.4702740	-1734.5729458	-1734.1651861
**10ca**		-1732.9926831	-1732.5820890	-1732.4914150	-1734.5948273	-1734.1842332
**TS2**	-47.14	-1732.9918037	-1732.5792540	-1732.4913970	-1734.5928019	-1734.1802522
**11ca**	-	-1733.0340332	-1732.6242290	-1732.5324990	-1734.6285400	-1734.2187358
**TS3**	-190.4	-1733.0041004	-1732.5977300	-1732.5045570	-1734.5987995	-1734.1924291
**12c**	-	-697.5191567	-697.3649270	-697.3135500	-698.2716367	-698.1174070
**TS4**	-93.46	-2430.5511878	-2429.9668860	-2429.8439070	-2432.9039619	-2432.3196601
**9ca**	-	-2430.6246878	-2430.0302560	-2429.9138210	-2432.9672470	-2432.3728152
**9ca'**	-	-2430.6102102	-2430.0156270	-2429.8993210	-2432.9543954	-2432.3598122

Cartesian Coordinates

PPh$_3$

SCF = -1036.31523948

P	0.000061	-0.001186	-1.262284
C	1.437027	-0.812841	-0.440959
C	2.703204	-0.580315	-0.997812
C	1.338128	-1.634074	0.689079
C	3.845922	-1.137155	-0.426898
H	2.796374	0.047759	-1.888684
C	2.480884	-2.201992	1.253564
H	0.361567	-1.831786	1.136729
C	3.736313	-1.952187	0.700548
H	4.825115	-0.941280	-0.869479
H	2.388031	-2.841606	2.134226
H	4.629759	-2.396214	1.145108
C	-1.422233	-0.838604	-0.441182
C	-2.086011	-0.338601	0.685993
C	-1.853319	-2.052939	-0.995220
C	-3.150817	-1.041889	1.250436
H	-1.769004	0.607318	1.130870
C	-2.908672	-2.761661	-0.424401
H	-1.354354	-2.450703	-1.883837
C	-3.561809	-2.255391	0.700132
H	-3.659881	-0.638699	2.128834
H	-3.228304	-3.708745	-0.864806
H	-4.394404	-2.805247	1.144528
C	-0.014713	1.649454	-0.441578
C	-0.848967	2.630278	-0.997727
C	0.747203	1.973817	0.687771
C	-0.936534	3.898409	-0.426733
H	-1.440486	2.397335	-1.888027
C	0.669043	3.247390	1.252484
H	1.406608	1.226009	1.134072
C	-0.174760	4.210340	0.700148
H	-1.595628	4.648987	-0.868662
H	1.270254	3.486316	2.132681
H	-0.235896	5.206112	1.144830

8ca

SCF = -1734.61651320

C	2.880642	0.999519	1.853481
C	2.490762	-0.265941	1.426925
C	2.539025	-0.576953	0.073910
C	3.012753	0.369290	-0.845008
N	3.107285	0.041757	-2.183058
C	3.211568	-0.205279	-3.325537
C	3.394772	1.655939	-0.430942
C	3.316441	1.948755	0.933057
H	3.604958	2.944744	1.275521
N	2.135006	-1.871637	-0.347384
N	2.722190	-2.939967	0.230949
N	2.159715	-3.998690	-0.235683
C	1.191972	-3.639051	-1.114904
C	1.148155	-2.263425	-1.200971
C	0.229159	-1.364279	-1.988875
N	-0.065212	-0.097256	-1.386648
P	-1.136311	0.168452	-0.261979
C	-0.847275	-0.587586	1.383714
C	-0.760283	-1.985337	1.456465
C	-0.406226	-2.610808	2.648435
C	-0.155168	-1.844813	3.788823
C	-0.260959	-0.456182	3.730880
C	-0.597551	0.171996	2.531336
H	-0.649123	1.261757	2.491925
H	-0.068005	0.145824	4.621285
H	0.122947	-2.334094	4.724652
H	-0.323249	-3.698617	2.686158
H	-0.960585	-2.597048	0.573414
C	-1.164935	1.958814	0.018143
C	-2.239346	2.582023	0.662204
C	-2.203066	3.954578	0.901833
C	-1.096978	4.705026	0.499345
C	-0.029285	4.084720	-0.150131
C	-0.063171	2.713287	-0.394069
H	0.753065	2.205441	-0.910221
H	0.834081	4.671505	-0.471637
H	-1.071102	5.780440	0.688571
H	-3.043039	4.440908	1.401915
H	-3.109232	1.999569	0.976923
C	-2.838901	-0.336918	-0.693963
C	-3.199234	-0.251423	-2.045200
C	-4.485173	-0.601404	-2.450530

C	-5.418472	-1.036977	-1.508602
C	-5.064774	-1.125189	-0.162200
C	-3.776791	-0.779617	0.246026
H	-3.502596	-0.859395	1.300732
H	-5.793722	-1.468990	0.574554
H	-6.426550	-1.312031	-1.826443
H	-4.760325	-0.535335	-3.505183
H	-2.461108	0.090435	-2.775456
H	0.709979	-1.185646	-2.967454
H	-0.665902	-1.979099	-2.216933
H	0.568617	-4.369581	-1.625467
H	2.124758	-1.016479	2.127754
H	2.829268	1.250169	2.914360
C	3.857165	2.670657	-1.436814
H	4.120346	3.616054	-0.945920
H	4.737918	2.310163	-1.989839
H	3.071422	2.872531	-2.181754

TS1

SCF = -1734.57294577

C	-5.924376	0.452810	-0.932060
C	-5.063080	-0.605981	-0.681306
C	-3.813561	-0.360850	-0.110990
C	-3.407278	0.939705	0.244975
C	-4.280604	2.019129	-0.045585
C	-5.516482	1.754764	-0.630452
H	-6.904533	0.269846	-1.375445
H	-6.186103	2.592044	-0.843312
N	-2.908044	-1.448987	0.030941
C	-1.691190	-1.572291	-0.562196
C	-1.317863	-2.863616	-0.257270
H	-0.406586	-3.393354	-0.526344
N	-3.259581	-2.585357	0.646296
N	-2.301834	-3.436904	0.480076
C	-0.997856	-0.450294	-1.272829
H	-1.744786	0.167324	-1.795541
H	-0.359530	-0.887177	-2.055858
N	-0.245244	0.397493	-0.362063
H	-5.332984	-1.632511	-0.933532
N	-2.262832	1.208155	0.952083
C	-1.029728	1.181362	1.064060
P	1.339668	0.221875	-0.061721

C	2.231037	1.798508	-0.185029
C	3.571539	1.831791	-0.593960
C	1.577073	2.987490	0.159050
C	4.250464	3.047042	-0.656453
H	4.090206	0.911854	-0.873876
C	2.262830	4.198589	0.094241
H	0.538162	2.950112	0.495166
C	3.596495	4.230166	-0.313326
H	5.293378	3.068332	-0.978499
H	1.750124	5.123772	0.364630
H	4.129242	5.182087	-0.365395
C	1.708011	-0.480382	1.573490
C	0.749590	-1.278829	2.206698
C	2.962487	-0.294399	2.165538
C	1.050080	-1.897228	3.417895
H	-0.236235	-1.410262	1.758470
C	3.257005	-0.912342	3.379400
H	3.713625	0.339367	1.689195
C	2.302966	-1.715638	4.003952
H	0.297094	-2.517327	3.907796
H	4.234893	-0.760901	3.840534
H	2.534743	-2.197244	4.956310
C	2.091871	-0.894069	-1.276002
C	2.457546	-2.199500	-0.930363
C	2.218299	-0.463716	-2.605471
C	2.947958	-3.066519	-1.906687
H	2.362787	-2.543401	0.101841
C	2.709632	-1.332604	-3.574903
H	1.933261	0.554295	-2.883245
C	3.073830	-2.634654	-3.225691
H	3.234040	-4.083440	-1.631874
H	2.808315	-0.993002	-4.607599
H	3.458703	-3.315323	-3.987824
C	-3.854355	3.412749	0.316235
H	-3.619432	3.475249	1.390227
H	-2.936036	3.699720	-0.220150
H	-4.638681	4.143853	0.079054

10ca

SCF = -1734.59482731

C	-5.725162	0.668616	-0.800985
C	-4.901464	-0.434109	-0.631317

C	-3.579923	-0.256117	-0.215764
C	-3.051134	1.022334	0.047904
C	-3.900397	2.141325	-0.140852
C	-5.213678	1.945860	-0.563114
H	-6.760111	0.537503	-1.121172
H	-5.857427	2.818356	-0.701432
N	-2.771458	-1.428645	-0.091019
C	-1.721252	-1.798526	-0.870837
C	-1.414253	-3.065191	-0.427415
H	-0.624809	-3.734605	-0.760697
N	-3.097467	-2.412166	0.760079
N	-2.281759	-3.394400	0.562677
C	-1.050002	-0.853973	-1.810293
H	-1.775529	-0.359766	-2.473841
H	-0.328711	-1.396113	-2.436342
N	-0.320514	0.145927	-1.039879
H	-5.262761	-1.446971	-0.816113
N	-1.784173	1.269434	0.583915
C	-0.679414	0.900545	0.070626
P	1.095190	0.299381	-0.059175
C	2.094425	1.835559	-0.078045
C	3.460490	1.830577	-0.392472
C	1.485397	3.036710	0.306695
C	4.201984	3.008637	-0.322733
H	3.954045	0.904865	-0.693175
C	2.228003	4.214051	0.369549
H	0.423363	3.046602	0.560654
C	3.586678	4.201767	0.055006
H	5.265849	2.993018	-0.567993
H	1.741879	5.145058	0.667989
H	4.168294	5.124856	0.104444
C	1.285206	-0.499327	1.582637
C	0.207547	-1.164905	2.175613
C	2.521026	-0.469056	2.239578
C	0.367658	-1.806556	3.402607
H	-0.770981	-1.182093	1.693537
C	2.674326	-1.096772	3.474255
H	3.373177	0.045605	1.789109
C	1.599742	-1.772040	4.054079
H	-0.479451	-2.331081	3.848725
H	3.639328	-1.060022	3.983971
H	1.722641	-2.269124	5.018697
C	2.153359	-0.852689	-1.101284

C	2.558211	-2.121216	-0.674753
C	2.391646	-0.487175	-2.435995
C	3.202790	-2.996764	-1.553103
H	2.368990	-2.442934	0.351325
C	3.038852	-1.354432	-3.311737
H	2.067077	0.493560	-2.797378
C	3.447701	-2.614931	-2.869589
H	3.512733	-3.983363	-1.201572
H	3.222562	-1.048978	-4.344206
H	3.952935	-3.298941	-3.554989
C	-3.362182	3.517153	0.134949
H	-2.966452	3.577302	1.160043
H	-2.523626	3.758000	-0.537046
H	-4.140348	4.281354	0.005400

TS2

SCF = -1734.59280188

C	-5.747904	0.576128	-0.630152
C	-4.896313	-0.514060	-0.522564
C	-3.552688	-0.310842	-0.204549
C	-3.027724	0.975915	0.021146
C	-3.904220	2.081357	-0.104281
C	-5.241659	1.862046	-0.429388
H	-6.801349	0.428519	-0.873732
H	-5.908891	2.723160	-0.519690
N	-2.701889	-1.455711	-0.134527
C	-1.678426	-1.775440	-0.969053
C	-1.271931	-3.014845	-0.525261
H	-0.462841	-3.641631	-0.893282
N	-2.920957	-2.440008	0.748095
N	-2.060890	-3.376885	0.517331
C	-1.114776	-0.812605	-1.963175
H	-1.912328	-0.325890	-2.546001
H	-0.462238	-1.348889	-2.664588
N	-0.316118	0.186075	-1.264217
H	-5.252775	-1.533023	-0.681360
N	-1.730512	1.223453	0.472436
C	-0.658485	0.886525	-0.129205
P	1.072691	0.310466	-0.097318
C	2.123683	1.763017	-0.413856
C	3.471614	1.638203	-0.772925
C	1.574872	3.038252	-0.227269

C	4.257795	2.776538	-0.945678
H	3.915341	0.651554	-0.917122
C	2.363314	4.172984	-0.403316
H	0.526126	3.141428	0.060168
C	3.704921	4.043498	-0.763424
H	5.307738	2.670680	-1.225792
H	1.927055	5.163128	-0.257405
H	4.321900	4.933876	-0.902255
C	1.281948	-0.132196	1.684702
C	0.252097	-0.823278	2.335428
C	2.452290	0.177728	2.386085
C	0.396230	-1.206137	3.667821
H	-0.674101	-1.069385	1.810368
C	2.588266	-0.195050	3.722990
H	3.265320	0.715073	1.891862
C	1.563076	-0.890725	4.363765
H	-0.410695	-1.750510	4.162385
H	3.501399	0.058988	4.265576
H	1.672702	-1.185576	5.409636
C	2.018448	-1.109409	-0.860363
C	2.445722	-2.229471	-0.141721
C	2.225182	-1.078042	-2.248781
C	3.068442	-3.295694	-0.795913
H	2.289406	-2.285869	0.936709
C	2.857114	-2.133141	-2.899573
H	1.887687	-0.212639	-2.826742
C	3.277560	-3.249184	-2.171759
H	3.390403	-4.166786	-0.221322
H	3.018627	-2.088812	-3.978804
H	3.766526	-4.082528	-2.680932
C	-3.367721	3.464988	0.132278
H	-2.892826	3.529739	1.122975
H	-2.589218	3.720030	-0.603915
H	-4.165134	4.217524	0.067159

11ca

SCF = -1734.62853998

C	-5.302530	1.649931	-0.441497
C	-4.869638	0.337536	-0.312843
C	-3.502988	0.059177	-0.301846
C	-2.524534	1.068673	-0.418343
N	-1.164710	0.873886	-0.275289

C	-0.350422	0.056039	-0.891856
P	1.304701	0.089973	-0.058473
N	-0.363370	-0.874263	-1.800706
C	-1.651477	-1.371530	-2.246720
C	-2.369053	-2.010089	-1.096071
C	-2.345632	-3.279796	-0.558797
N	-3.048735	-3.273075	0.601212
N	-3.500783	-2.080890	0.818474
N	-3.093817	-1.301707	-0.194236
H	-1.862078	-4.175651	-0.940751
H	-1.489271	-2.131965	-3.022980
H	-2.291422	-0.588404	-2.693555
C	1.284352	-1.322582	1.072658
C	0.064745	-1.732135	1.627801
C	0.030461	-2.826652	2.487072
C	1.208976	-3.502324	2.805168
C	2.424956	-3.088725	2.260506
C	2.466113	-2.002221	1.388856
H	3.417356	-1.693699	0.949031
H	3.346201	-3.620406	2.506071
H	1.178621	-4.362174	3.477686
H	-0.927201	-3.156684	2.893084
H	-0.859398	-1.203893	1.383685
C	2.734577	-0.087644	-1.155807
C	2.691208	-1.001493	-2.218745
C	3.816457	-1.177056	-3.019649
C	4.984264	-0.457776	-2.762121
C	5.029877	0.445051	-1.700662
C	3.908380	0.632645	-0.894838
H	3.953147	1.341310	-0.065509
H	5.942103	1.008631	-1.496047
H	5.864261	-0.601868	-3.392642
H	3.780288	-1.882711	-3.851982
H	1.759949	-1.537864	-2.410049
C	1.514179	1.613170	0.883751
C	1.488531	2.829436	0.188139
C	1.631066	4.025525	0.882201
C	1.796148	4.012584	2.269422
C	1.821101	2.803510	2.961239
C	1.680615	1.599507	2.270434
H	1.695201	0.653641	2.816037
H	1.947895	2.793067	4.045314
H	1.904238	4.953485	2.812964

H	1.606406	4.973170	0.340936
H	1.347449	2.842212	-0.895170
C	-2.986814	2.405472	-0.557649
C	-4.355006	2.668478	-0.569197
H	-4.689924	3.704174	-0.673174
H	-5.575950	-0.488705	-0.217027
H	-6.369199	1.880762	-0.445835
C	-1.975932	3.512497	-0.657614
H	-1.324388	3.522129	0.229724
H	-1.311267	3.367320	-1.523629
H	-2.465506	4.491636	-0.753227

TS3

SCF = -1734.59879951

C	-5.111690	2.040567	-0.500823
C	-5.053401	0.661258	-0.348898
C	-3.838466	0.059148	-0.021669
C	-2.669062	0.821166	0.152245
N	-1.453840	0.217436	0.510873
C	-0.932030	-0.605904	-0.281084
P	1.465923	-0.170701	0.038727
N	-0.932701	-1.443396	-1.194393
C	-2.136717	-2.213774	-1.479800
C	-3.113072	-2.353980	-0.353207
C	-3.545970	-3.478881	0.314264
N	-4.473340	-3.113766	1.232394
N	-4.635721	-1.836117	1.182460
N	-3.825572	-1.347591	0.229408
H	-3.241366	-4.513027	0.170754
H	-1.844693	-3.225980	-1.791412
H	-2.640288	-1.754242	-2.347675
C	1.682207	1.415654	-0.835042
C	1.156616	1.505062	-2.133333
C	1.216886	2.706011	-2.834459
C	1.787488	3.833837	-2.240426
C	2.304968	3.752335	-0.948839
C	2.256591	2.546878	-0.247199
H	2.660828	2.492822	0.766006
H	2.750209	4.632635	-0.480437
H	1.824736	4.779136	-2.786022
H	0.805351	2.766273	-3.844097
H	0.684568	0.629654	-2.588791

C	2.108292	0.118946	1.720202
C	1.208413	0.610553	2.677190
C	1.644258	0.873713	3.974611
C	2.973641	0.638468	4.327252
C	3.868749	0.138156	3.381052
C	3.440233	-0.120778	2.079807
H	4.145689	-0.511831	1.342818
H	4.908279	-0.051720	3.656735
H	3.312827	0.840575	5.345616
H	0.939515	1.256474	4.715777
H	0.163778	0.774229	2.397967
C	2.624940	-1.328736	-0.752150
C	2.381683	-2.696929	-0.568429
C	3.231447	-3.643279	-1.137434
C	4.321663	-3.230237	-1.904080
C	4.561624	-1.869981	-2.100844
C	3.716954	-0.920213	-1.528024
H	3.908441	0.143944	-1.685321
H	5.412684	-1.545315	-2.703275
H	4.984724	-3.972067	-2.354528
H	3.037250	-4.707486	-0.988492
H	1.517985	-3.021121	0.018030
C	-2.734069	2.222124	0.007090
C	-3.959236	2.806268	-0.318593
H	-4.010463	3.891981	-0.433285
H	-5.940816	0.037815	-0.465113
H	-6.057434	2.522488	-0.754499
C	-1.490925	3.045524	0.195761
H	-0.983785	2.783348	1.136477
H	-0.765383	2.859771	-0.611487
H	-1.725344	4.118342	0.207307

12c

SCF = -698.271636743

C	2.249509	-2.109103	0.428986
C	0.875370	-1.919985	0.399830
C	0.329351	-0.665902	0.102102
C	1.203099	0.425804	-0.118777
C	2.598885	0.229458	-0.097214
C	3.103938	-1.042375	0.163683
H	2.653328	-3.096456	0.658561
H	4.185781	-1.191724	0.179168

N	-1.110294	-0.643625	-0.049628
C	-2.159037	0.156978	0.353646
C	-3.275405	-0.540681	-0.040749
H	-4.318868	-0.253166	0.066570
N	-1.612014	-1.750184	-0.648031
N	-2.892060	-1.692974	-0.637684
C	-2.130303	1.557197	0.889570
H	-1.556282	1.631962	1.827758
H	-3.160934	1.864828	1.098406
N	-1.553910	2.445469	-0.157975
H	0.199242	-2.752199	0.586931
N	0.769491	1.753688	-0.226166
C	-0.375537	2.150268	-0.277708
C	3.508251	1.402780	-0.334997
H	3.319714	1.859945	-1.317832
H	3.339416	2.187033	0.418283
H	4.561383	1.096524	-0.291682

TS4

SCF = -2432.90396193

C	6.965641	-0.609000	-1.474197
C	6.247638	-1.233357	-0.461678
C	4.872952	-1.020026	-0.364819
C	4.206390	-0.181505	-1.277365
C	4.931833	0.443169	-2.307326
C	6.306900	0.216410	-2.386375
H	8.041483	-0.771128	-1.558582
H	6.876391	0.702370	-3.182517
N	4.137459	-1.771882	0.609924
C	3.371897	-1.398020	1.679476
C	2.964053	-2.598280	2.217104
H	2.333828	-2.774332	3.085325
N	4.181838	-3.114758	0.518840
N	3.485173	-3.609629	1.481515
C	3.071664	0.003336	2.118565
H	3.989092	0.488660	2.490251
H	2.378564	-0.052576	2.970598
N	2.509435	0.850502	1.080142
H	6.733916	-1.900743	0.250845
N	2.814393	0.014812	-1.178331
C	2.446856	0.464174	-0.078521
N	0.220439	0.681669	-0.711756

C	-0.183646	-0.275003	-1.705372
P	-0.735905	1.471859	0.273867
C	-0.307993	-1.682529	-1.177152
H	0.599935	-0.296246	-2.480181
H	-1.123632	0.010673	-2.211991
N	-1.471460	-2.362080	-0.960693
C	0.648370	-2.546831	-0.685729
C	-2.789807	-2.040182	-1.371251
N	-1.233661	-3.558846	-0.386802
N	0.036759	-3.662684	-0.215437
H	1.726283	-2.413938	-0.647339
C	-3.047037	-1.745173	-2.707493
C	-3.844868	-2.056277	-0.446110
C	-4.345477	-1.455357	-3.112586
H	-2.226710	-1.764184	-3.425806
N	-3.603688	-2.309881	0.889311
C	-5.166146	-1.792841	-0.849617
C	-5.390417	-1.489483	-2.193618
H	-4.545110	-1.219615	-4.158979
C	-3.490766	-2.530081	2.037367
H	-6.408811	-1.272892	-2.522720
C	-2.344455	1.898925	-0.477242
C	-2.336805	2.814894	-1.539317
C	-3.544362	1.276776	-0.117190
C	-3.513924	3.113348	-2.219347
H	-1.404727	3.302888	-1.834915
C	-4.724703	1.588977	-0.791544
H	-3.565437	0.548783	0.695959
C	-4.711481	2.503758	-1.841773
H	-3.497960	3.829273	-3.043507
H	-5.659641	1.111427	-0.493586
H	-5.637066	2.743634	-2.369285
C	-1.103733	0.641515	1.856152
C	-2.033115	1.129346	2.786567
C	-0.394041	-0.529040	2.144803
C	-2.225841	0.467637	3.996763
H	-2.622679	2.022963	2.566897
C	-0.594944	-1.192616	3.353242
H	0.311749	-0.924253	1.412191
C	-1.502397	-0.690497	4.283196
H	-2.950941	0.853484	4.716030
H	-0.052238	-2.116851	3.559581
H	-1.663049	-1.214225	5.227566

C	0.093851	3.049776	0.650295
C	-0.150543	3.781075	1.816650
C	0.988120	3.550701	-0.303235
C	0.486858	5.003912	2.021759
H	-0.825591	3.400011	2.583943
C	1.621630	4.773441	-0.096645
H	1.195344	2.957110	-1.196170
C	1.370687	5.502798	1.065602
H	0.295024	5.565911	2.938008
H	2.320977	5.154682	-0.843748
H	1.870451	6.459873	1.230114
C	4.211638	1.340226	-3.274564
H	3.357566	0.817632	-3.731131
H	3.803489	2.224876	-2.761104
H	4.883347	1.683293	-4.072541
C	-6.281256	-1.811615	0.155264
H	-7.242595	-1.584164	-0.322017
H	-6.358331	-2.795499	0.642533
H	-6.106490	-1.074241	0.954865

9ca

SCF = -2432.96724704

C	4.686362	-3.321166	1.537512
C	3.994215	-3.257528	0.339312
C	2.942013	-2.349443	0.184540
C	2.555698	-1.474079	1.222159
N	1.654696	-0.434853	1.122579
C	0.425683	-0.389033	0.777240
N	-0.472428	-1.368610	0.322758
C	-0.085746	-2.644515	-0.243381
C	0.943990	-2.524905	-1.317108
C	0.874657	-2.606302	-2.691332
N	2.128097	-2.496295	-3.192686
N	2.962070	-2.352871	-2.217149
N	2.269253	-2.355198	-1.072278
H	0.005990	-2.762685	-3.327062
H	-0.976770	-3.122368	-0.665214
H	0.285076	-3.306867	0.555492
P	-1.877394	-0.366100	0.155659
C	-3.040771	-1.774986	-0.324546
C	-3.170646	-2.832778	0.592308
C	-4.066191	-3.874026	0.374164

C	-4.870675	-3.873567	-0.767800
C	-4.769745	-2.824817	-1.676439
C	-3.862922	-1.783542	-1.455863
H	-3.815965	-0.970359	-2.181361
H	-5.400866	-2.808793	-2.567724
H	-5.578228	-4.687036	-0.942054
H	-4.142800	-4.686377	1.100119
H	-2.559207	-2.842926	1.500006
N	-0.397365	0.676589	0.814457
C	-0.130709	1.875475	1.556992
C	-0.073708	3.116018	0.719776
C	-0.809326	4.280006	0.745892
N	-0.414725	5.069327	-0.284642
N	0.520722	4.473368	-0.942845
N	0.749073	3.292713	-0.345806
C	1.726779	2.399952	-0.859480
C	1.519215	1.789745	-2.089864
C	2.469492	0.898994	-2.574304
C	3.619649	0.626865	-1.834779
C	3.852595	1.225866	-0.597668
C	2.887338	2.129917	-0.123970
N	3.084530	2.748475	1.095797
C	3.277057	3.253510	2.137470
H	4.341386	-0.097068	-2.214695
H	2.308252	0.392810	-3.527549
H	0.606145	2.012903	-2.640067
H	-1.599557	4.570899	1.434236
H	-0.908780	2.040119	2.320393
H	0.823407	1.736143	2.091947
C	-2.931243	0.155357	1.561965
C	-2.460806	0.036327	2.873098
C	-3.284606	0.376926	3.945039
C	-4.571621	0.859930	3.711382
C	-5.043113	0.981409	2.403937
C	-4.233461	0.612677	1.331776
H	-4.624301	0.677639	0.313228
H	-6.051877	1.354568	2.216544
H	-5.211530	1.138804	4.551065
H	-2.914572	0.271447	4.966786
H	-1.447099	-0.325070	3.063096
C	-2.007881	0.692067	-1.323791
C	-1.587205	0.184863	-2.556584
C	-1.590478	0.998107	-3.688798

C	-2.002524	2.326739	-3.592228
C	-2.416315	2.838881	-2.361764
C	-2.423761	2.023660	-1.232262
H	-2.742905	2.438692	-0.274738
H	-2.724212	3.882305	-2.274850
H	-1.994615	2.967967	-4.475753
H	-1.256571	0.593083	-4.646106
H	-1.250769	-0.849354	-2.640931
C	3.268069	-1.566733	2.450295
C	4.308684	-2.480182	2.586924
H	4.846363	-2.528323	3.537328
H	4.251753	-3.905527	-0.499334
H	5.510157	-4.026222	1.659849
C	5.061475	0.901166	0.229280
H	5.720095	0.201950	-0.300832
H	5.635681	1.806803	0.476828
H	4.759765	0.432397	1.179250
C	2.901442	-0.630594	3.567199
H	3.030920	0.416987	3.252288
H	1.842957	-0.739027	3.849587
H	3.520351	-0.812105	4.456247

9ca’

SCF = -2432.95439538

C	-3.593477	-2.425579	-2.874573
C	-3.613751	-2.695436	-1.509383
C	-2.629677	-2.135711	-0.704851
C	-1.636567	-1.294454	-1.227426
N	-0.776403	-0.596531	-0.353276
C	0.328215	-1.034752	0.403034
N	0.695431	-2.178263	0.833079
C	-0.199206	-3.310926	0.679436
C	-1.534054	-3.052472	1.317169
C	-2.010769	-3.205669	2.602122
N	-3.274301	-2.715095	2.660896
N	-3.613604	-2.267108	1.498260
N	-2.573632	-2.454588	0.674794
H	-1.514353	-3.637782	3.467628
H	0.250620	-4.176865	1.184314
H	-0.345137	-3.609128	-0.374681
P	-0.669095	1.110874	0.139230
C	-2.195575	1.607851	-0.848001

C	-3.426734	1.015106	-0.522134
C	-4.571655	1.286186	-1.263322
C	-4.511106	2.167854	-2.344897
C	-3.303620	2.780922	-2.666470
C	-2.155139	2.503465	-1.920097
H	-1.219415	2.997393	-2.190603
H	-3.248470	3.481706	-3.502420
H	-5.408557	2.381479	-2.929742
H	-5.515414	0.806960	-0.994616
H	-3.499266	0.329423	0.326687
N	0.850524	0.184863	0.703376
C	1.842521	0.444372	1.704803
C	3.087203	1.065396	1.141880
C	3.717259	2.256475	1.421412
N	4.764065	2.410788	0.571992
N	4.825470	1.394495	-0.216383
N	3.825263	0.558514	0.114977
C	3.622302	-0.631150	-0.630862
C	3.371829	-0.554849	-1.998258
C	3.134251	-1.720927	-2.716452
C	3.138170	-2.957766	-2.071810
C	3.419148	-3.065713	-0.710260
C	3.686532	-1.880769	-0.002754
N	4.030490	-1.961773	1.331666
C	4.339261	-2.072893	2.458553
H	2.926752	-3.865436	-2.640938
H	2.932804	-1.665196	-3.787526
H	3.370501	0.421926	-2.481256
H	3.455847	3.004308	2.165876
H	1.452630	1.136396	2.469707
H	2.071751	-0.502644	2.220074
C	-1.397241	1.349979	1.810371
C	-1.246039	0.368154	2.796881
C	-1.881510	0.502448	4.030049
C	-2.654979	1.628741	4.301608
C	-2.811122	2.612330	3.325277
C	-2.203003	2.464288	2.081439
H	-2.386161	3.213423	1.308634
H	-3.427751	3.491196	3.523589
H	-3.147387	1.735146	5.270386
H	-1.772195	-0.284622	4.778330
H	-0.648872	-0.526441	2.612964
C	0.414975	2.528211	-0.349226

C	1.342568	2.324420	-1.377913
C	2.238412	3.325171	-1.737563
C	2.232230	4.542732	-1.055762
C	1.317831	4.751043	-0.027288
C	0.405822	3.752179	0.322509
H	-0.290484	3.937114	1.141052
H	1.313233	5.696697	0.518832
H	2.948585	5.322752	-1.320571
H	2.959831	3.148418	-2.538026
H	1.378088	1.360941	-1.887424
C	-1.563125	-1.101398	-2.618150
C	-2.564213	-1.661606	-3.418668
H	-2.528725	-1.497755	-4.498077
H	-4.374053	-3.335874	-1.061275
H	-4.364846	-2.844319	-3.523194
C	3.427179	-4.381952	0.007181
H	3.142906	-5.200751	-0.665490
H	4.422225	-4.602440	0.423333
H	2.721264	-4.352574	0.851246
C	-0.439097	-0.314852	-3.228430
H	-0.597111	0.767359	-3.110892
H	0.518444	-0.569650	-2.751495
H	-0.358279	-0.522084	-4.303587

12. NMR spectra of all new synthesized compounds

6aa (^{1}H NMR, 400 MHz, $CDCl_3$, 298 K)

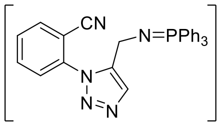

6aa

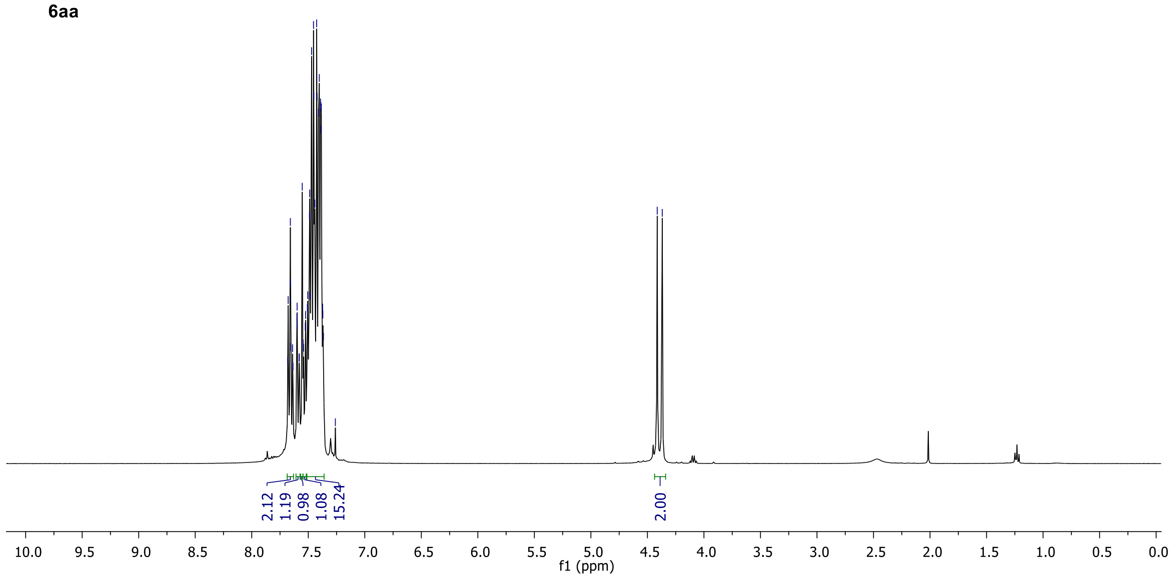

6aa (^{13}C NMR, 100 MHz, $CDCl_3$, 298 K)

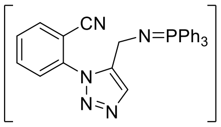

6aa

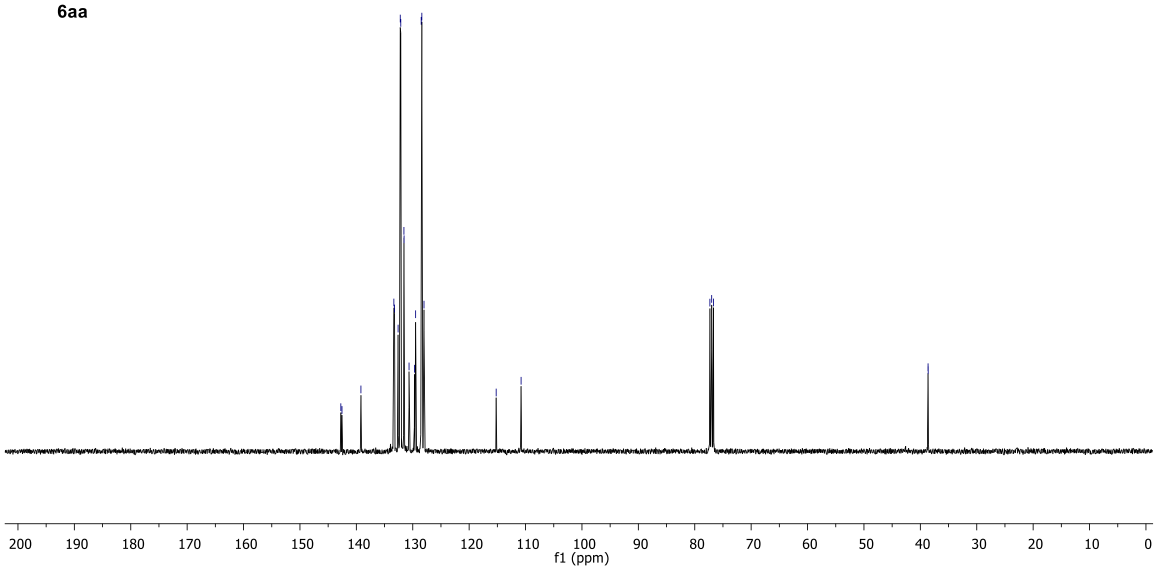

6aa (DEPT-135 NMR, 100 MHz, $CDCl_3$, 298 K)

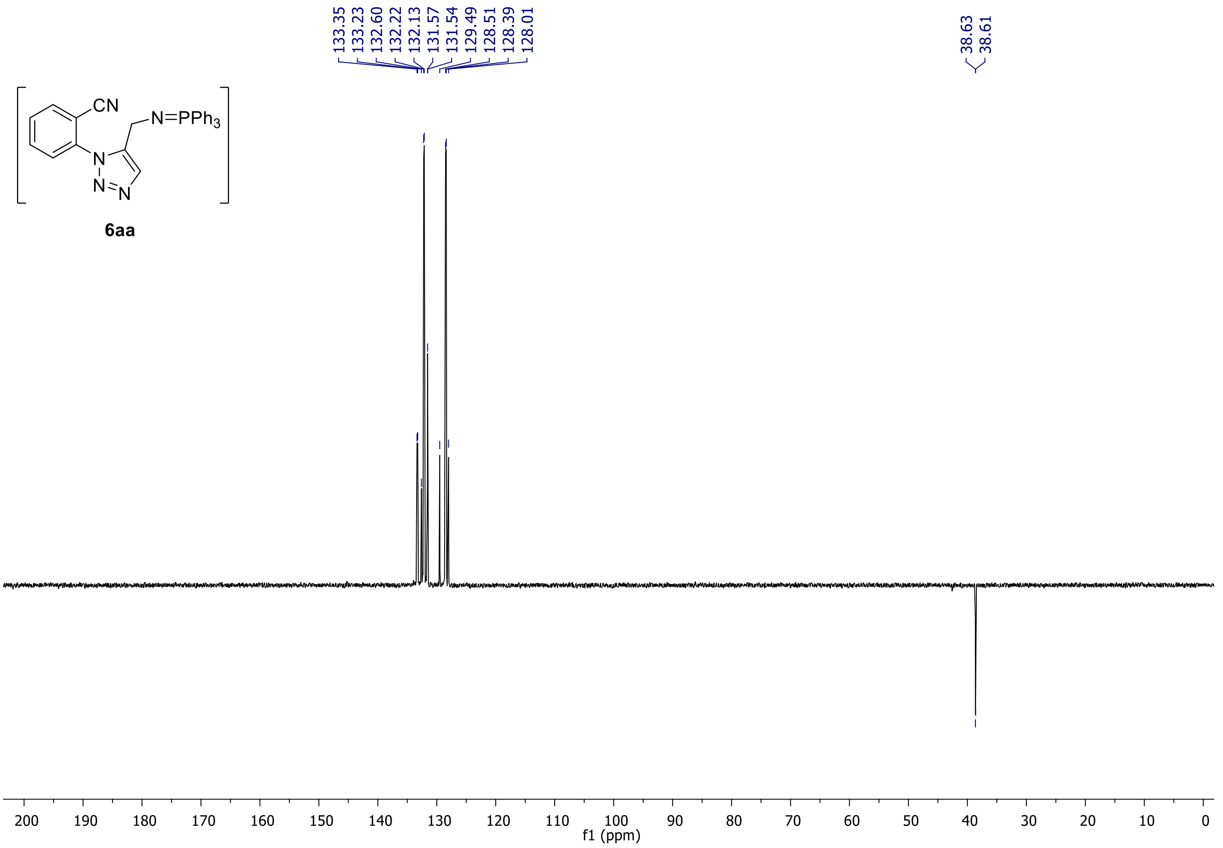

6aa (^{31}P NMR, 162 MHz, $CDCl_3$, 298 K)

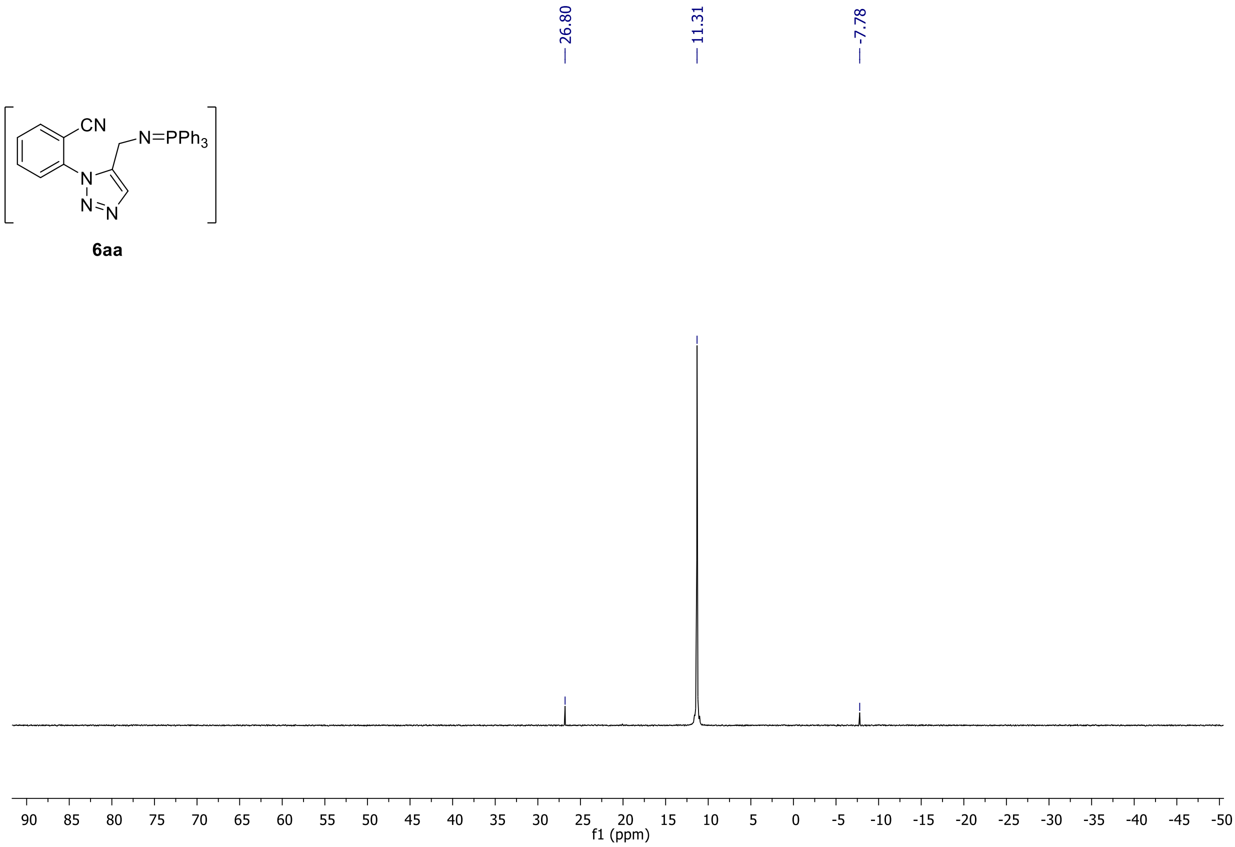

7aa (^{1}H NMR, 400 MHz, $CDCl_3$, 298 K)

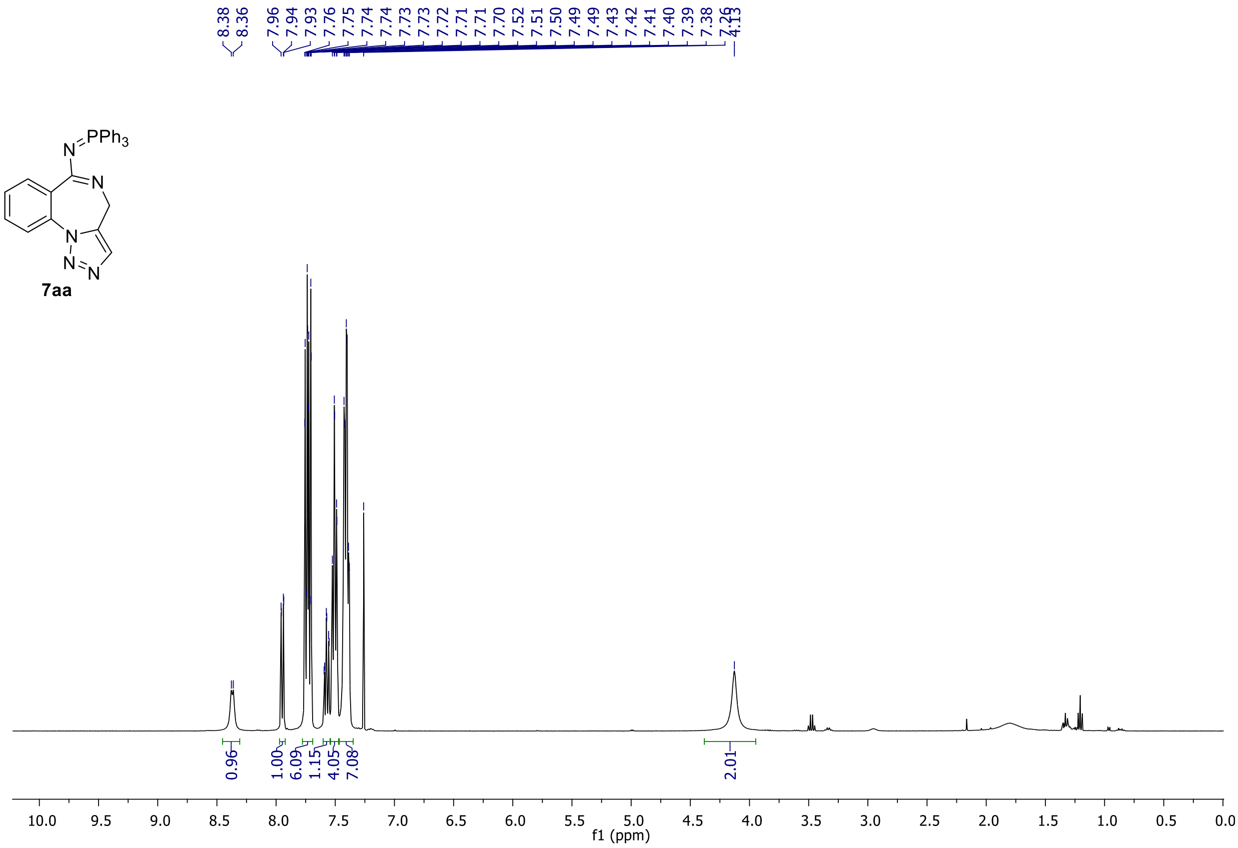

7aa (^{13}C NMR, 100 MHz, $CDCl_3$, 298 K)

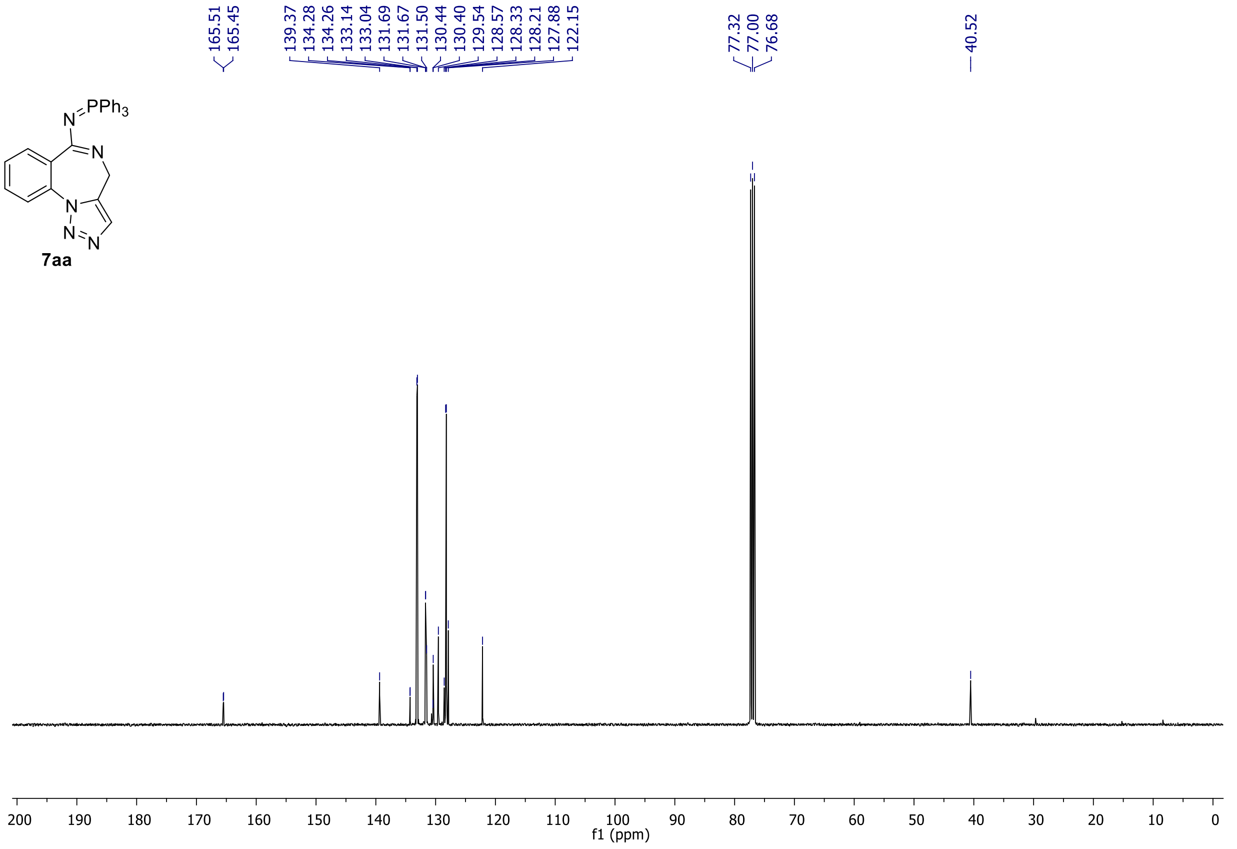

7aa (DEPT-135 NMR, 100 MHz, $CDCl_3$, 298 K)

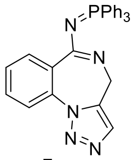

7aa

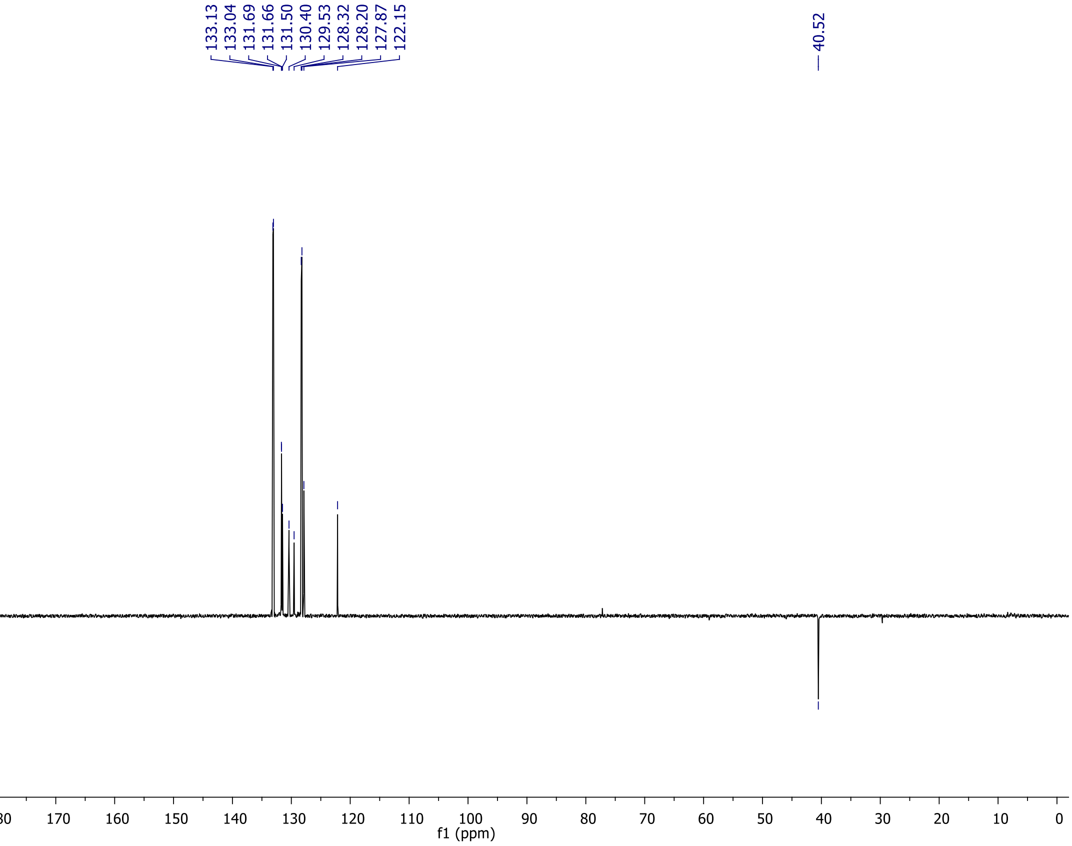

7aa (^{31}P NMR, 162 MHz, $CDCl_3$, 298 K)

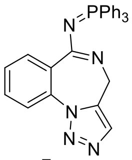

7aa

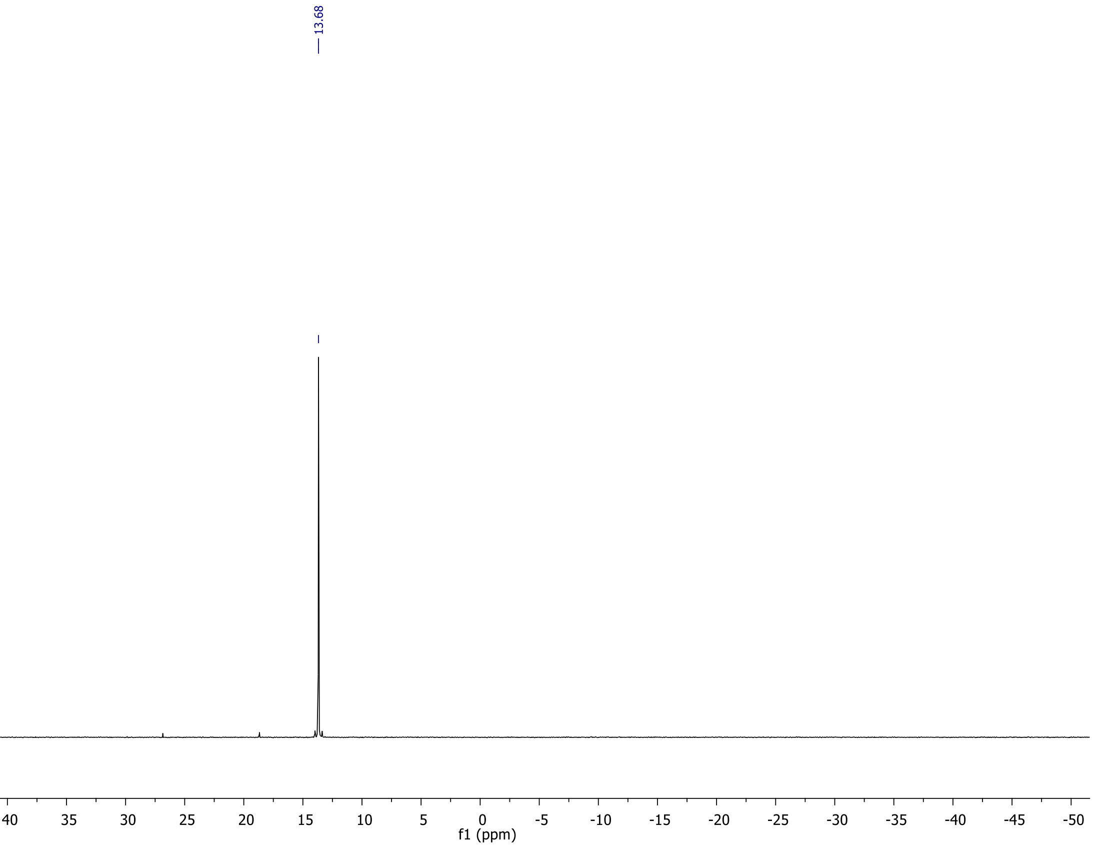

9ba (^{1}H NMR, 300 MHz, $CDCl_3$, 298 K)

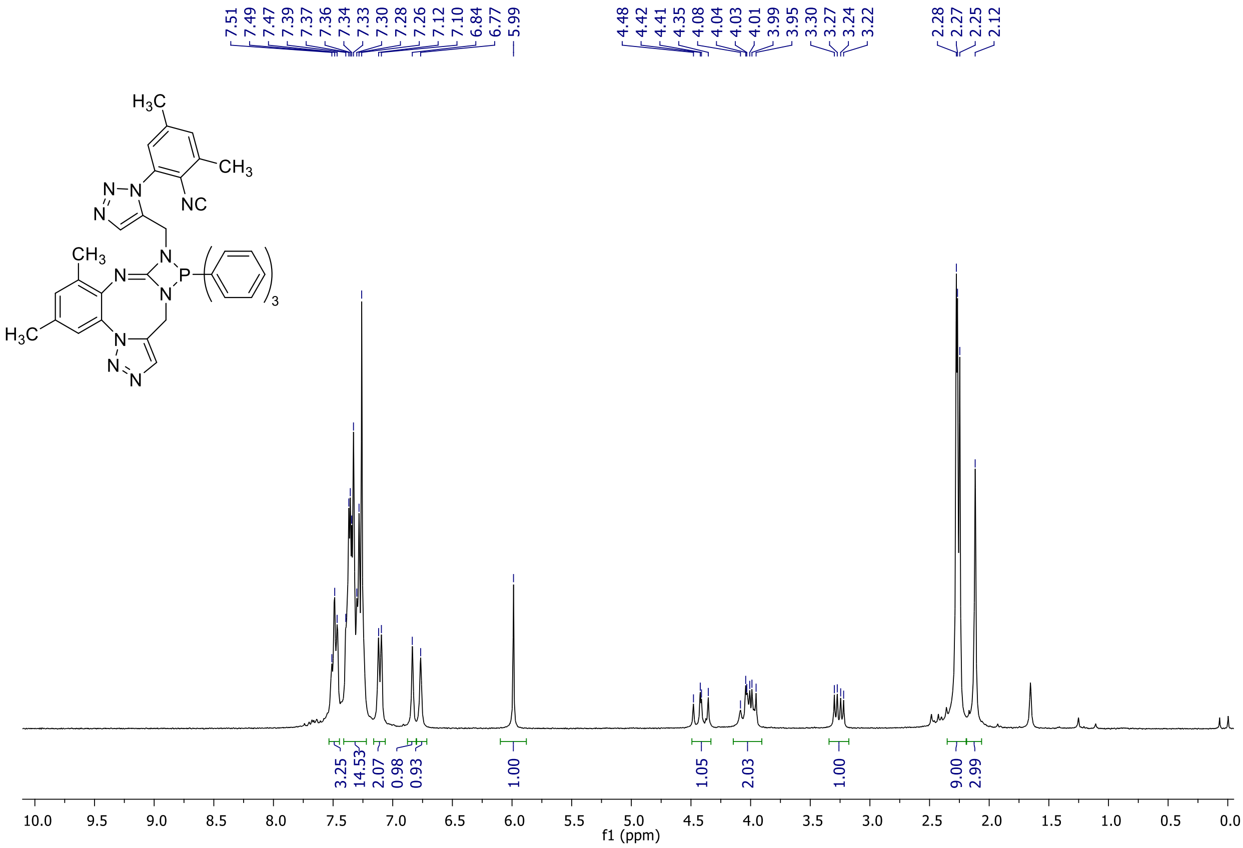

9ba (^{1}H{^{31}P} NMR, 300 MHz, $CDCl_3$, 298 K)

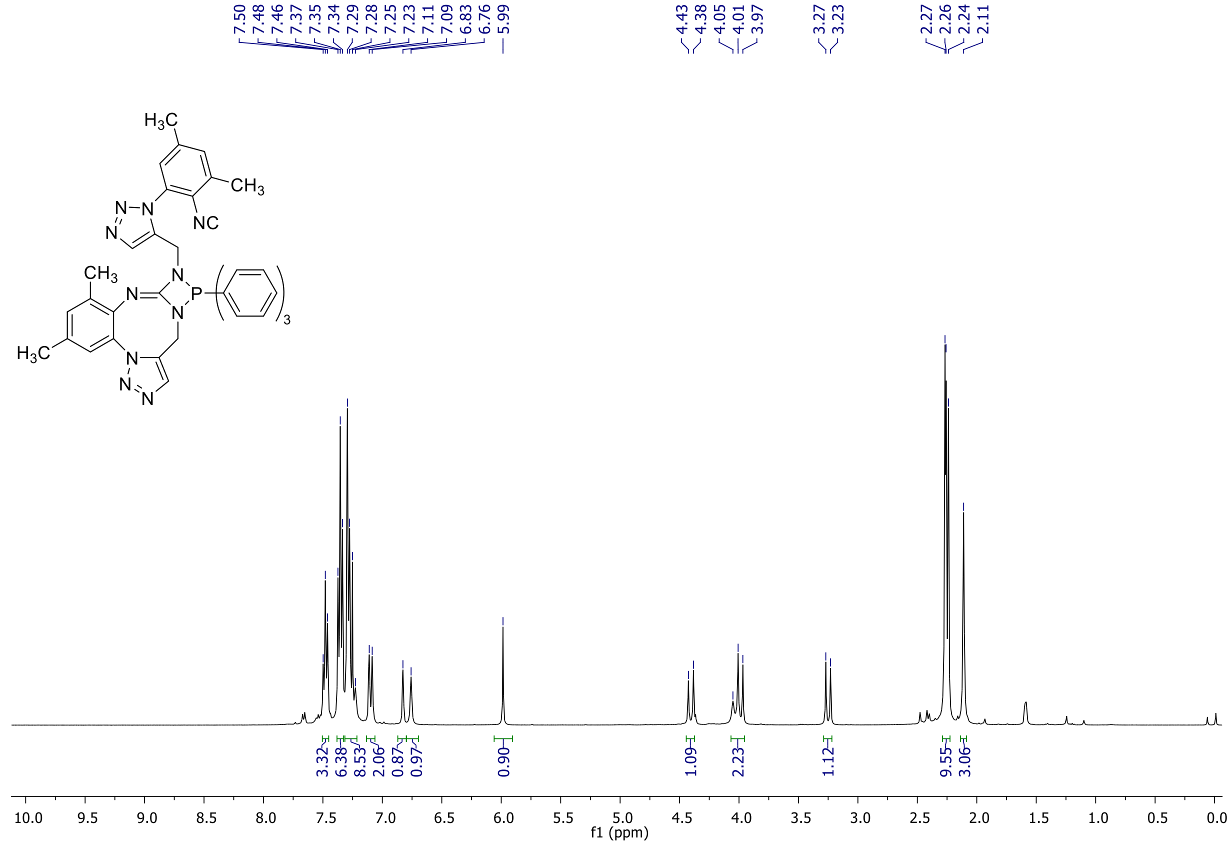

9ba ($^{1}H^{1}H$-COSY NMR, 600 MHz, $CDCl_3$, 298 K)

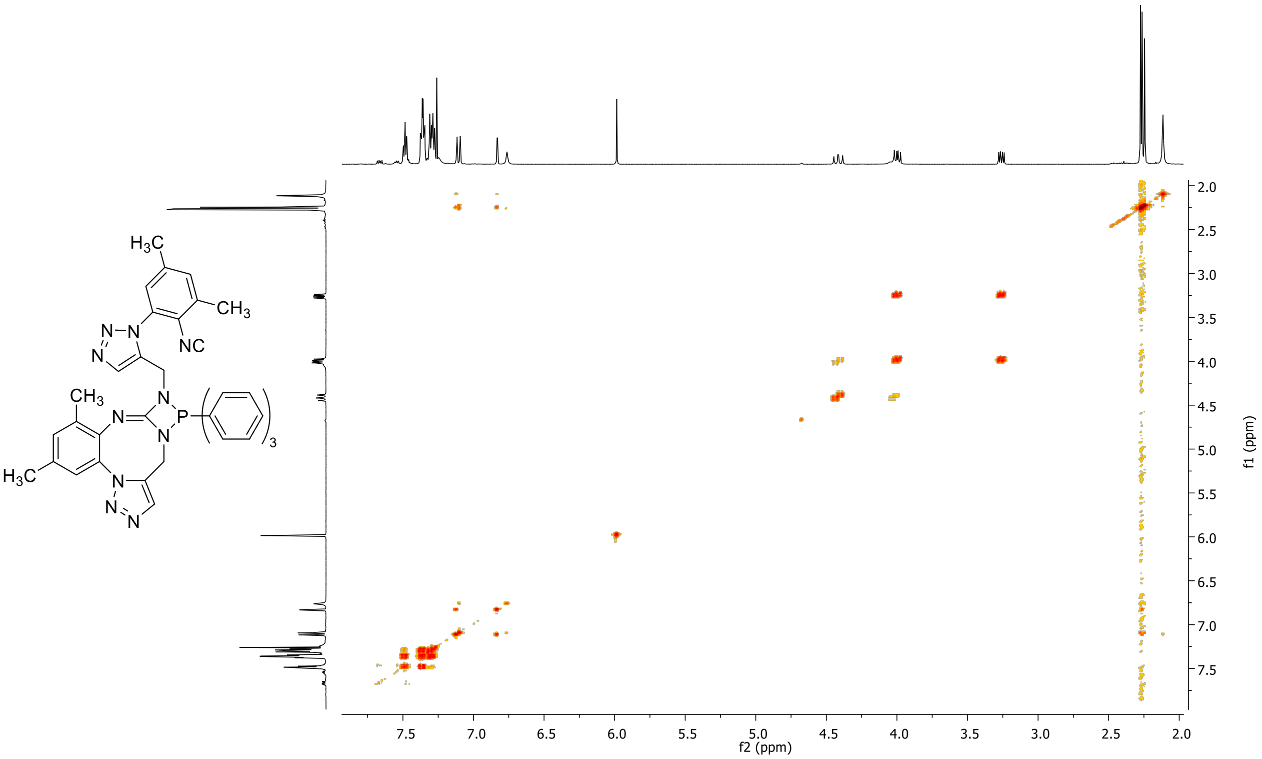

9ba (^{13}C NMR, 150 MHz, $CDCl_3$, 298 K)

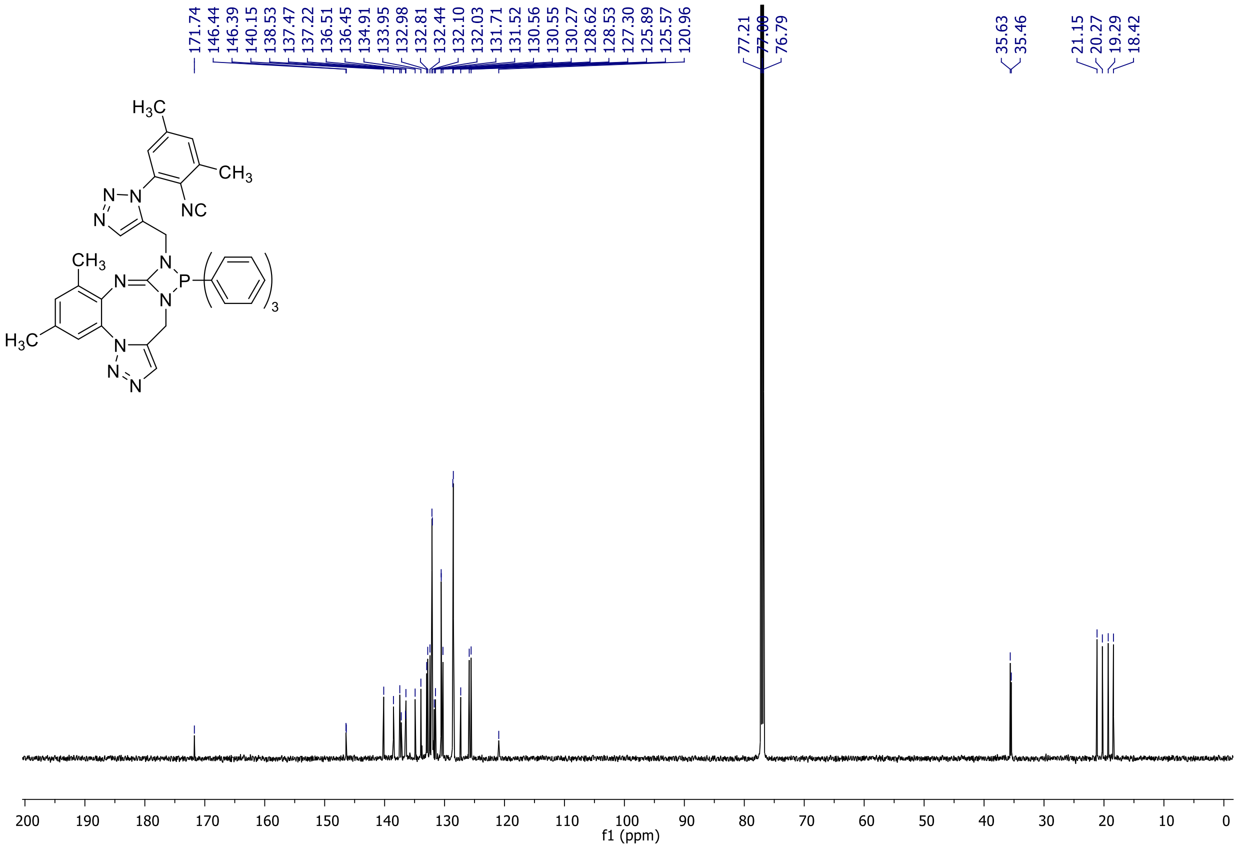

9ba (DEPT-135 NMR, 150 MHz, CDCl$_3$, 298 K)

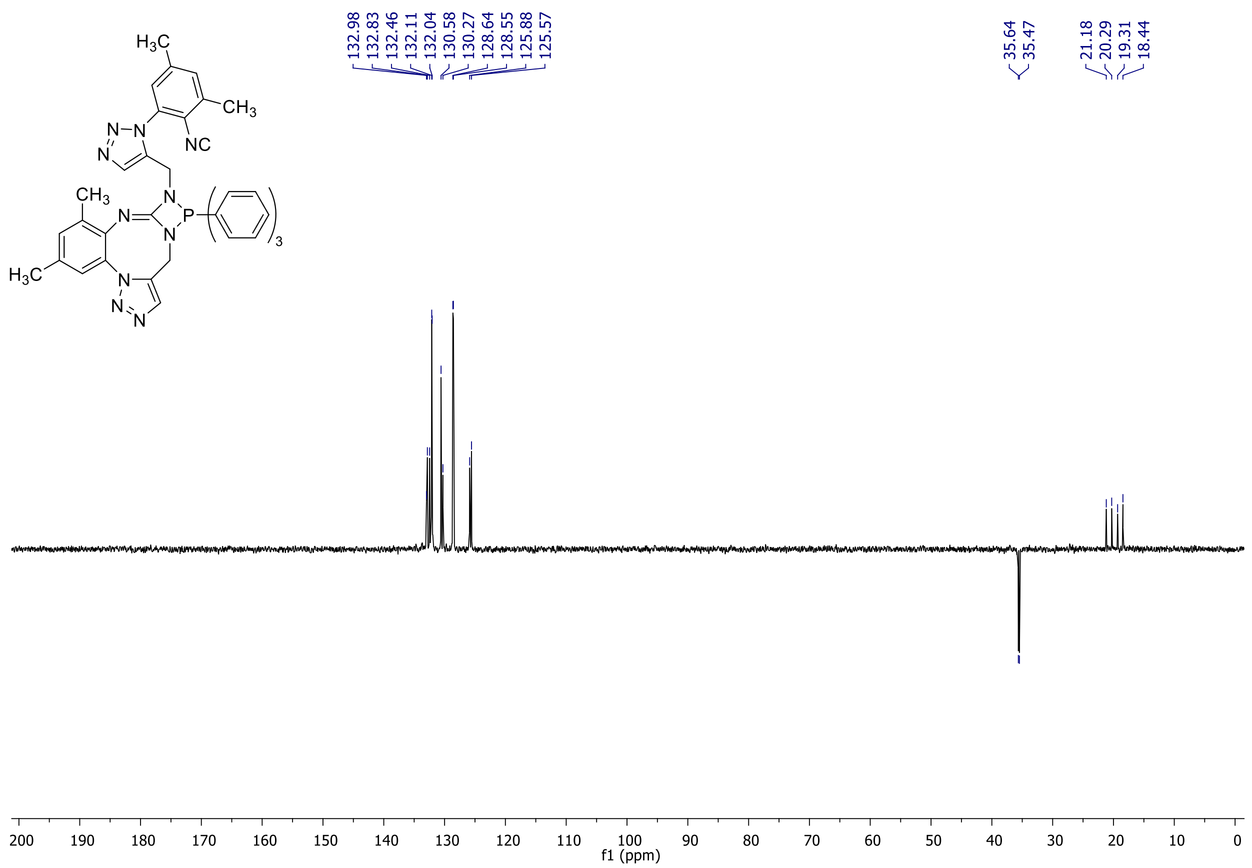

9ba (HMQC NMR, 600 MHz, CDCl$_3$, 298 K)

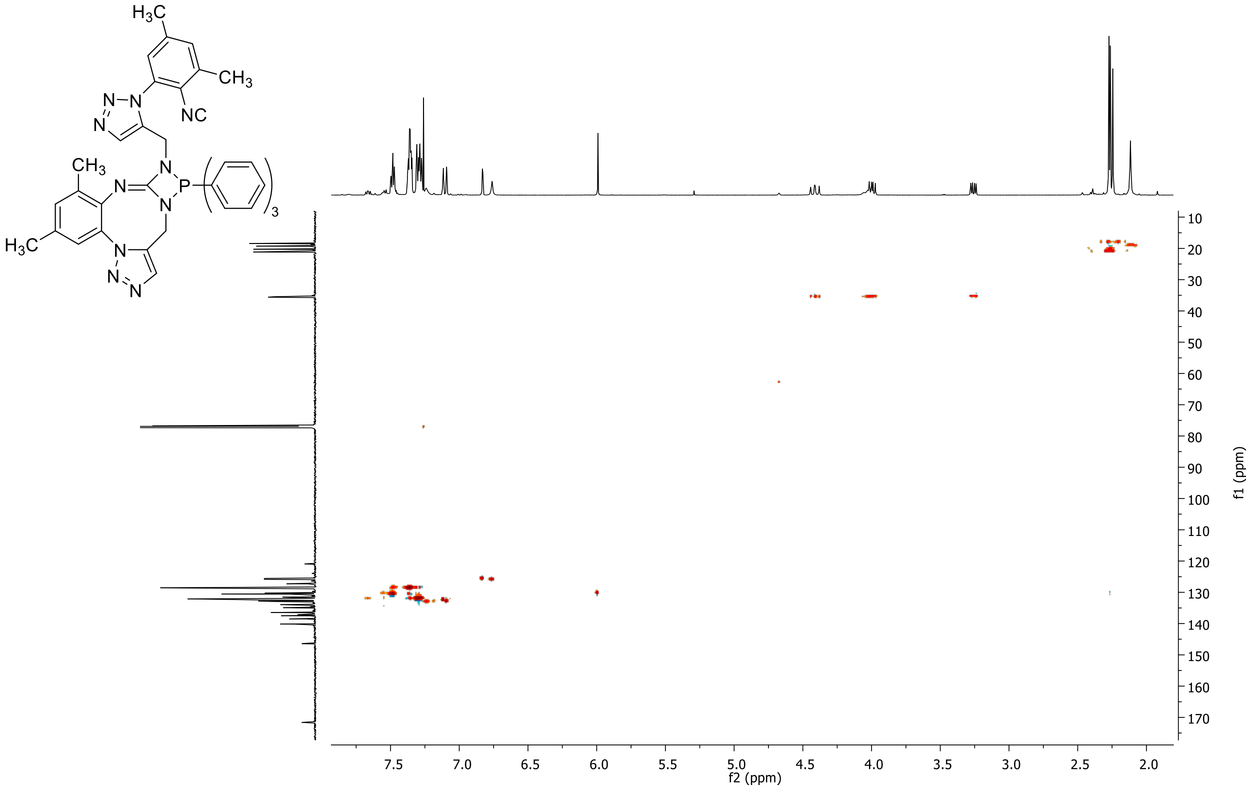

9ba (^{31}P NMR, 121.5 MHz, $CDCl_3$, 298 K)

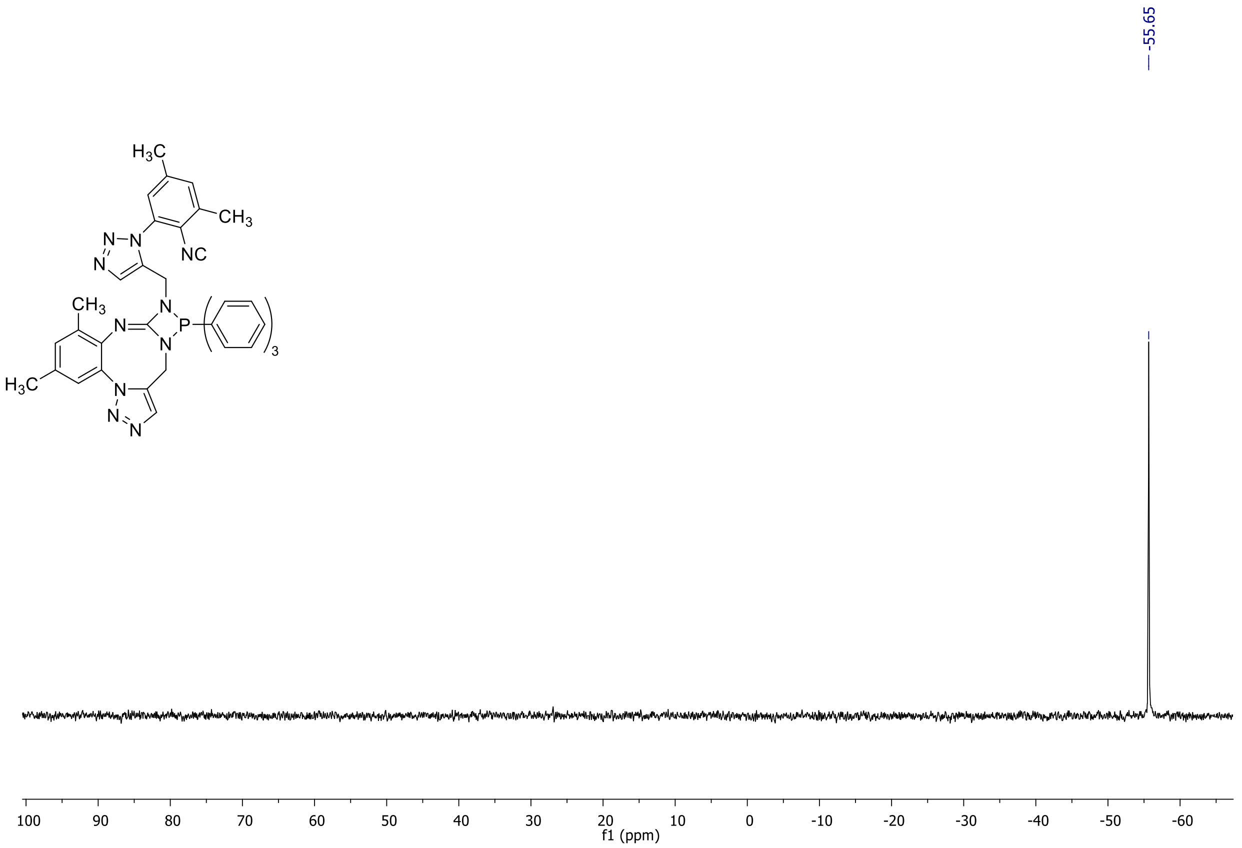

9bb (^{1}H NMR, 600 MHz, $CDCl_3$, 298 K)

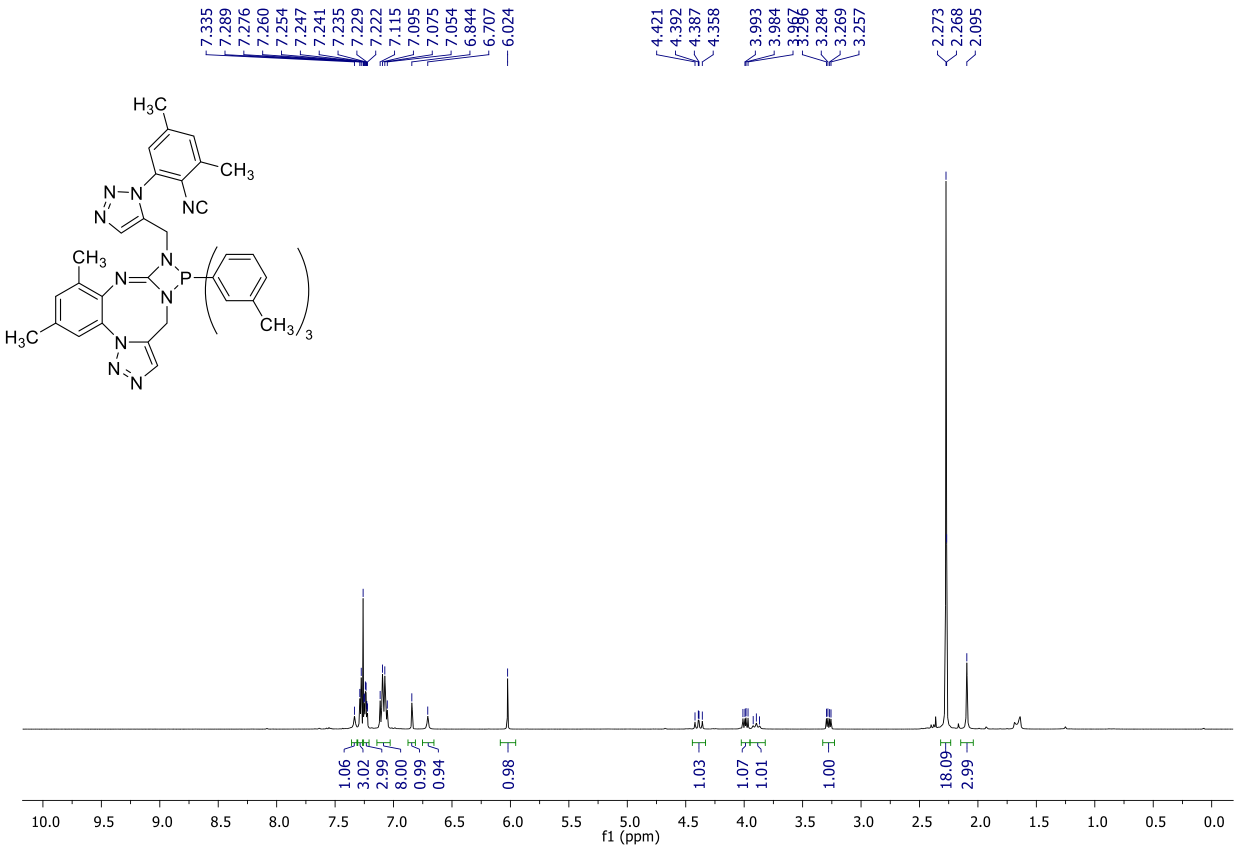

9bb ($^{1}H\{^{31}P\}$ NMR, 600 MHz, $CDCl_3$, 298 K)

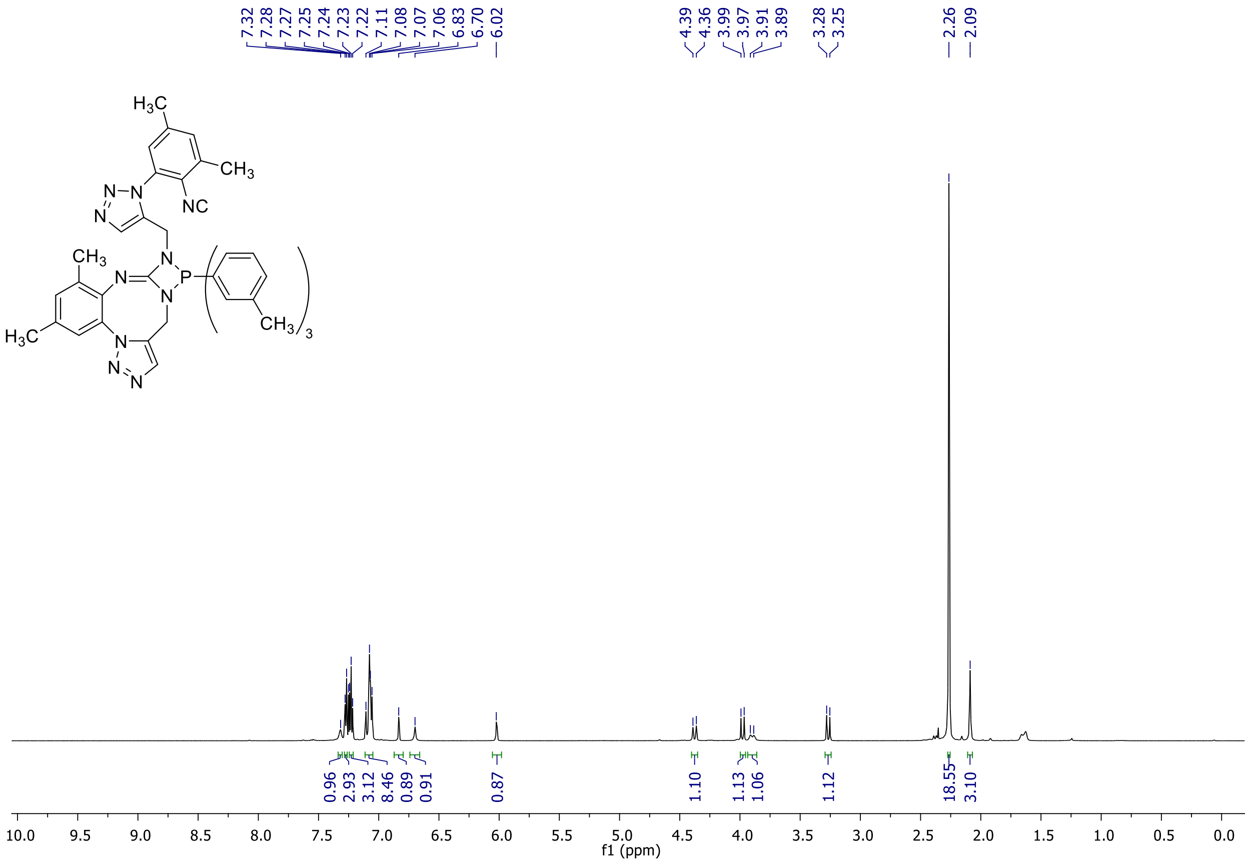

9bb (^{13}C NMR, 150 MHz, $CDCl_3$, 298 K)

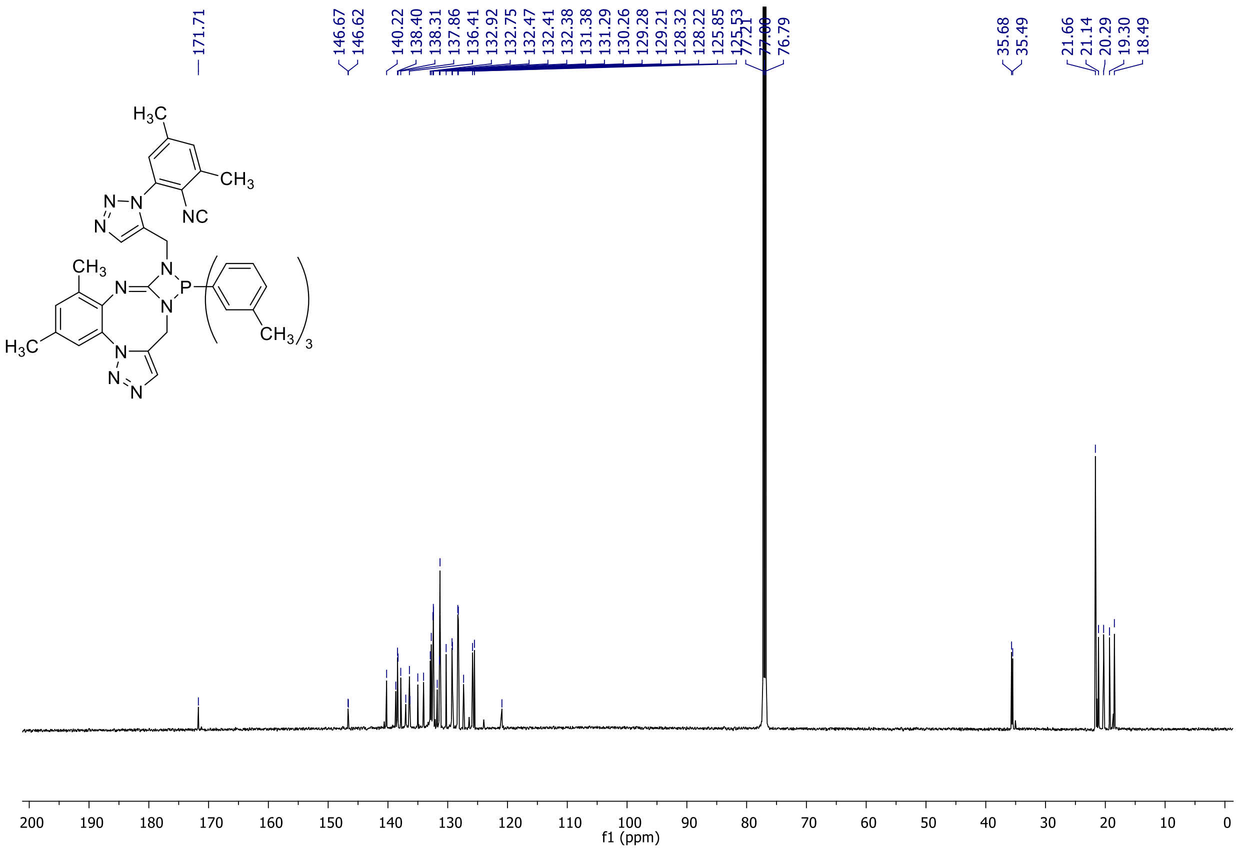

9bb (DEPT-135 NMR, 150 MHz, $CDCl_3$, 298 K)

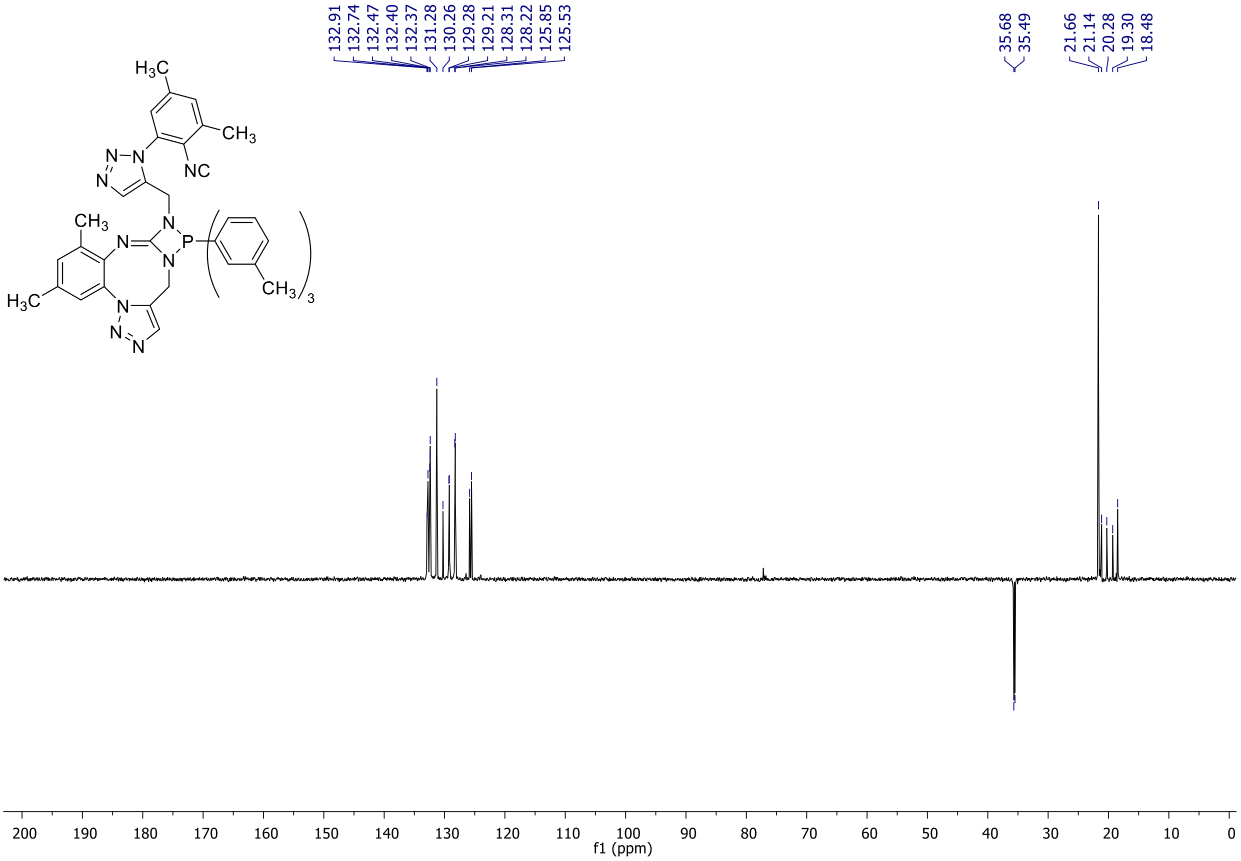

9bb (^{31}P NMR, 243 MHz, $CDCl_3$, 298 K)

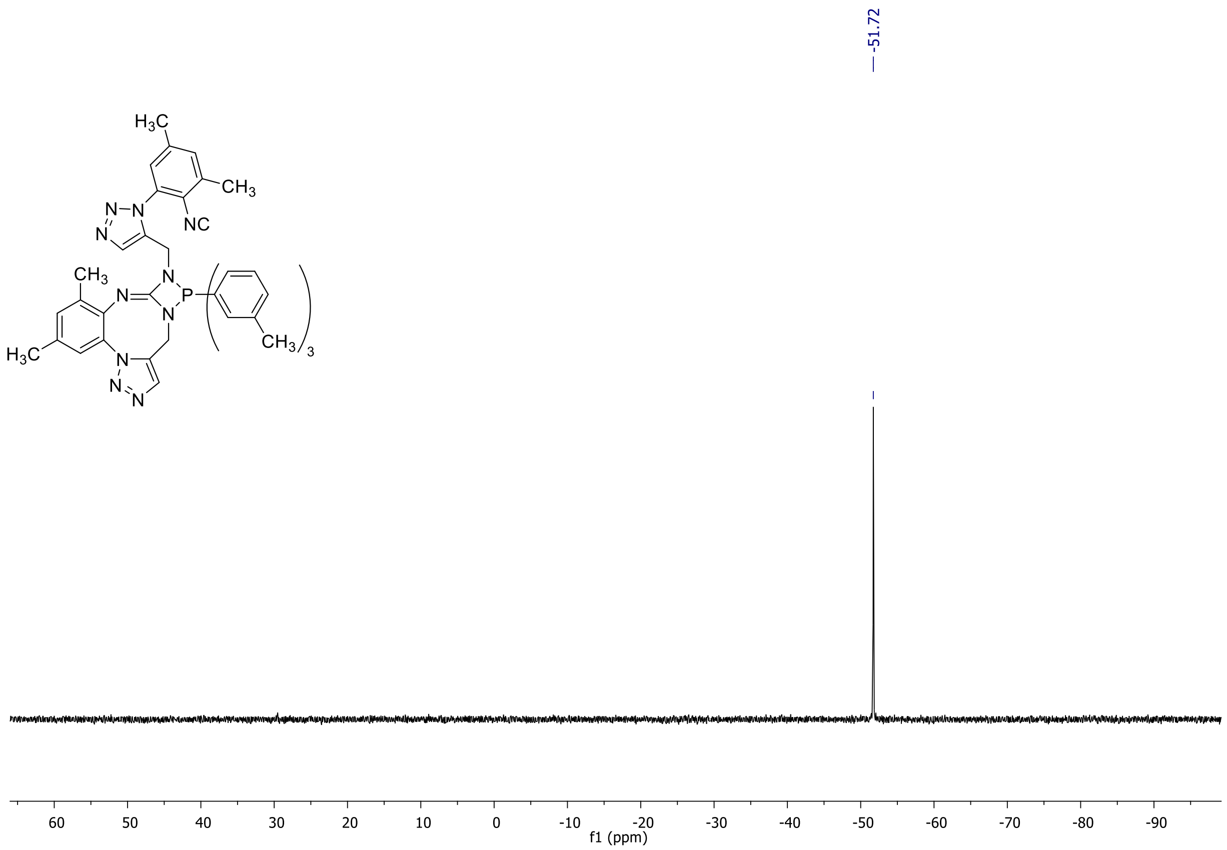

9bd (^{1}H NMR, 600 MHz, $CDCl_3$, 298 K)

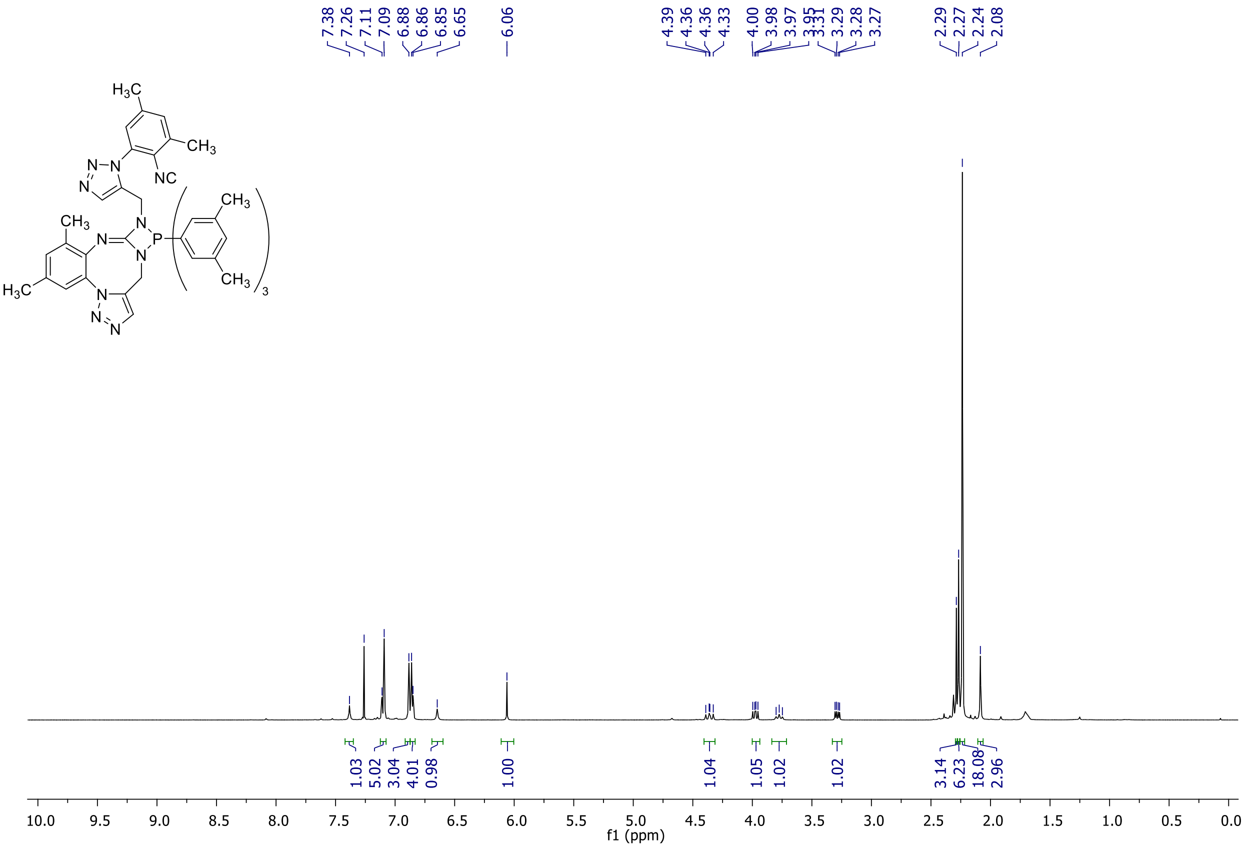

9bd (^{13}C NMR, 150 MHz, $CDCl_3$, 298 K)

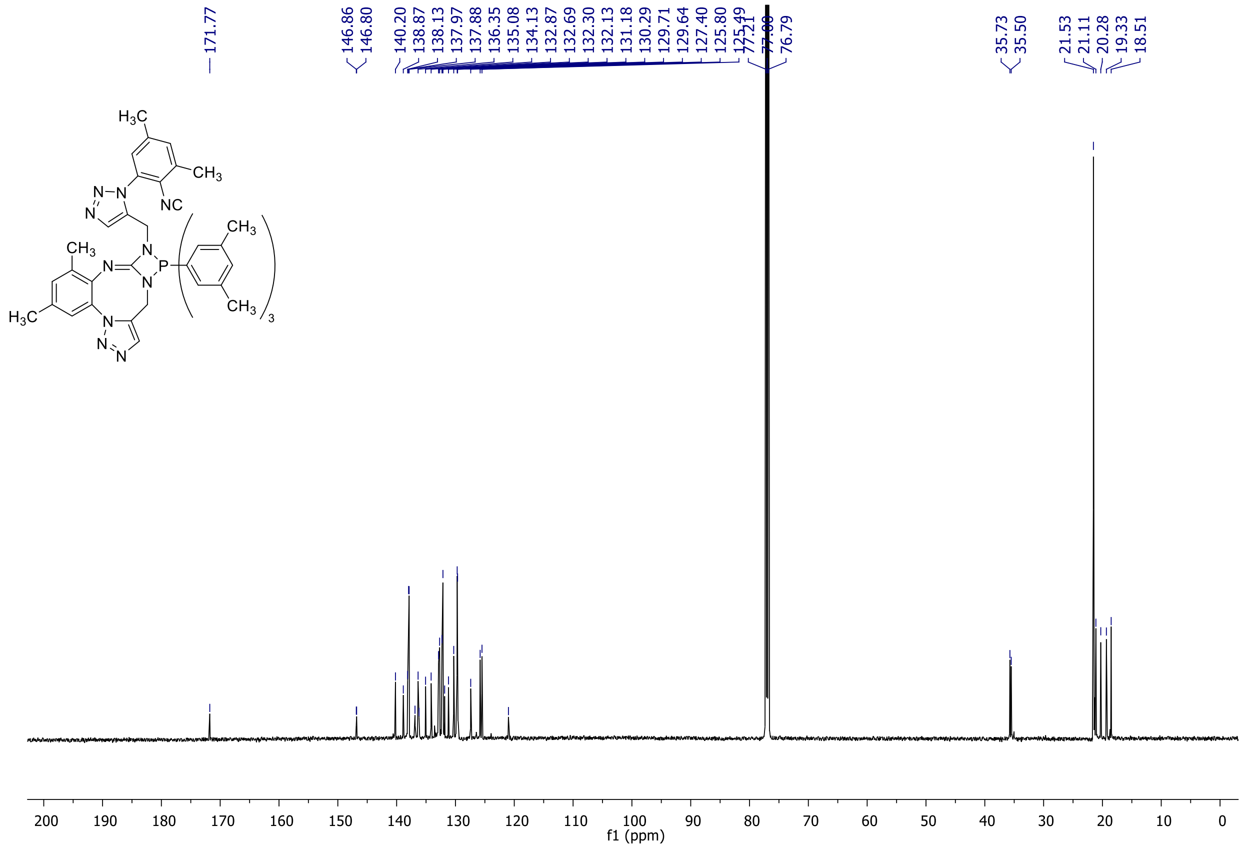

9bd (DEPT-135 NMR, 150 MHz, $CDCl_3$, 298 K)

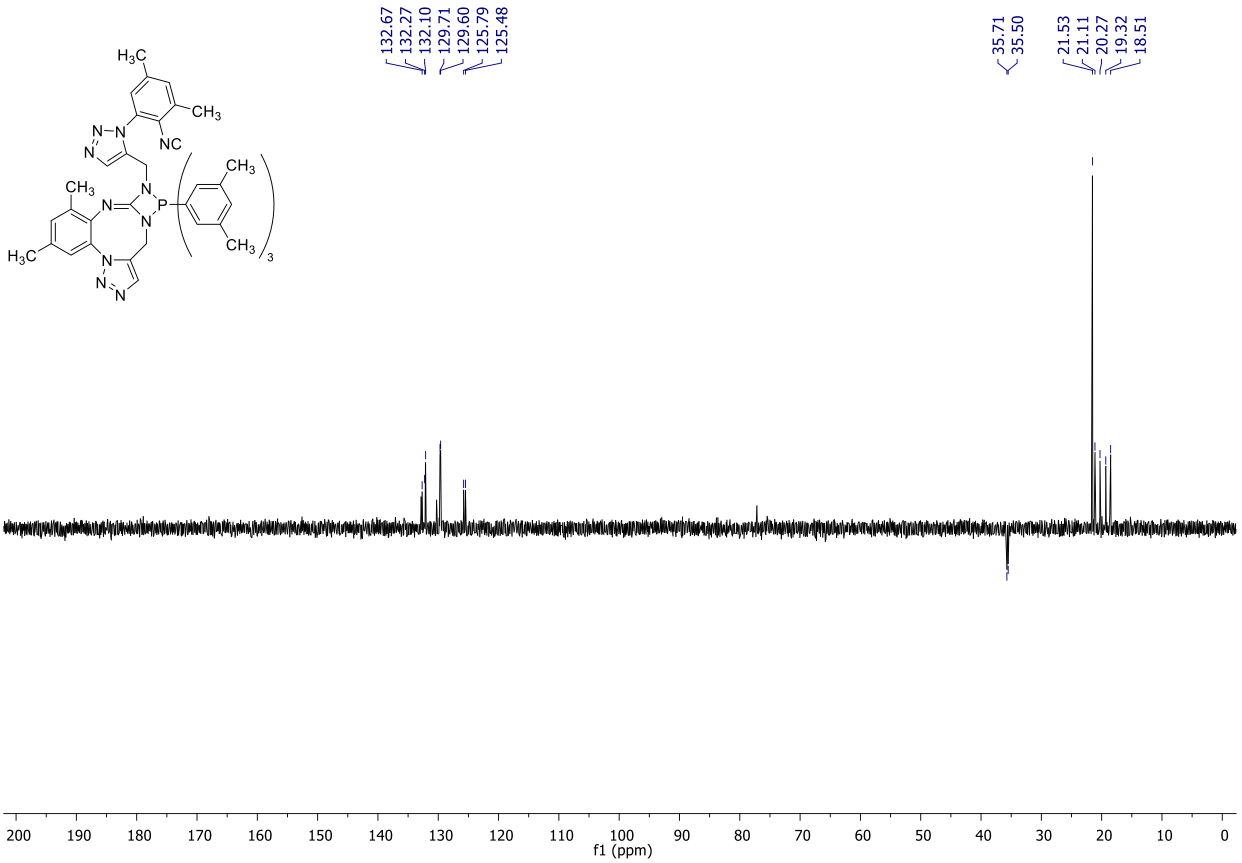

9bd (^{31}P NMR, 243 MHz, $CDCl_3$, 298 K)

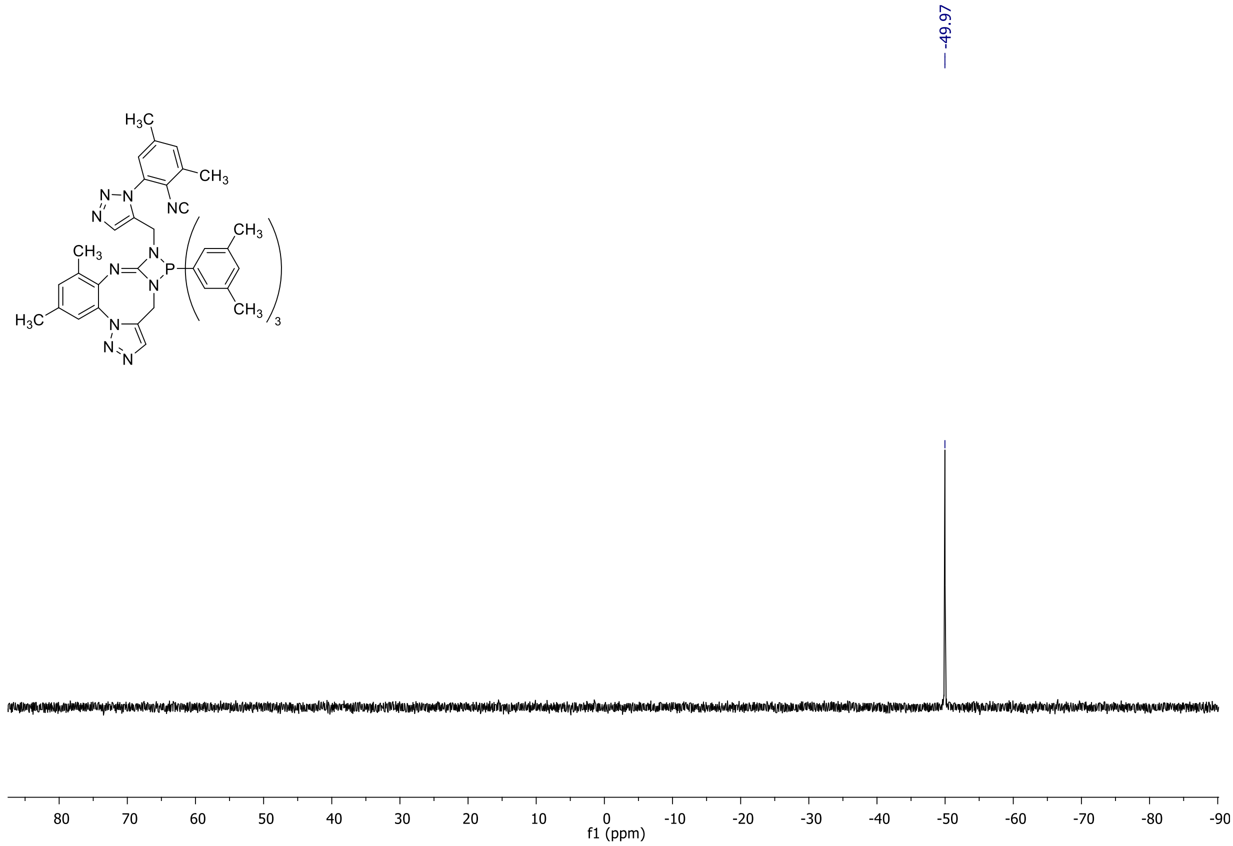

9be (^{1}H NMR, 600 MHz, $CDCl_3$, 298 K)

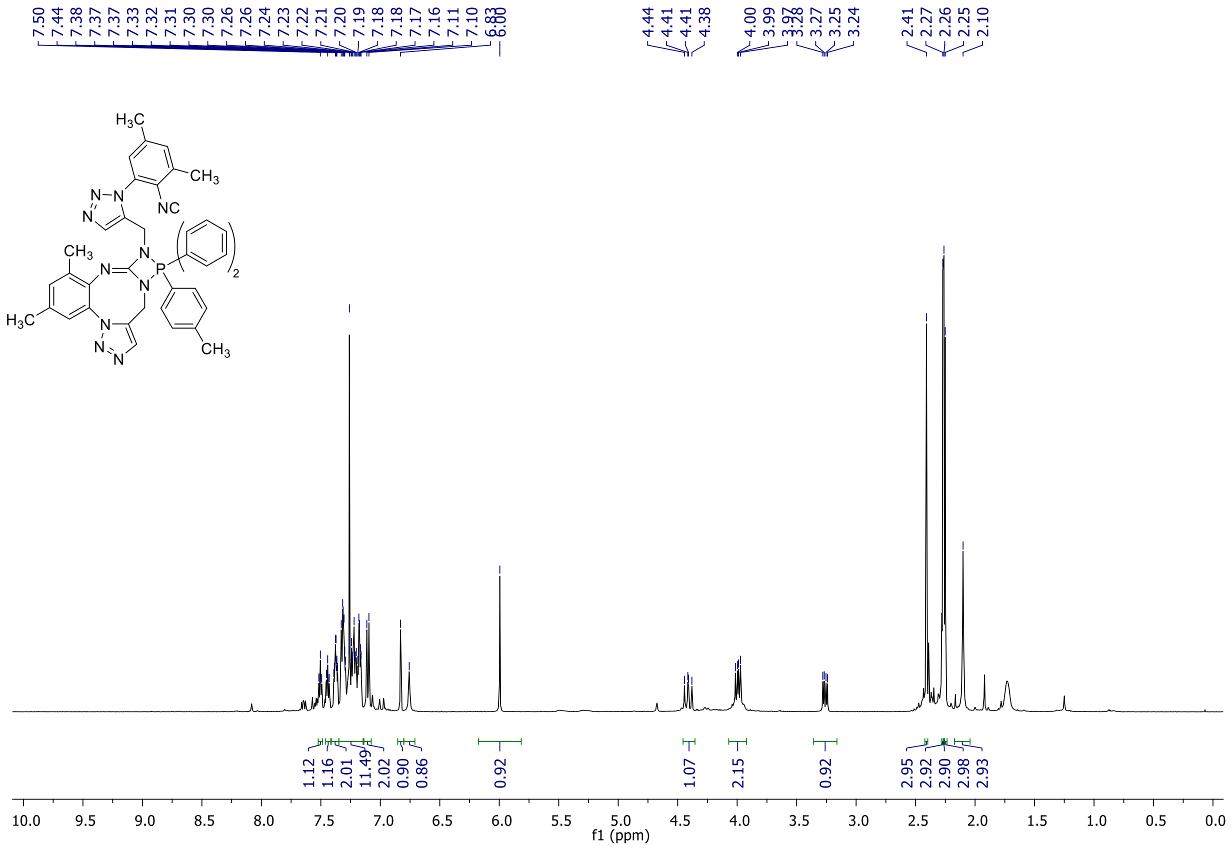

9be ($^{1}H\{^{31}P\}$ NMR, 600 MHz, $CDCl_3$, 298 K)

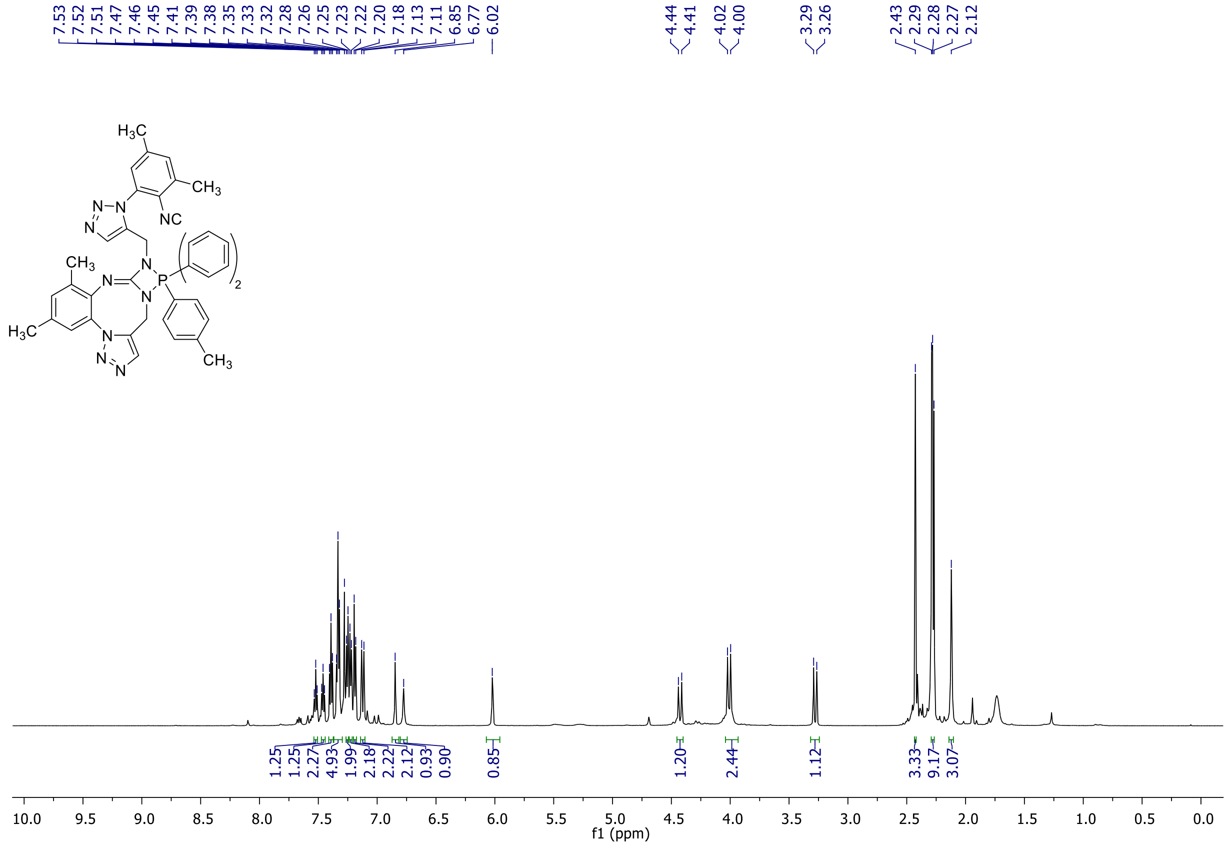

9be (^{13}C NMR, 150 MHz, $CDCl_3$, 298 K)

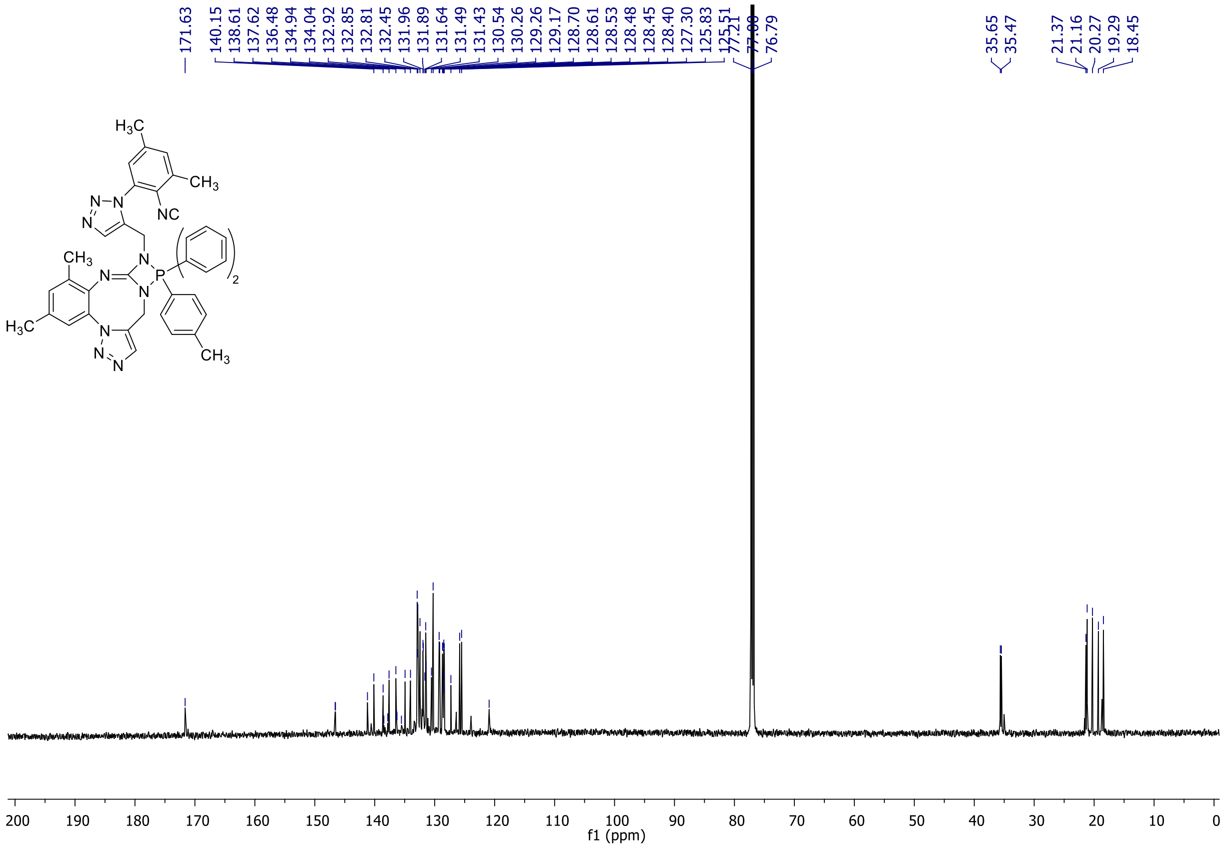

9be (DEPT-135 NMR, 150 MHz, $CDCl_3$, 298 K)

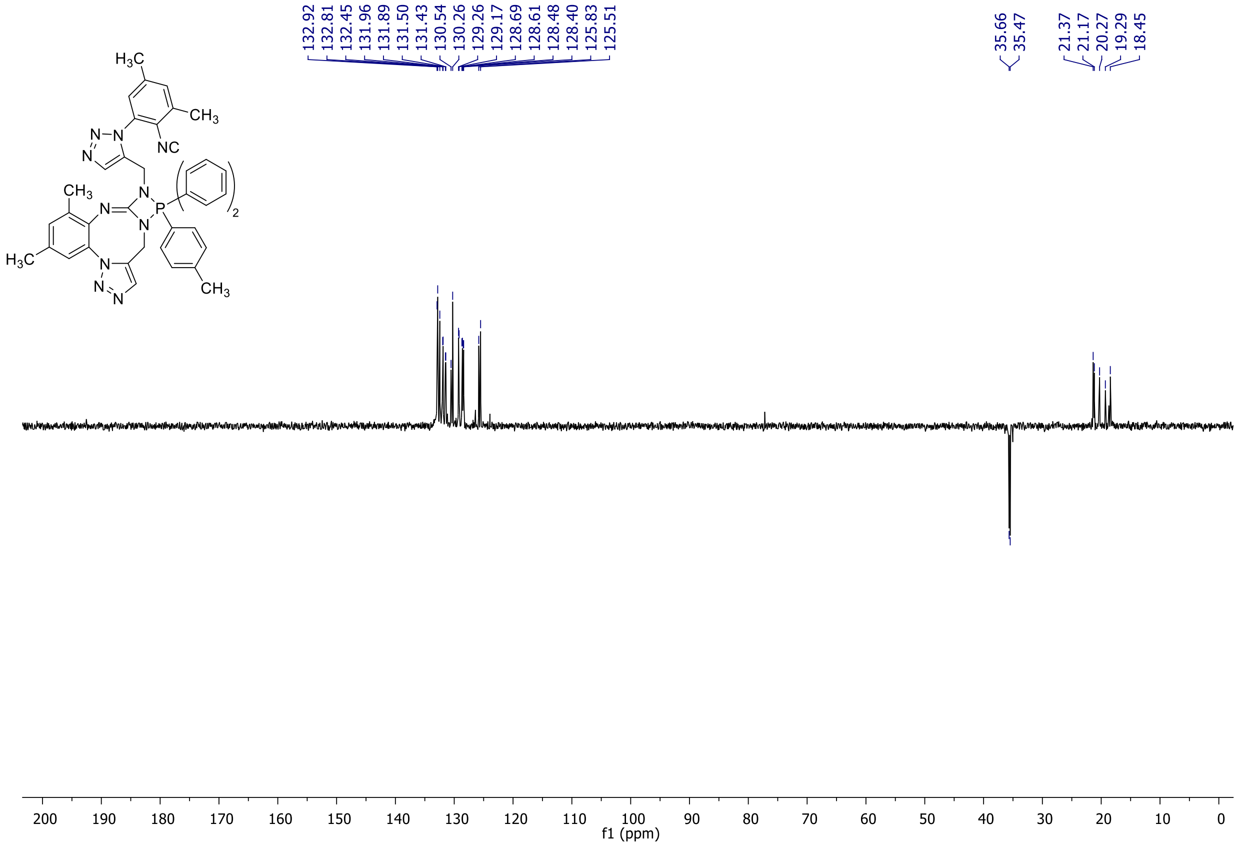

9be (^{31}P NMR, 243 MHz, $CDCl_3$, 298 K)

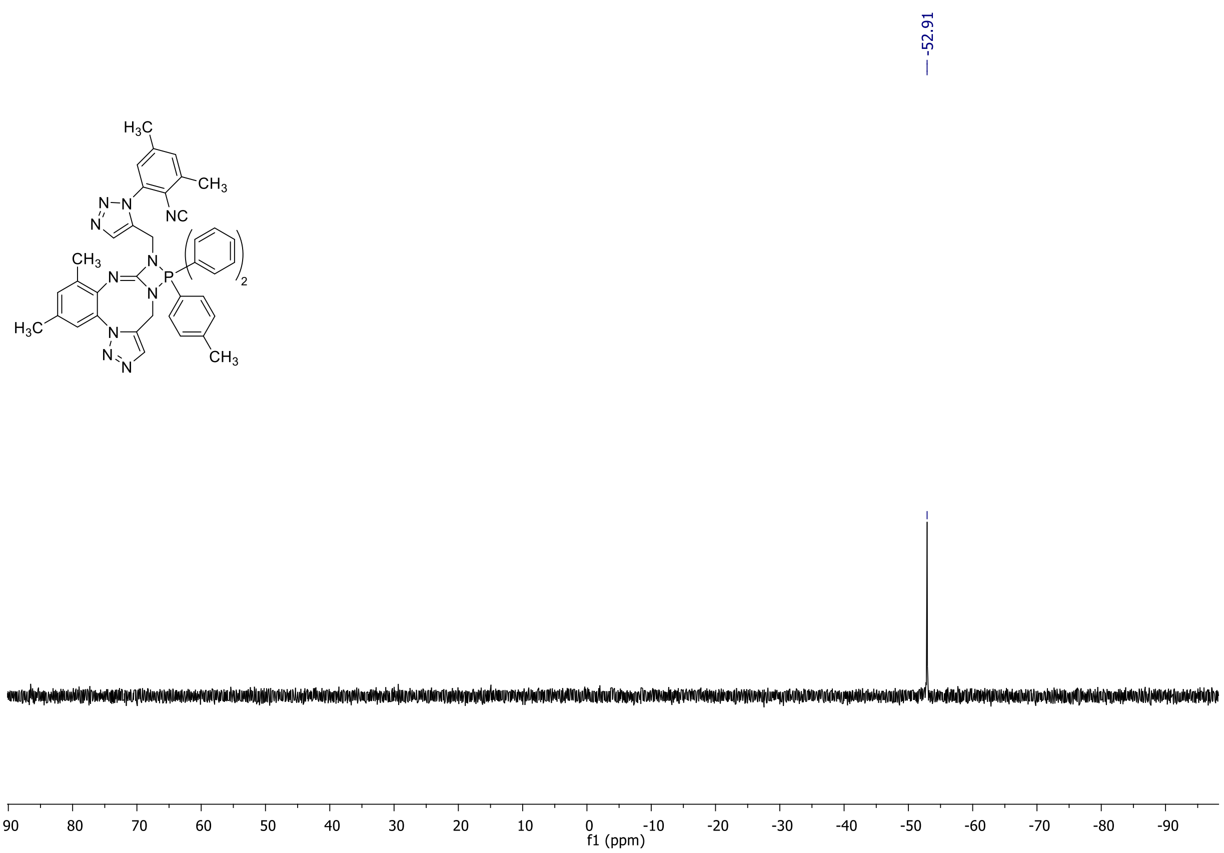

9be (^{31}P NMR, 243 MHz, $CDCl_3$, 298 K, after 6 h)

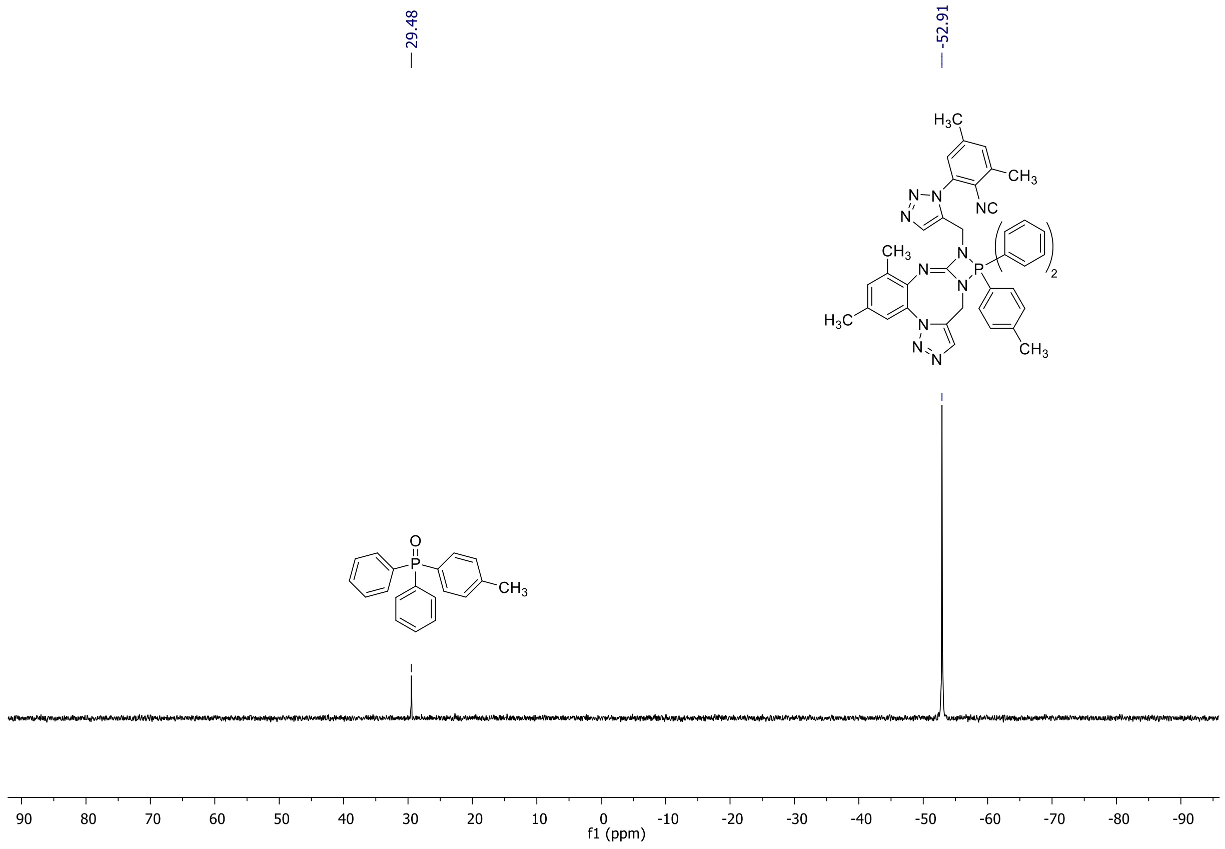

9ca (^1H NMR, 600 MHz, $CDCl_3$, 298 K)

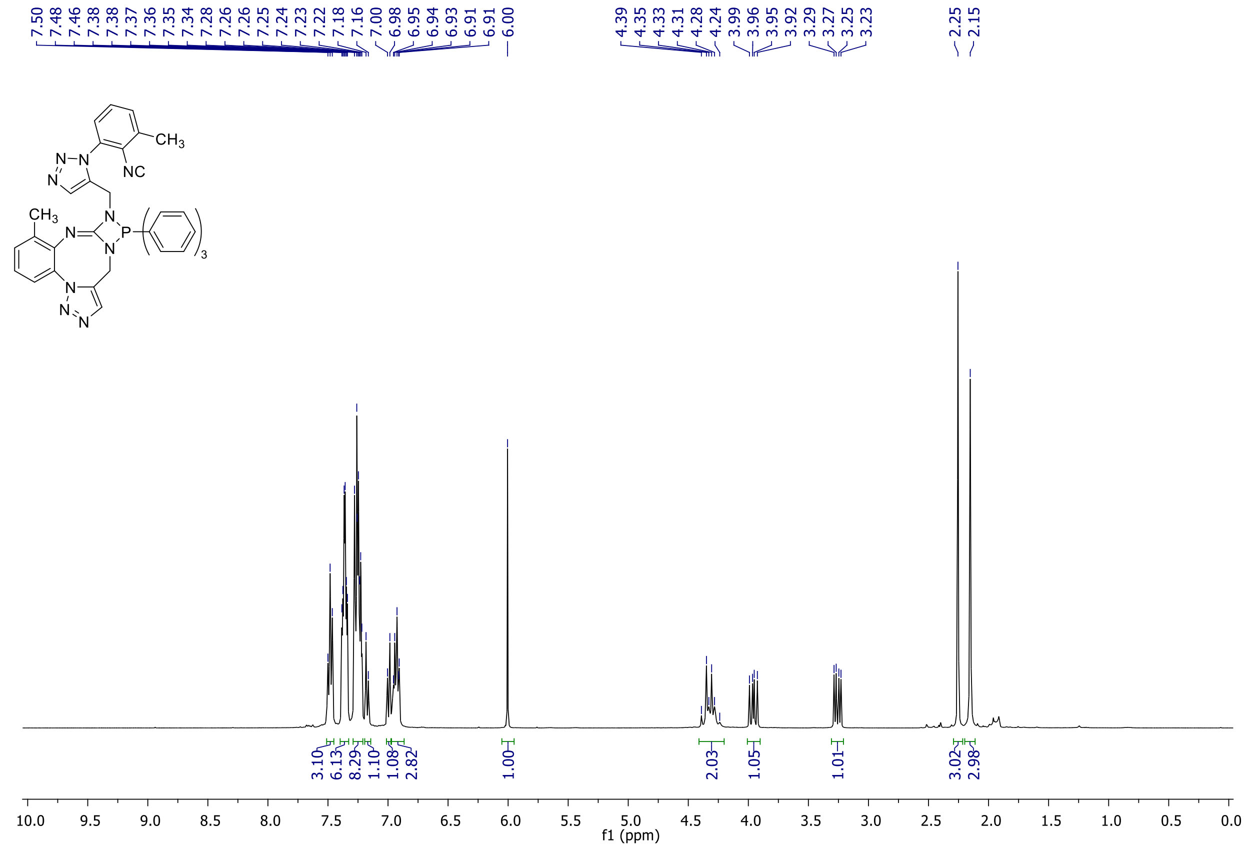

9ca ($^1H\{^{31}P\}$ NMR, 600 MHz, $CDCl_3$, 298 K)

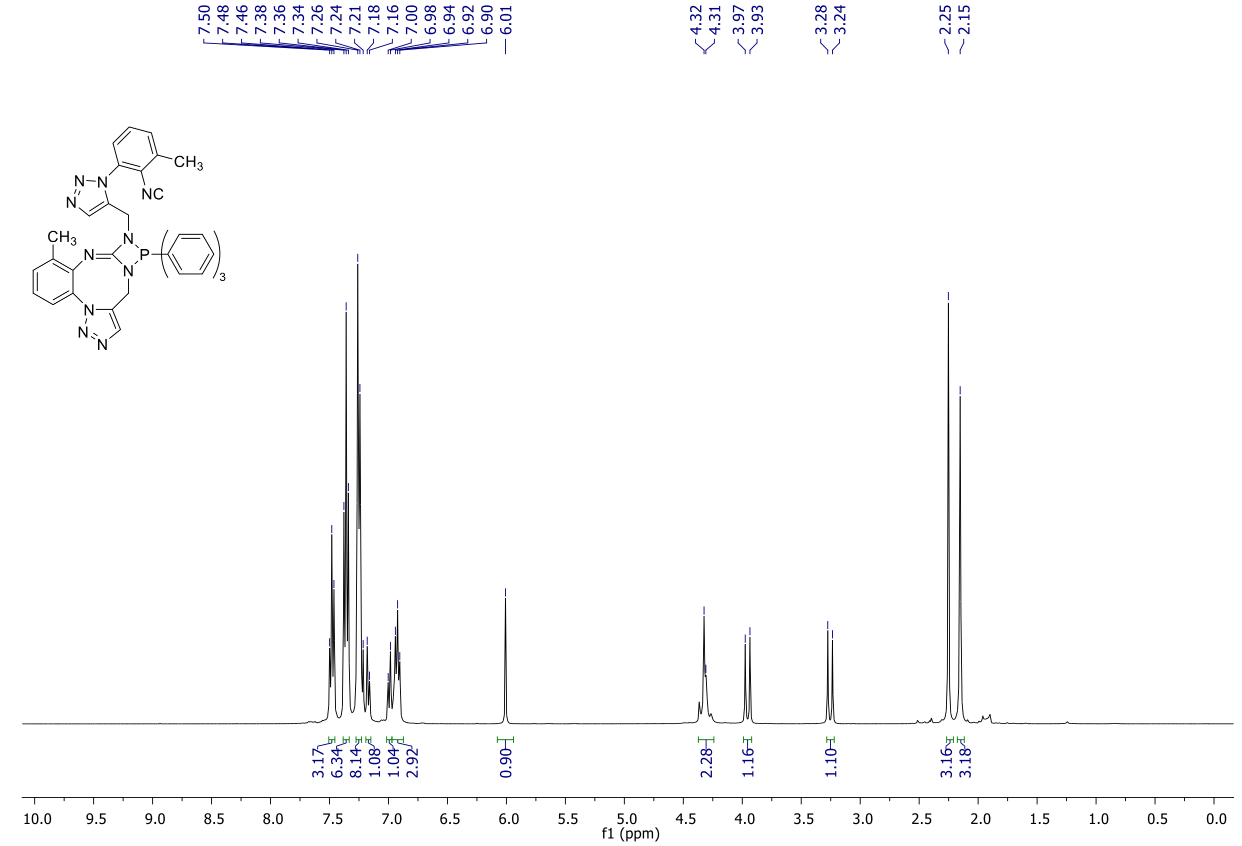

9ca (^{13}C NMR, 75 MHz, $CDCl_3$, 298 K)

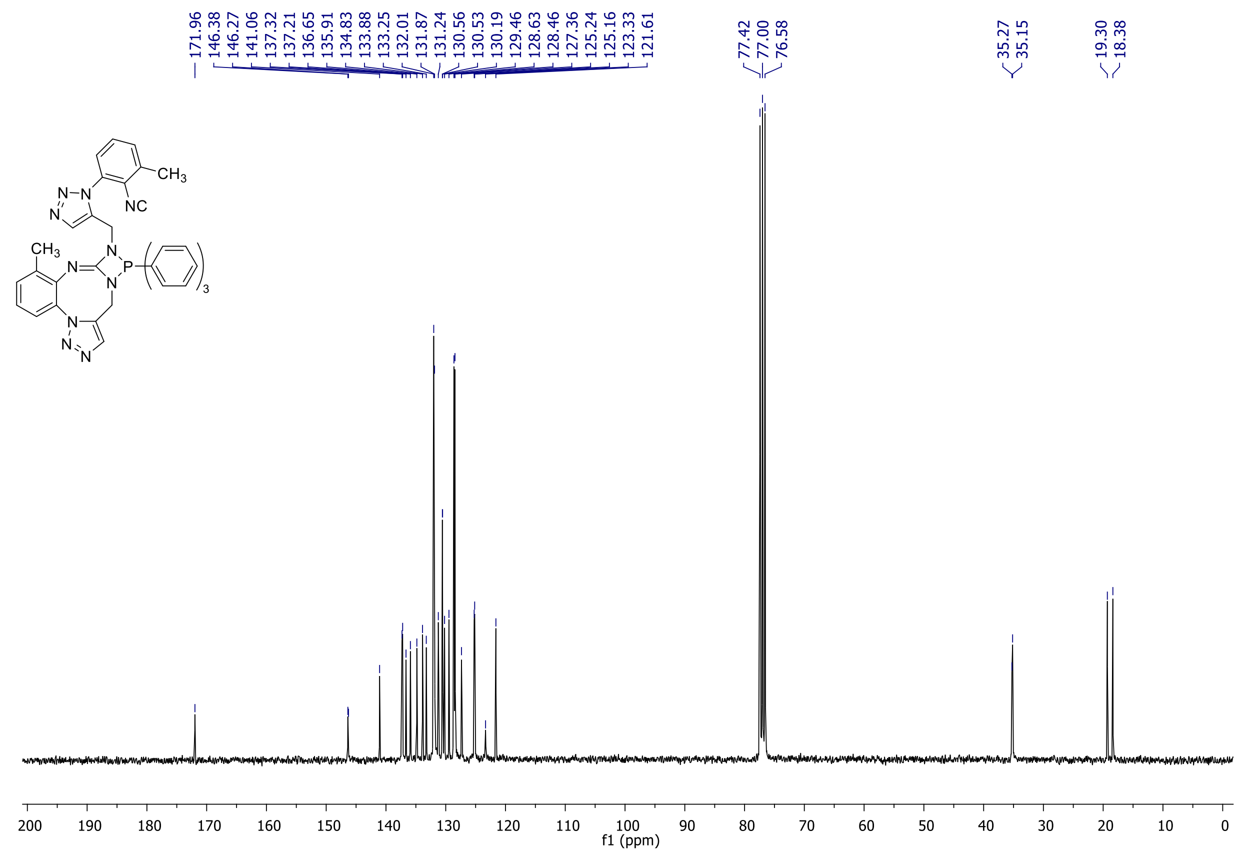

9ca (DEPT-135 NMR, 75 MHz, $CDCl_3$, 298 K, *Et_2O)

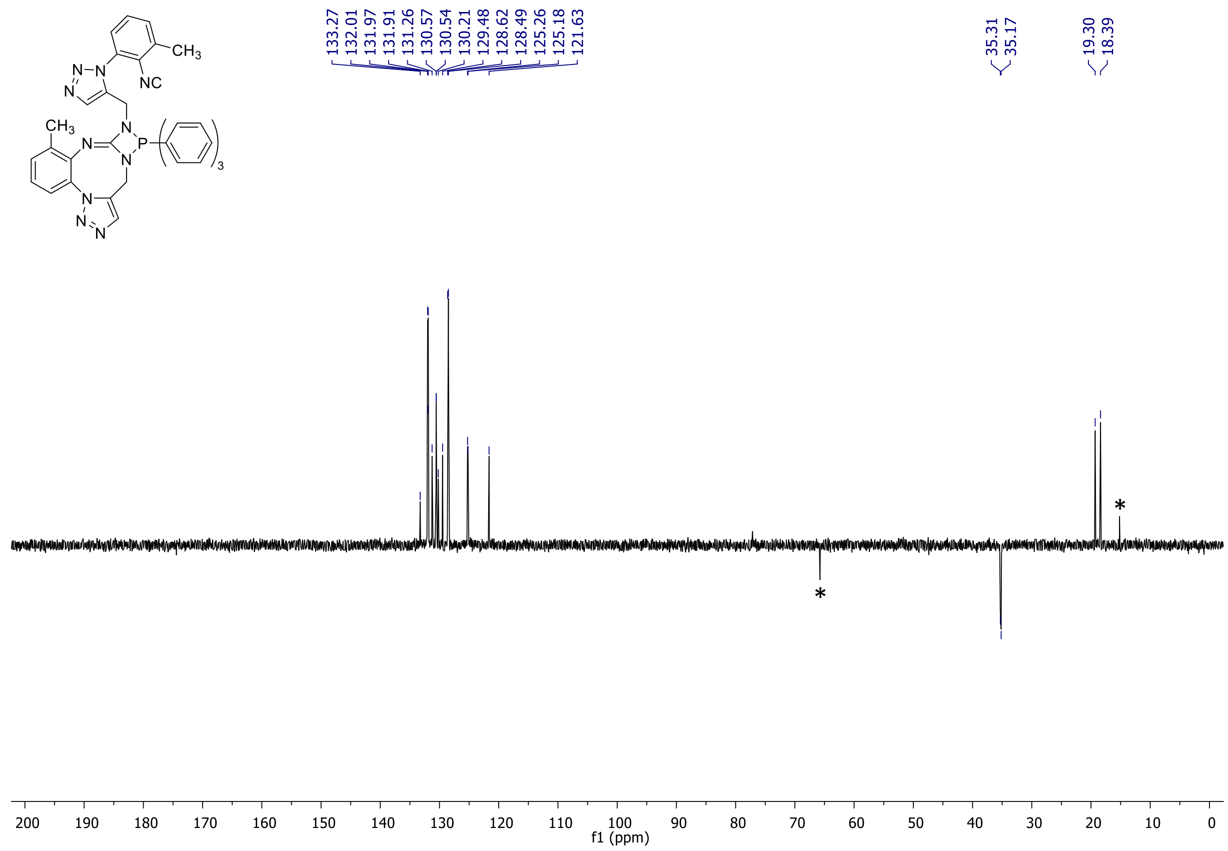

9ca (^{31}P NMR, 243 MHz, $CDCl_3$, 298 K)

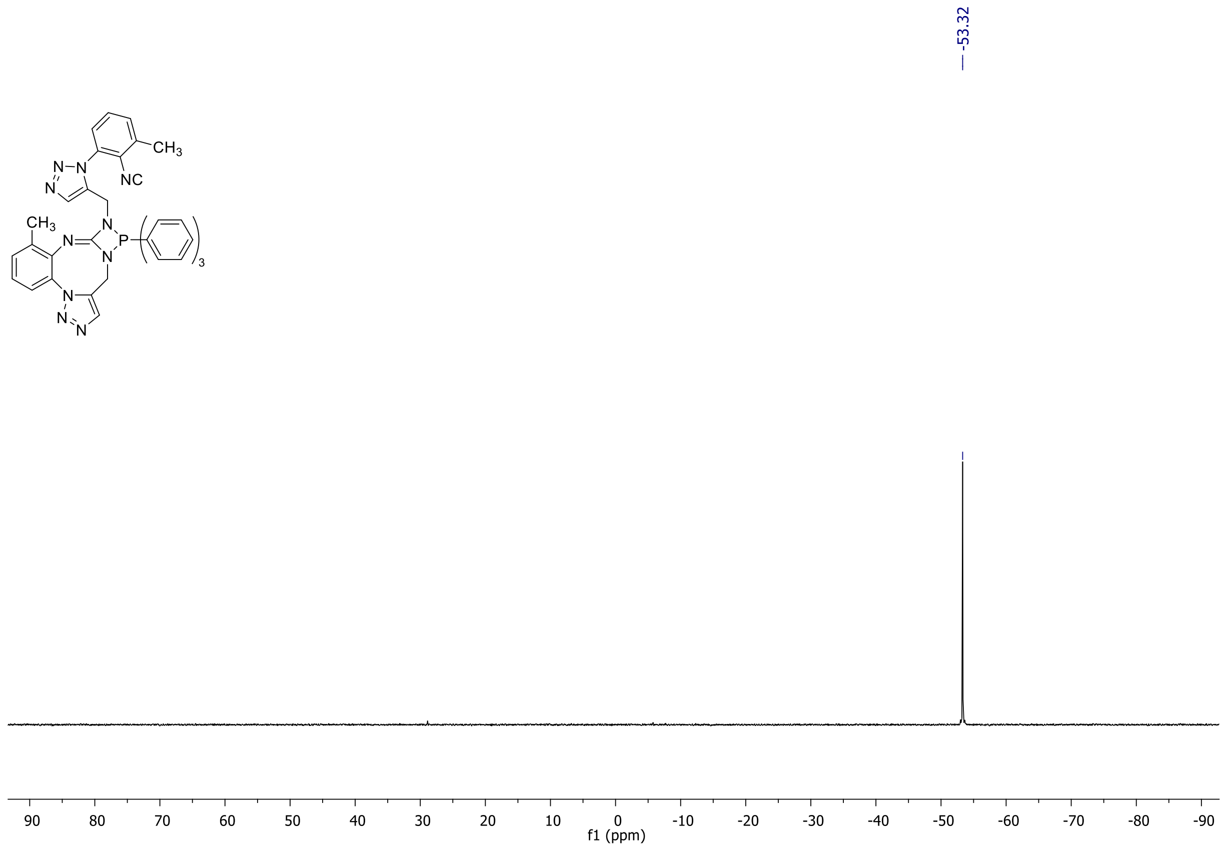

9cb (^{1}H NMR, 600 MHz, $CDCl_3$, 298 K)

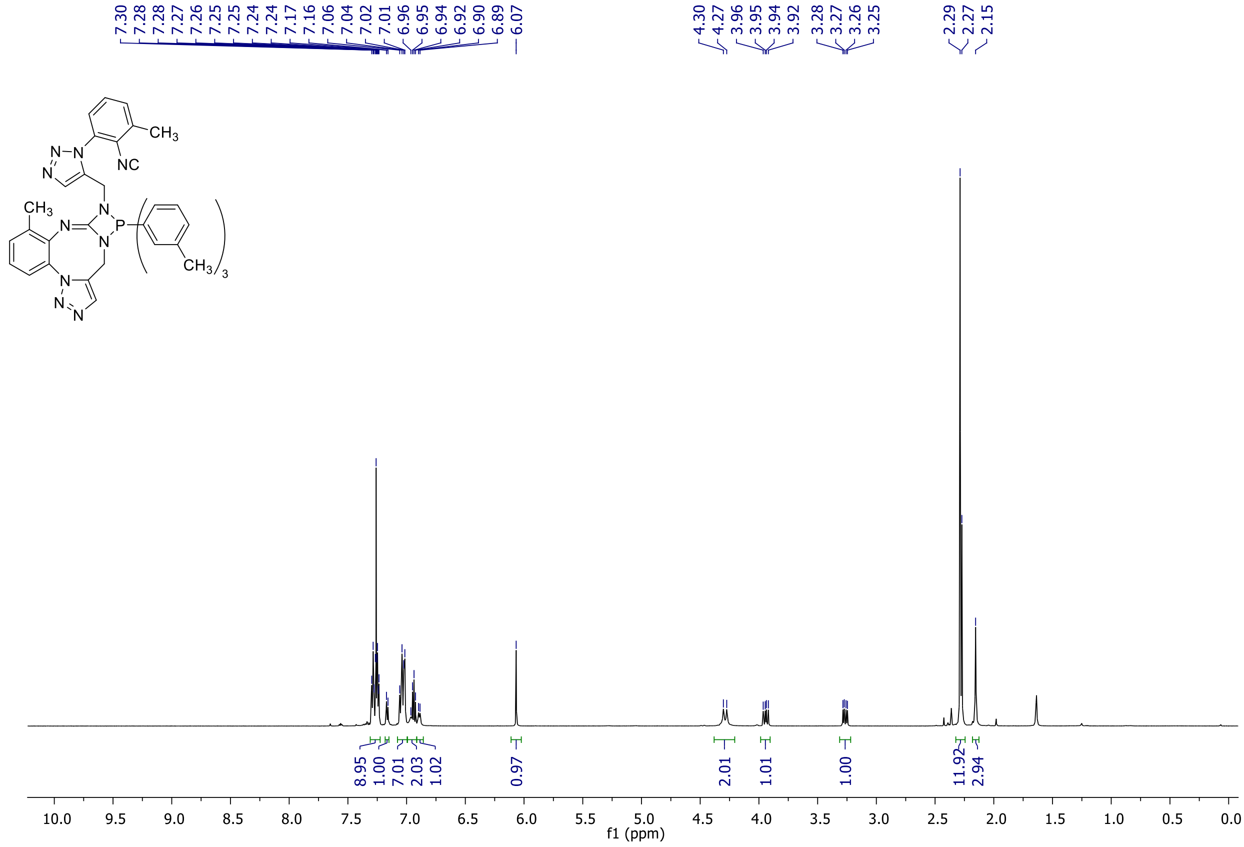

9cb ($^1H\{^{31}P\}$ NMR, 600 MHz, $CDCl_3$, 298 K)

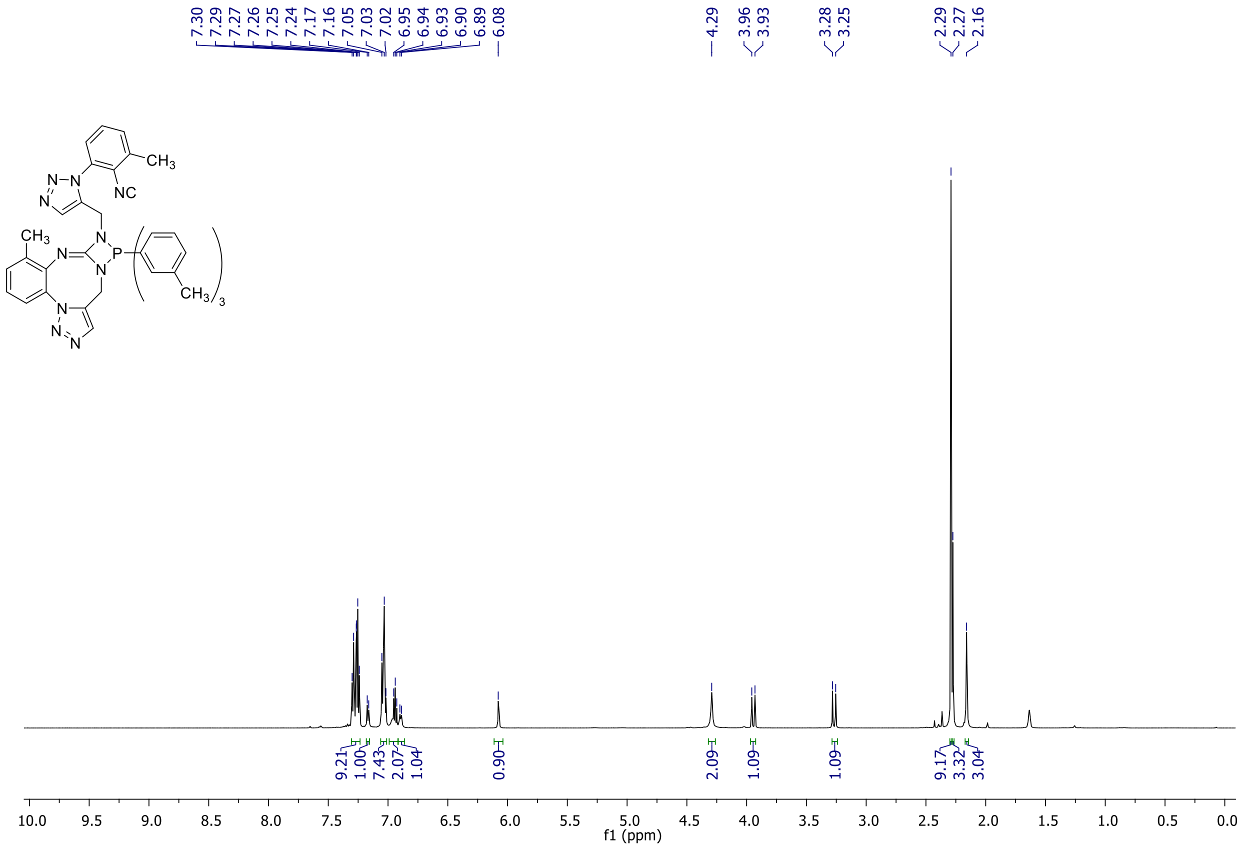

9cb (^{13}C NMR, 150 MHz, $CDCl_3$, 298 K)

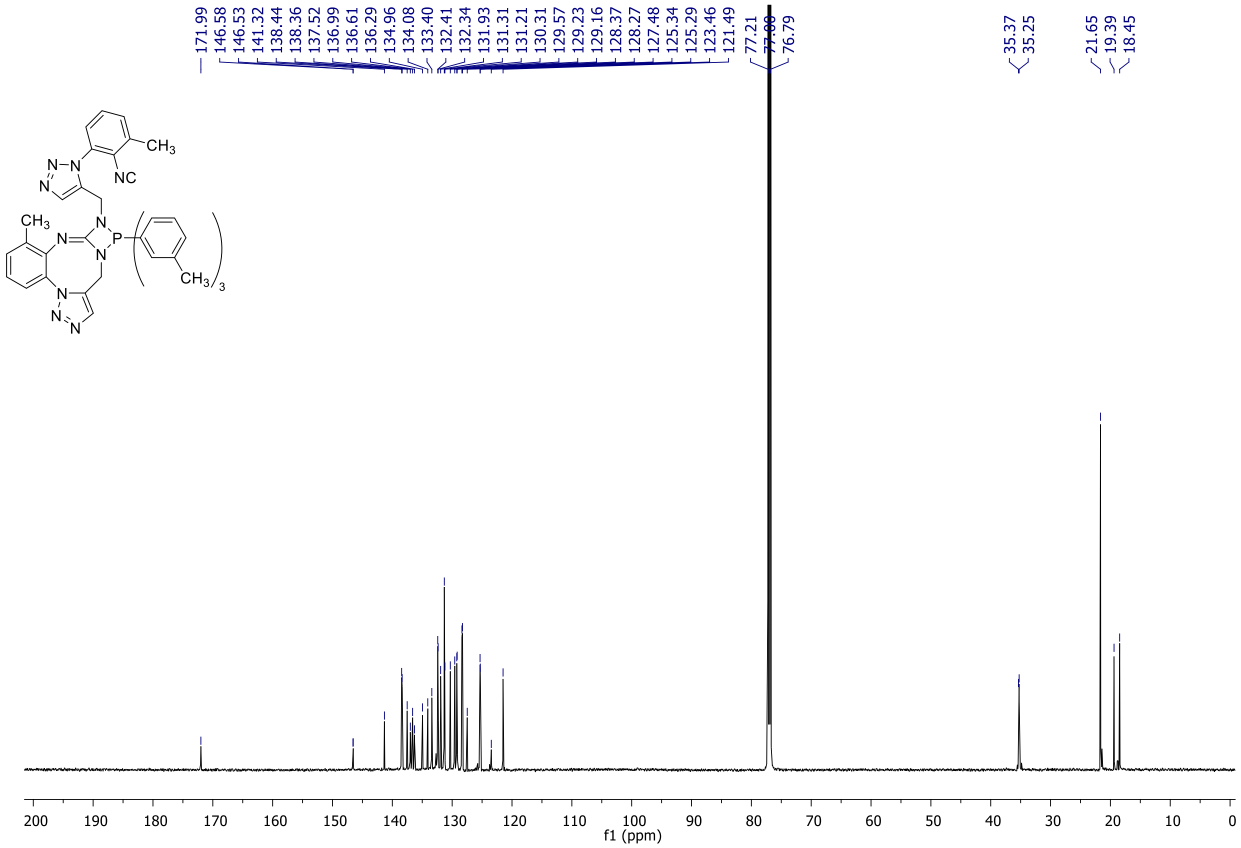

9cb (DEPT-135 NMR, 150 MHz, $CDCl_3$, 298 K)

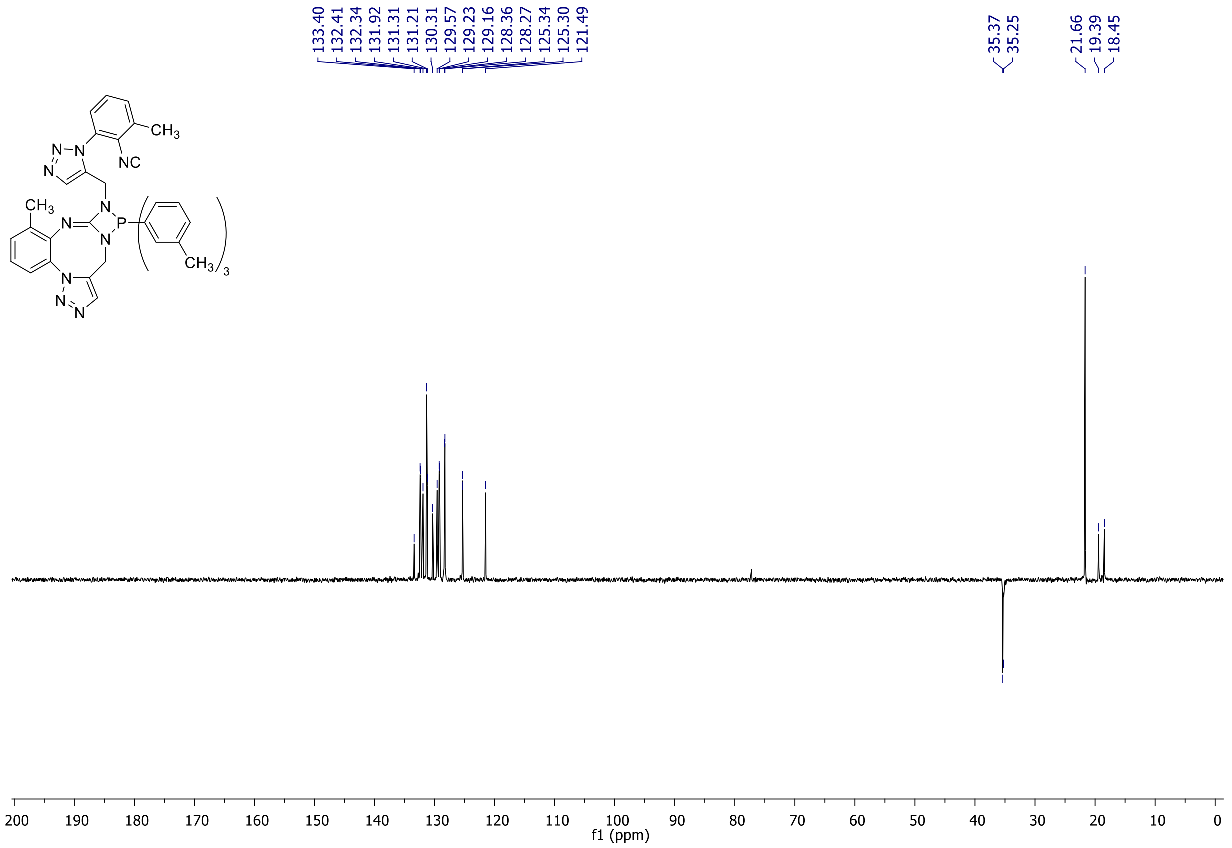

9cb (^{31}P NMR, 243 MHz, $CDCl_3$, 298 K)

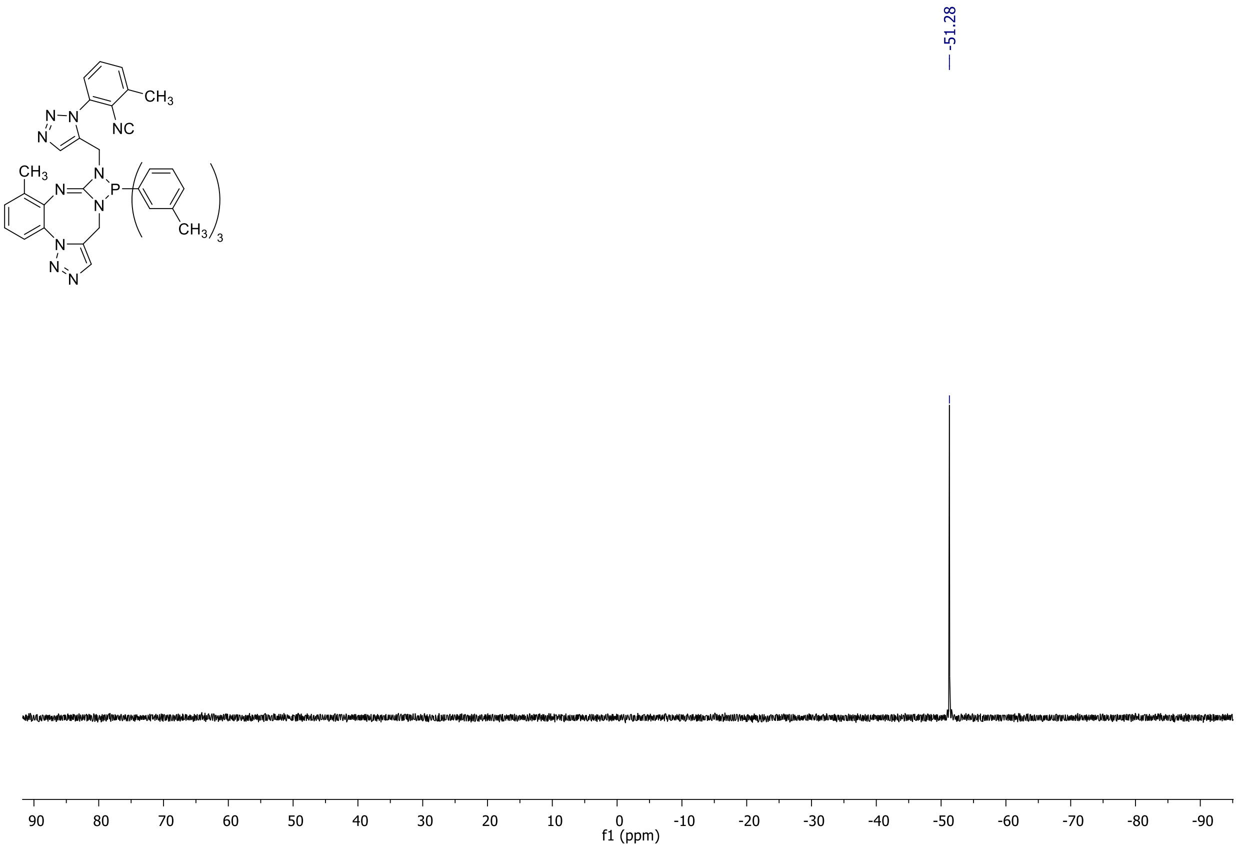

9cd (^{1}H NMR, 600 MHz, $CDCl_3$, 298 K)

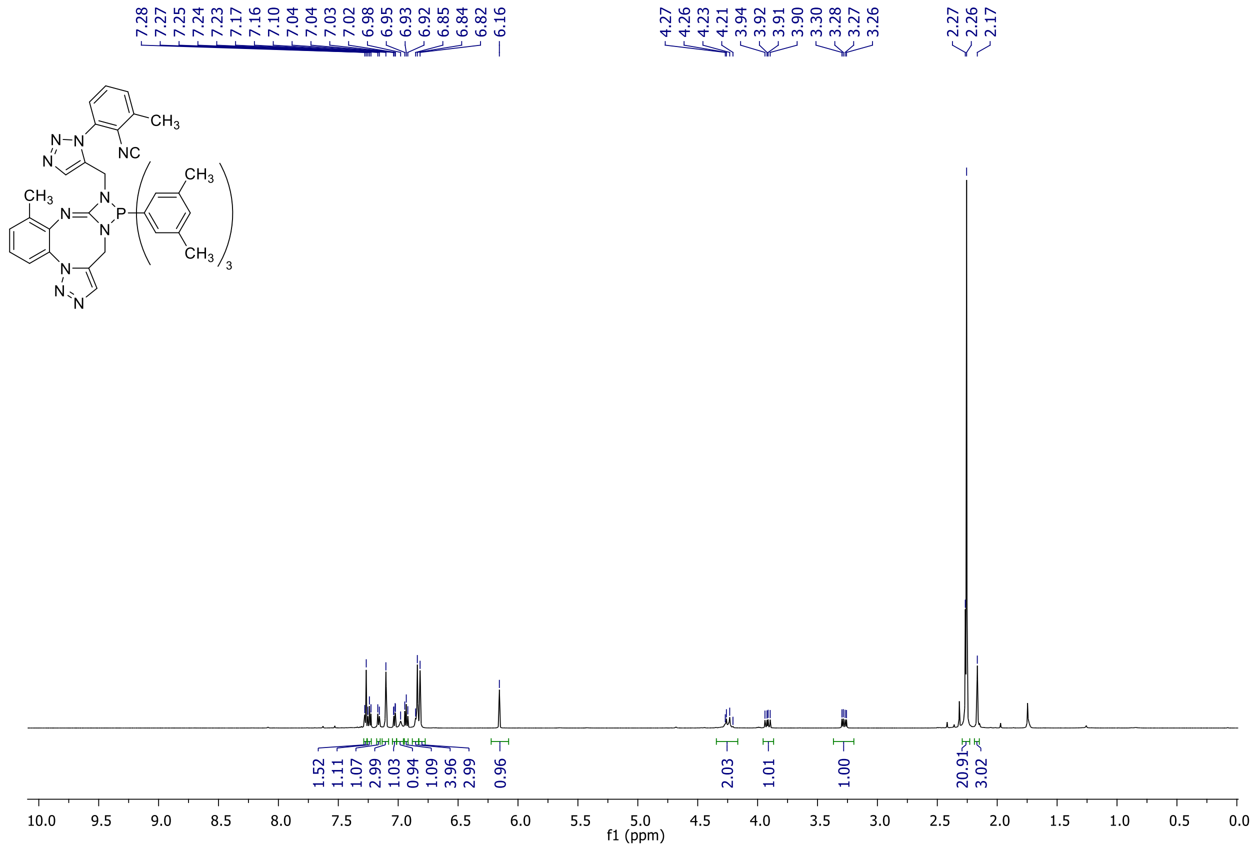

9cd (^{1}H{^{31}P} NMR, 600 MHz, $CDCl_3$, 298 K)

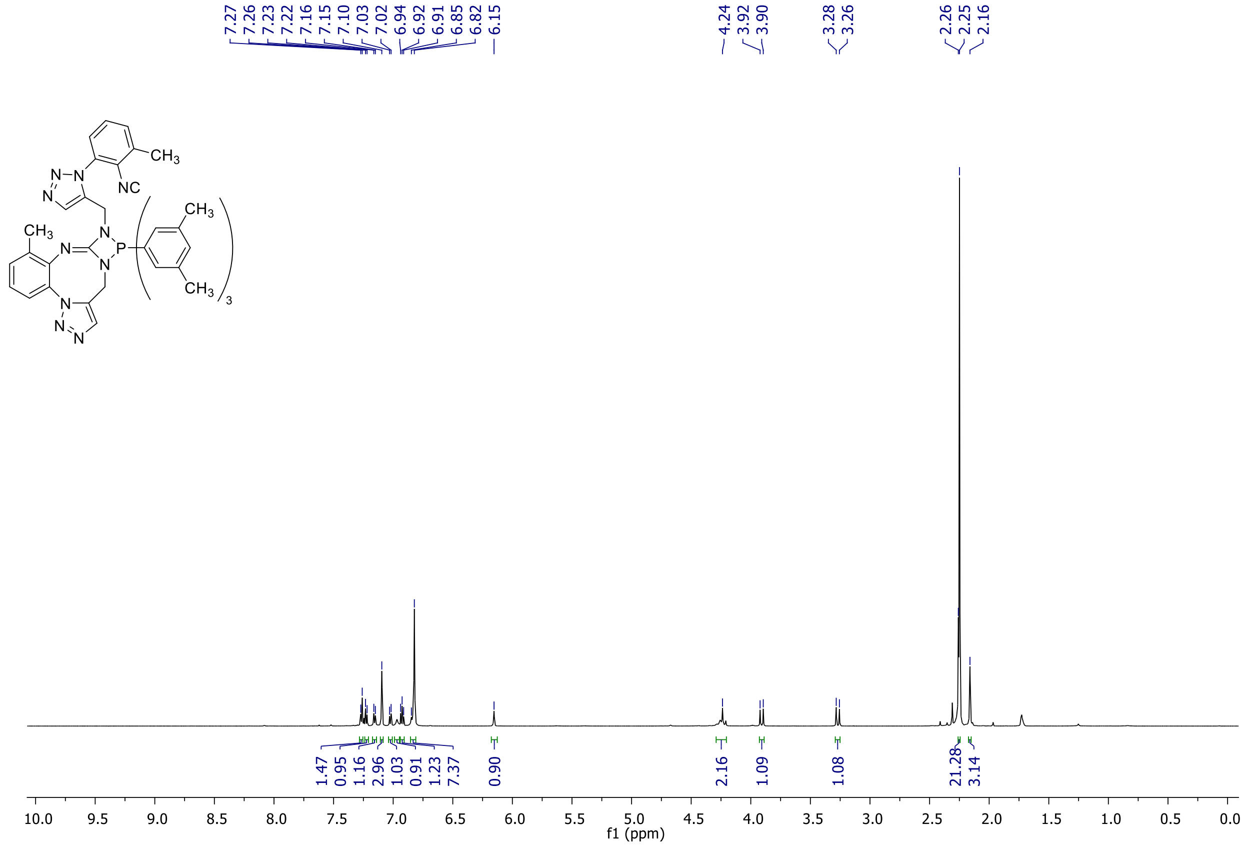

9cd (^{13}C NMR, 150 MHz, $CDCl_3$, 298 K)

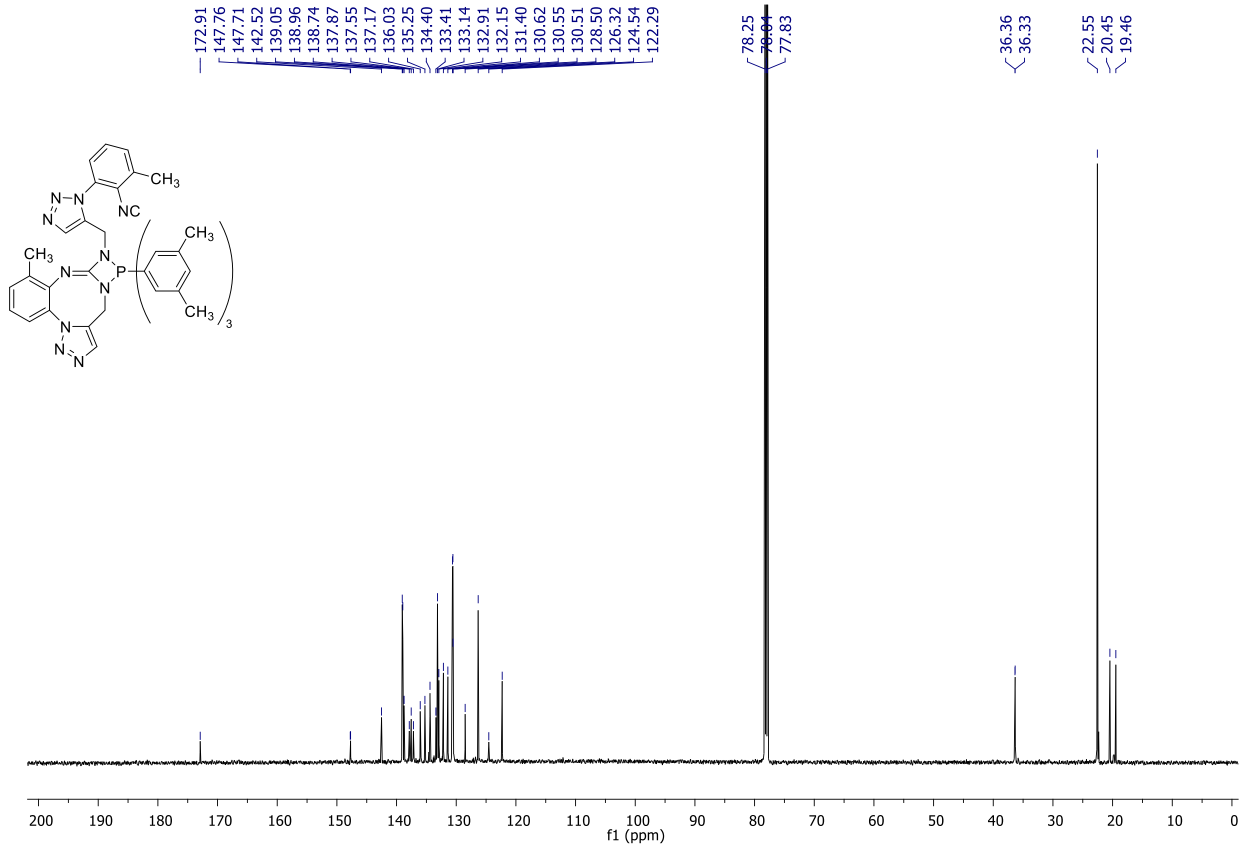

9cd (DEPT-135 NMR, 150 MHz, $CDCl_3$, 298 K)

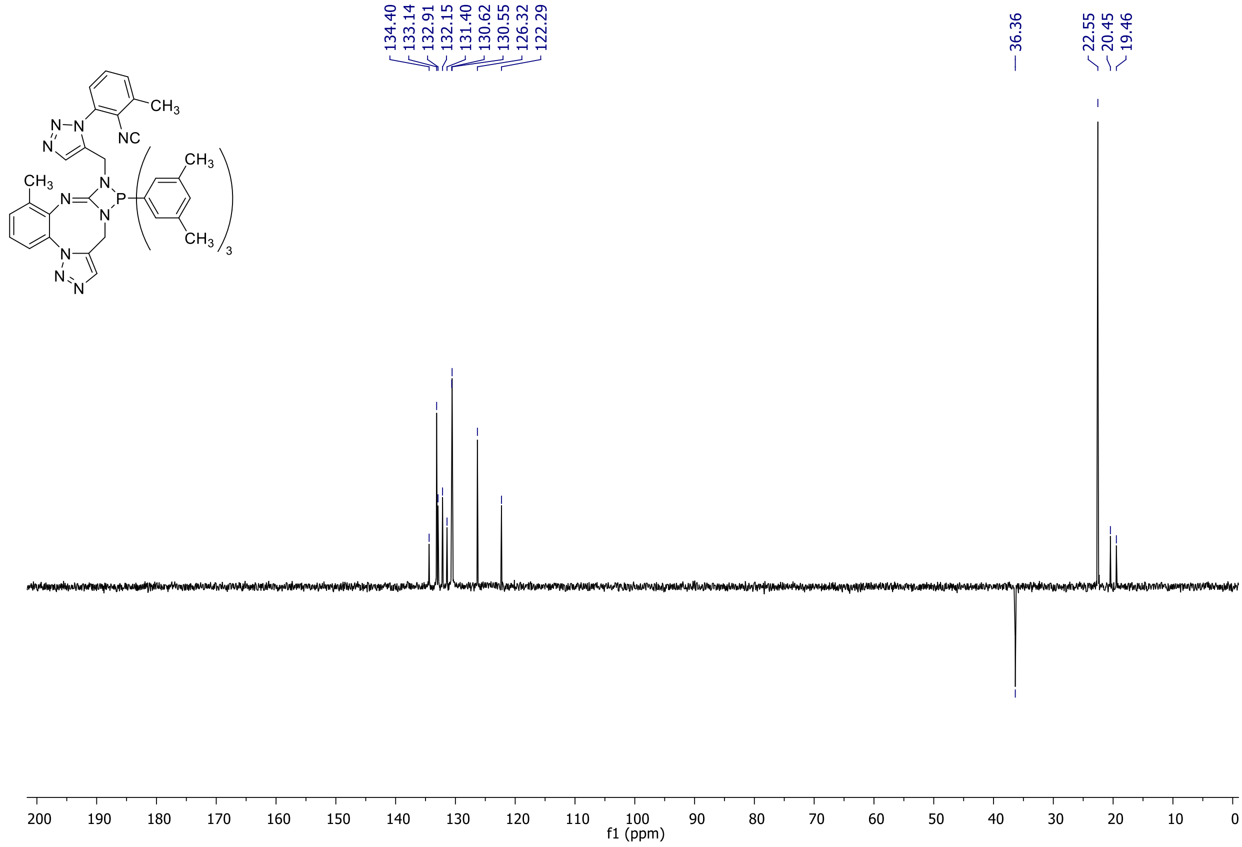

9cd (^{31}P NMR, 243 MHz, $CDCl_3$, 298 K)

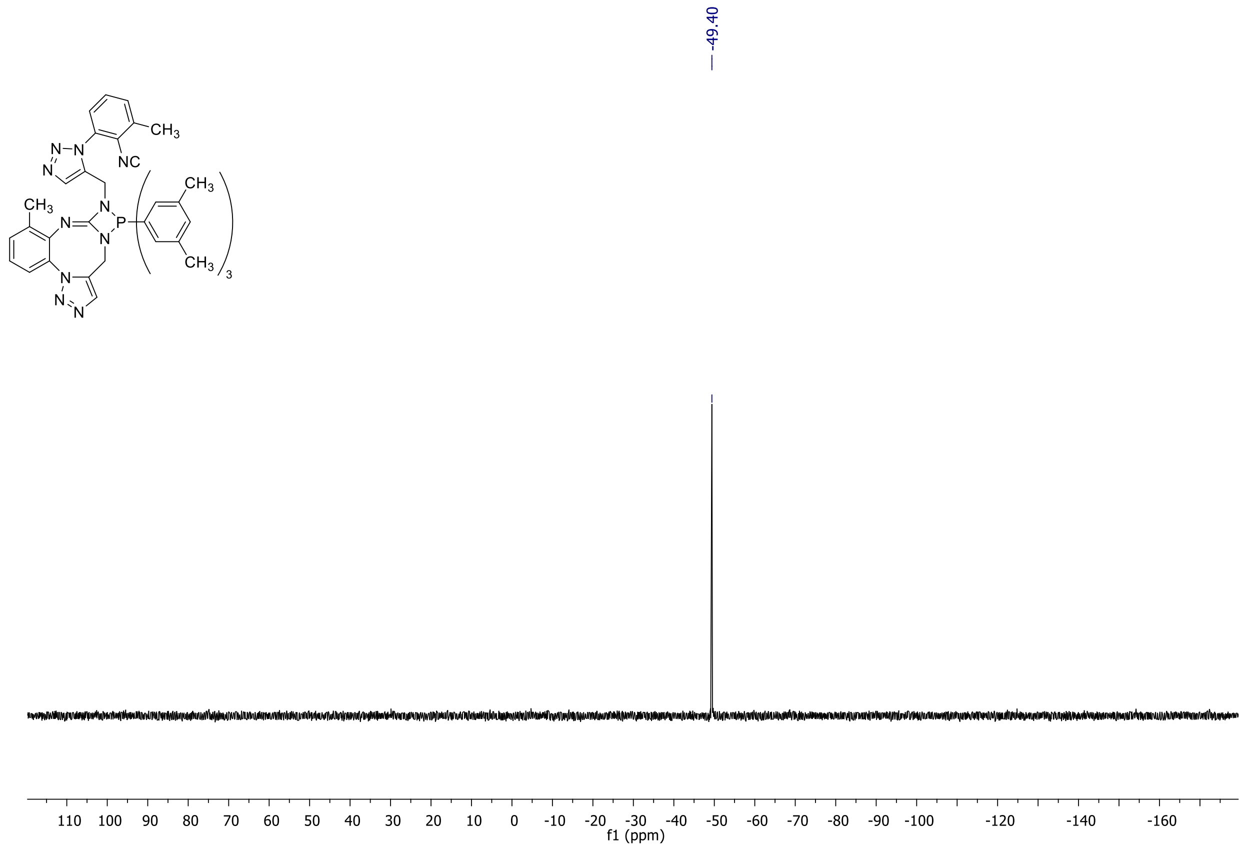

9da (^{1}H NMR, 400 MHz, $CDCl_3$, 298 K)

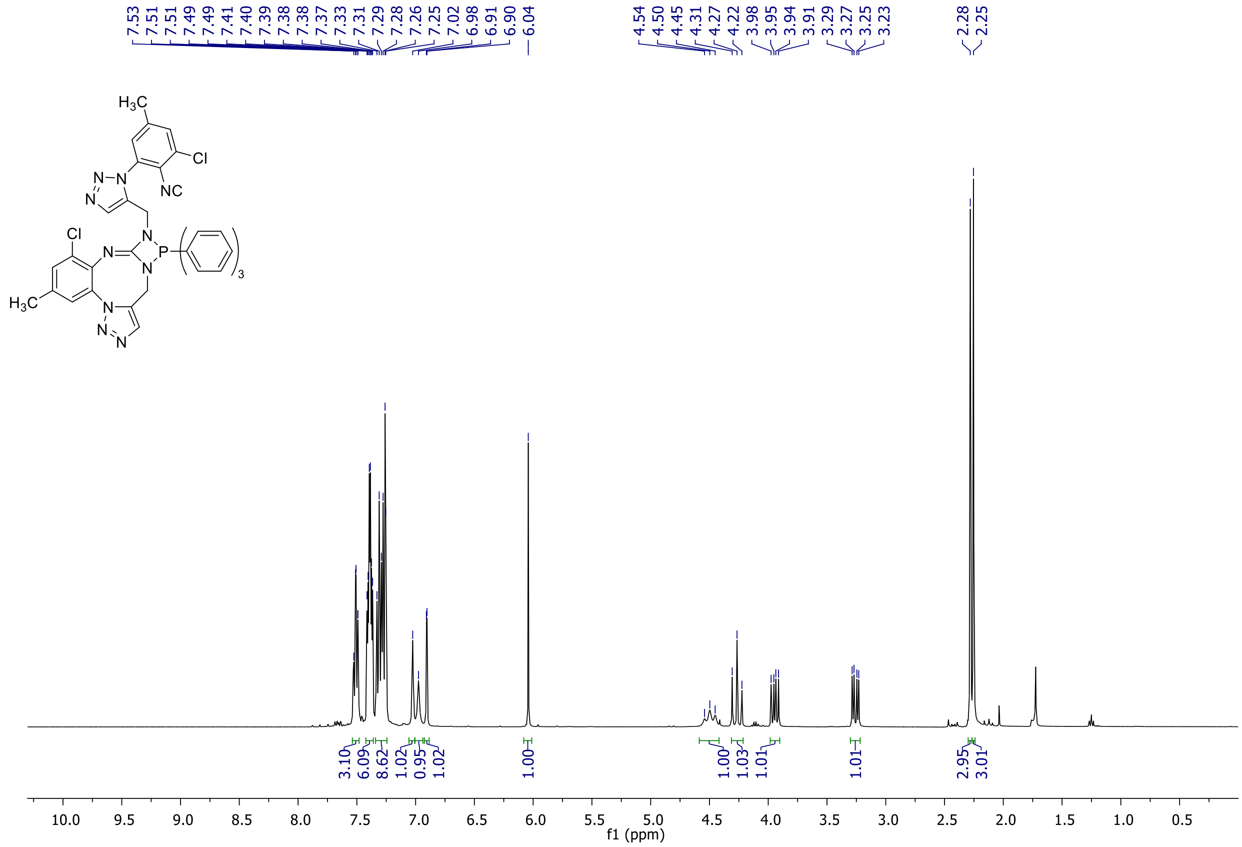

9da ($^{1}H\{^{31}P\}$ NMR, 400 MHz, $CDCl_3$, 298 K)

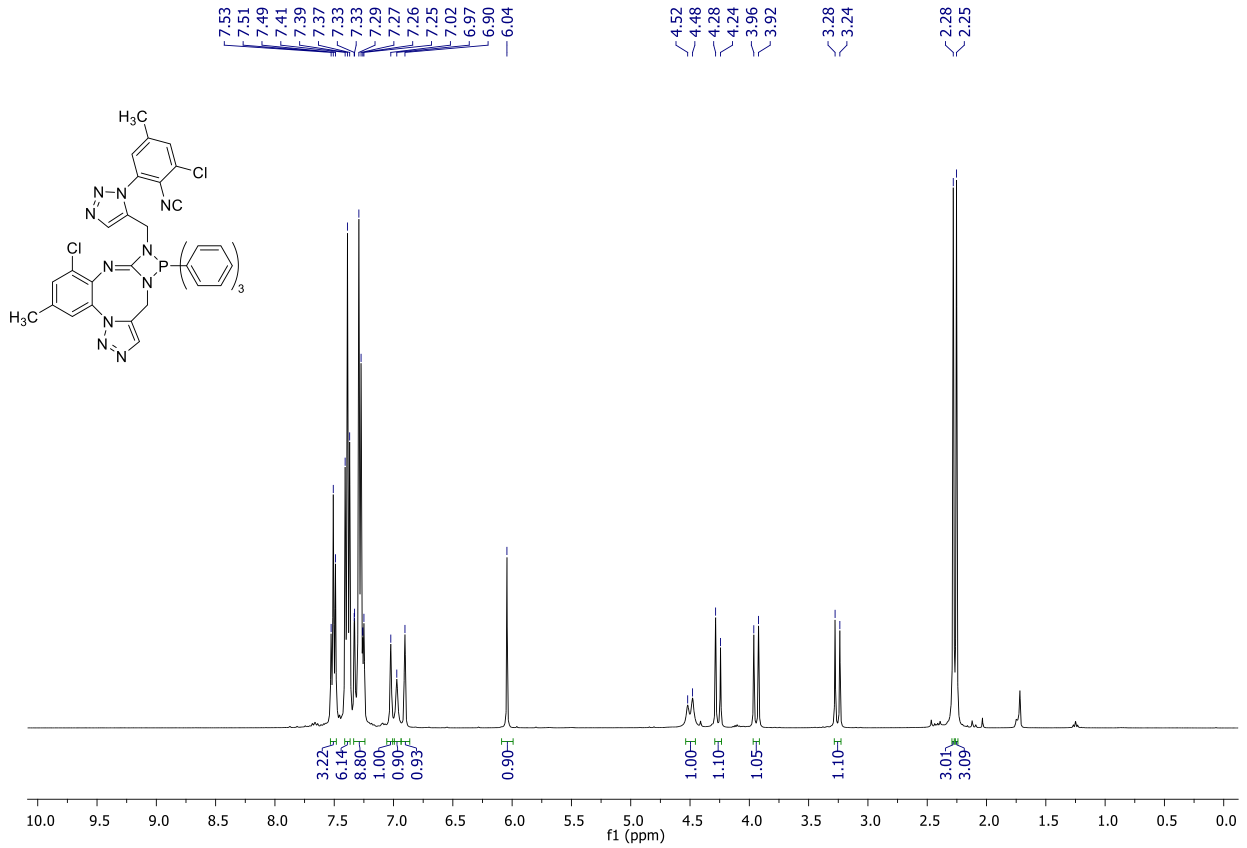

9da ($^{1}H^{1}H$-COSY NMR, 400 MHz, $CDCl_3$, 298 K)

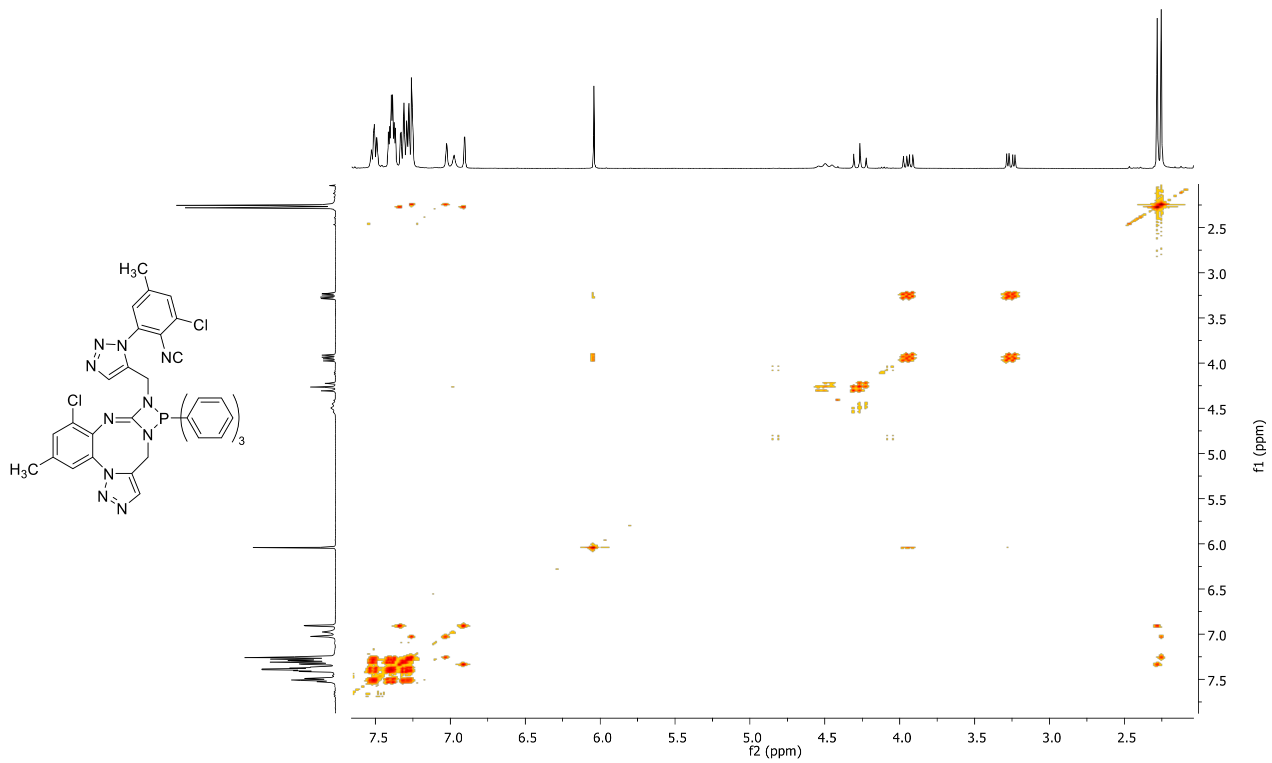

9da (^{13}C NMR, 100 MHz, $CDCl_3$, 298 K).

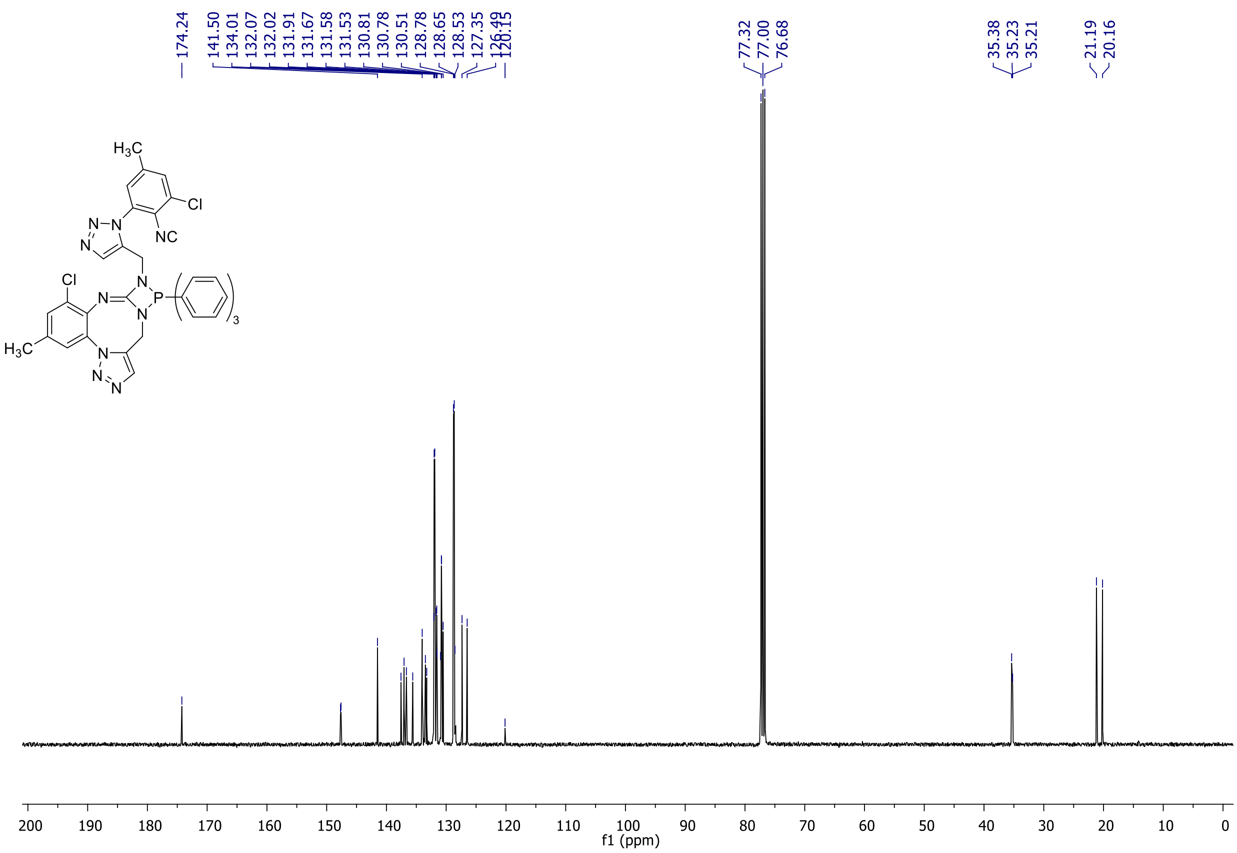

9da (DEPT-135 NMR, 100 MHz, $CDCl_3$, 298 K).

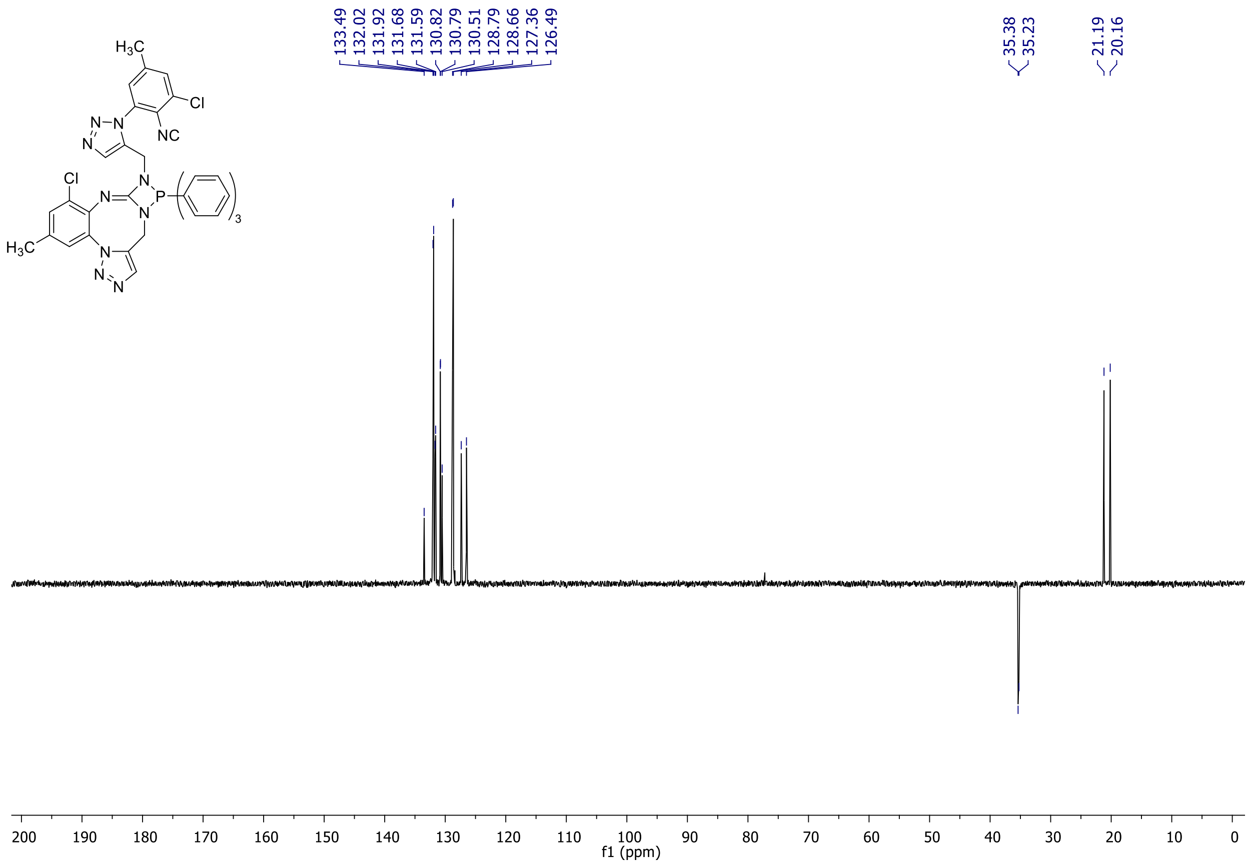

9da (HMQC NMR, 400 MHz, $CDCl_3$, 298 K)

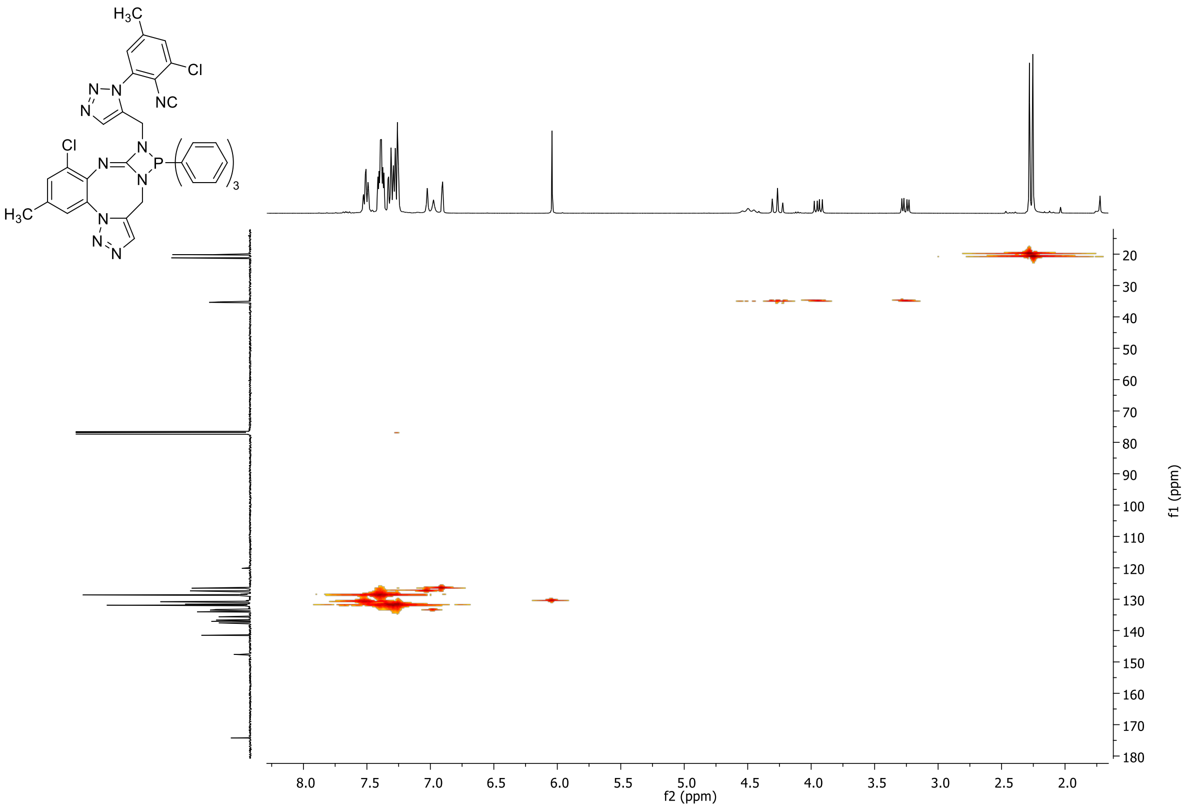

9da (^{31}P NMR, 121.5 MHz, $CDCl_3$, 298 K)

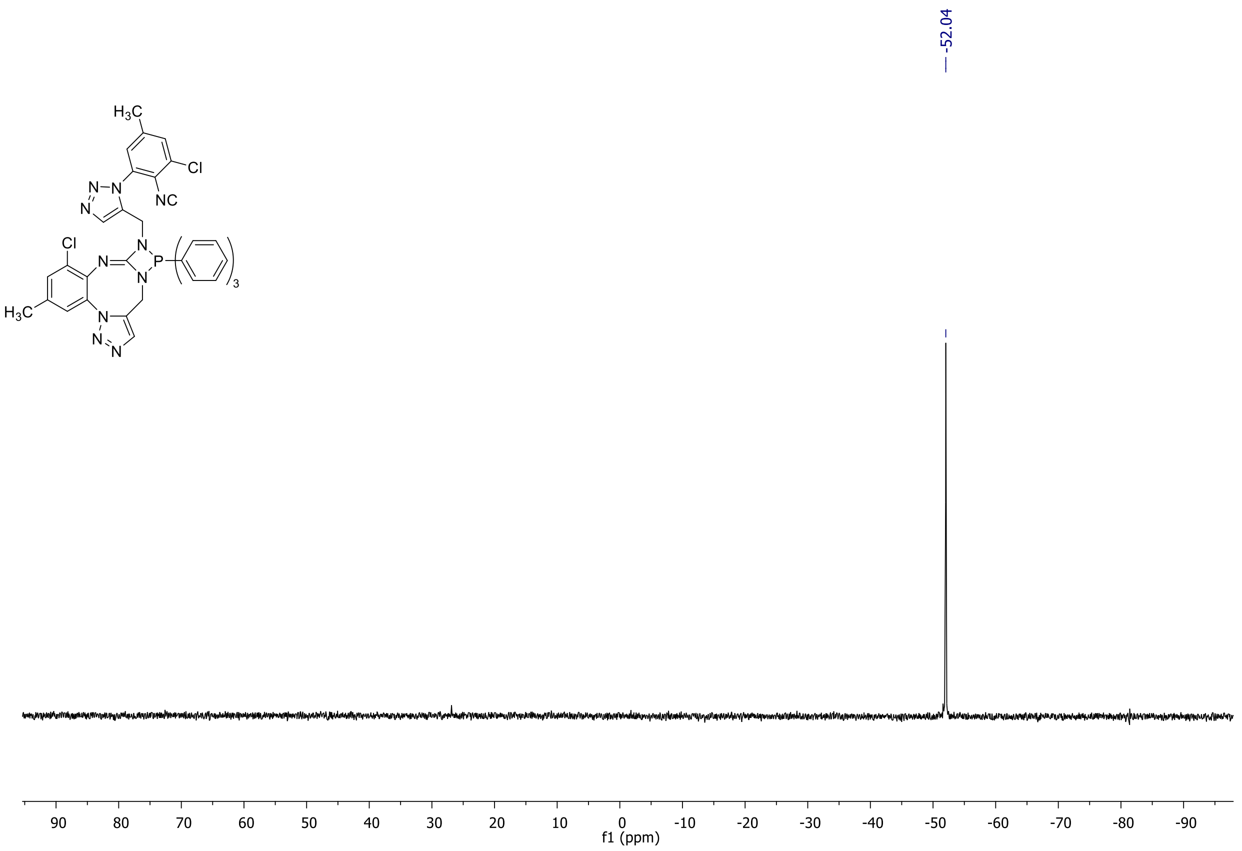

9db (^{1}H NMR, 400 MHz, $CDCl_3$, 298 K)

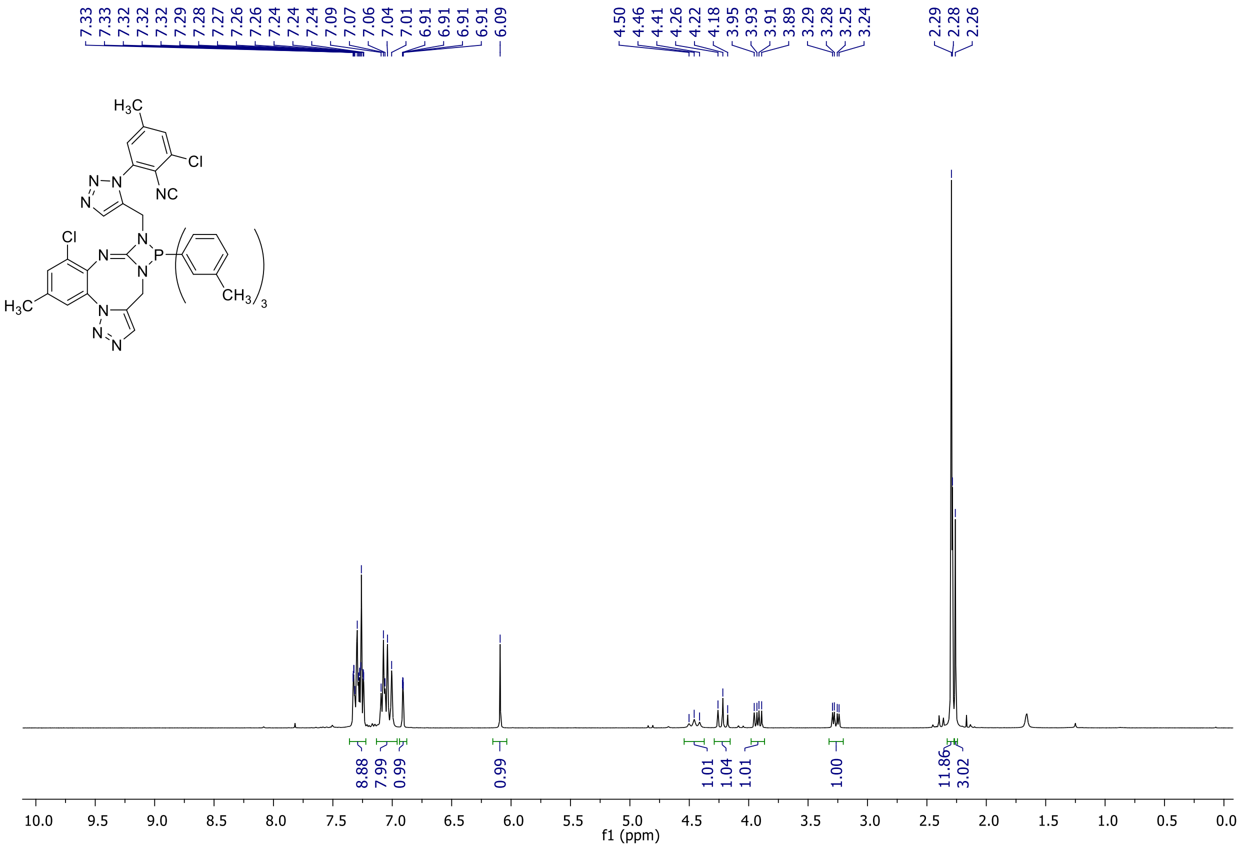

9db (^{13}C NMR, 100 MHz, $CDCl_3$, 298 K)

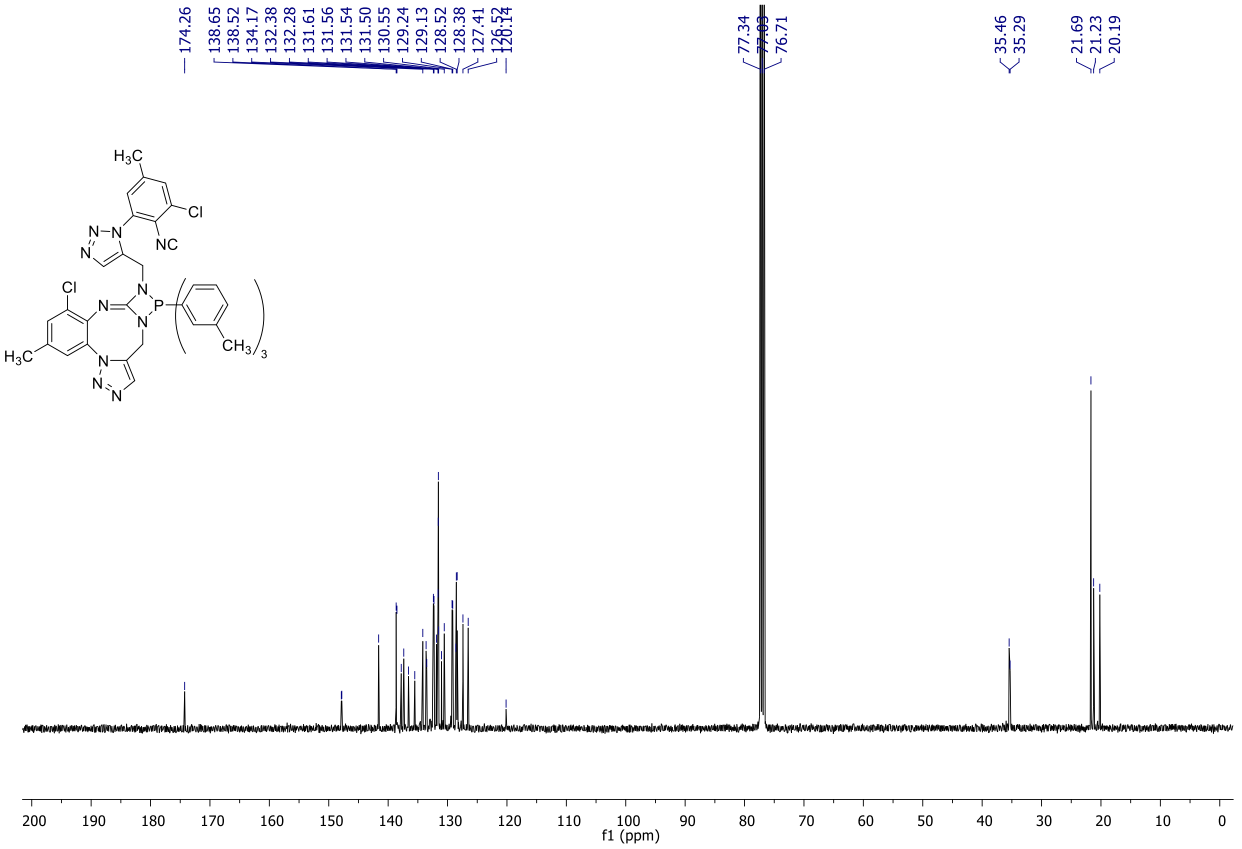

9db (DEPT-135 NMR, 100 MHz, $CDCl_3$, 298 K)

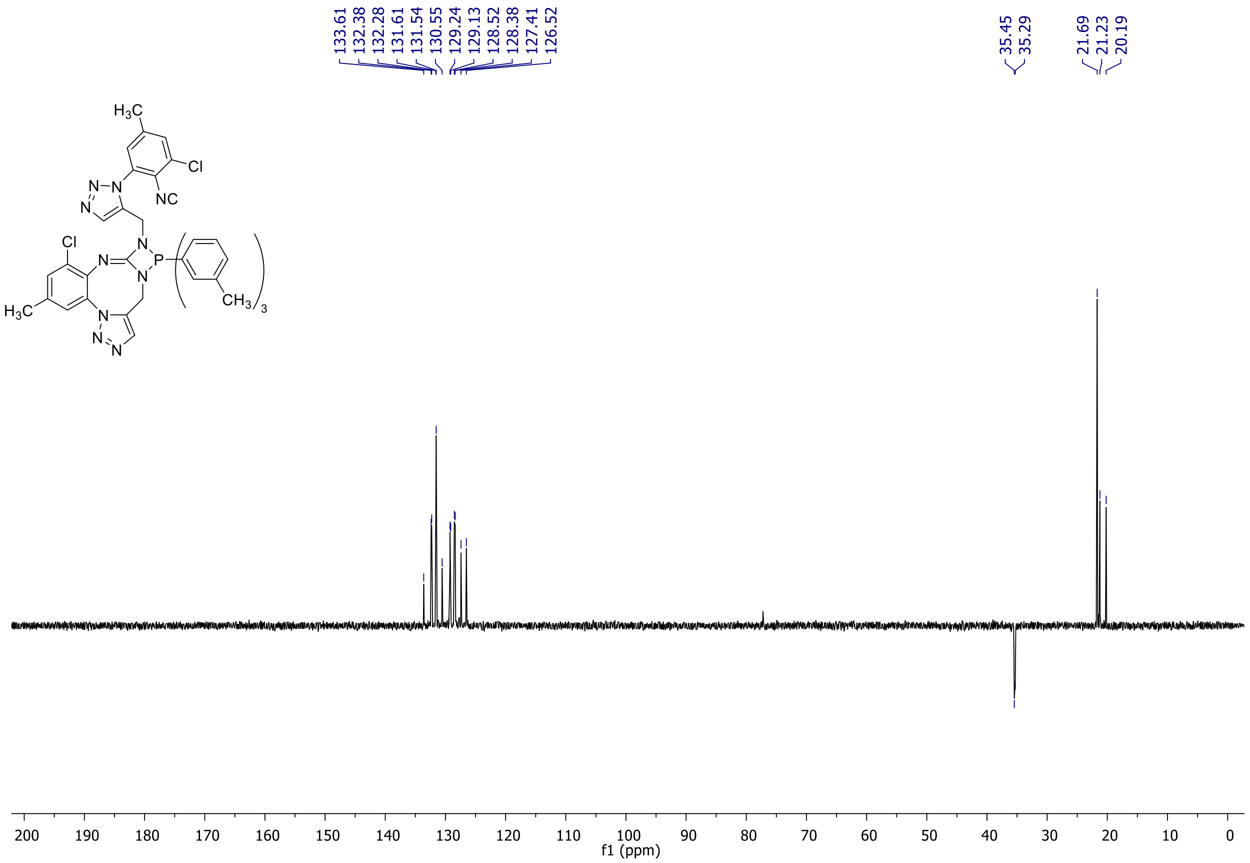

9db (^{31}P NMR, 162 MHz, $CDCl_3$, 298 K)

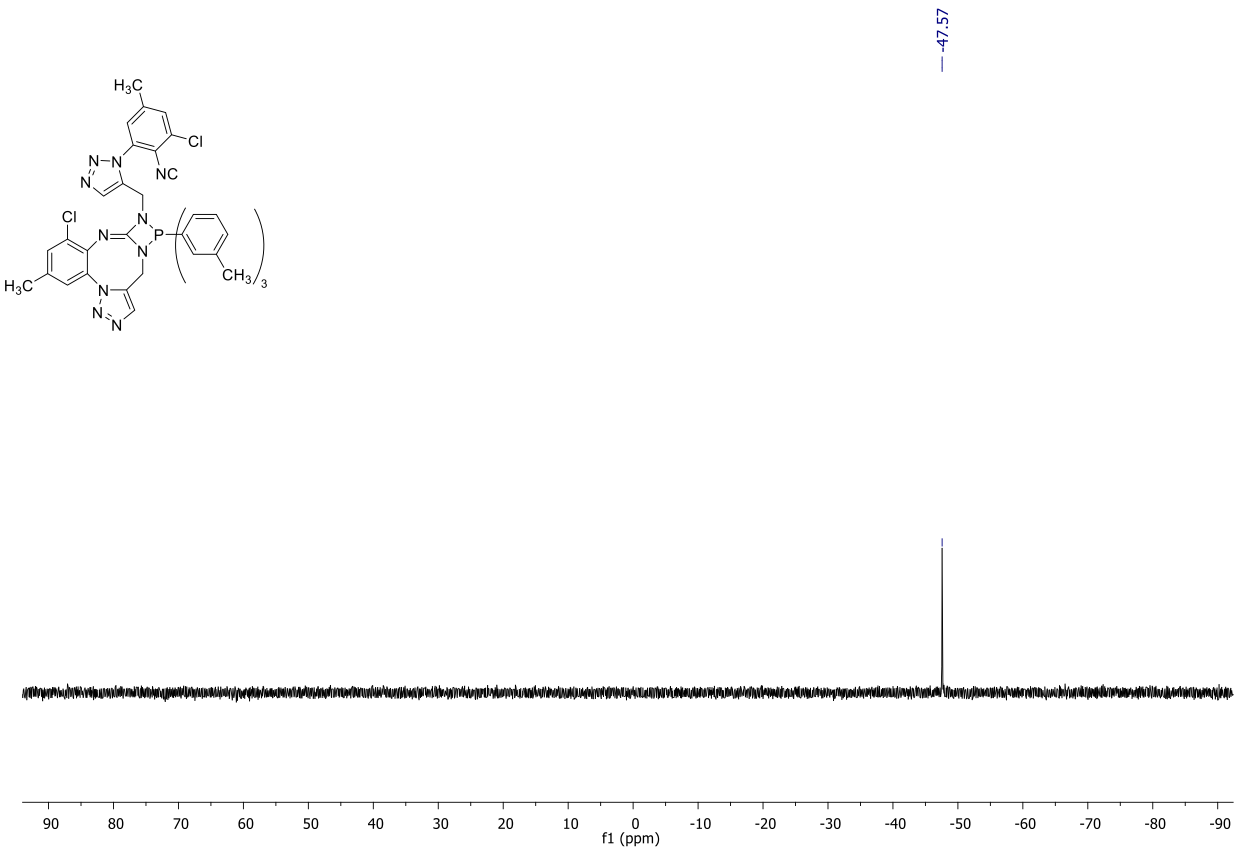

9dc (^{1}H NMR, 400 MHz, $CDCl_3$, 298 K)

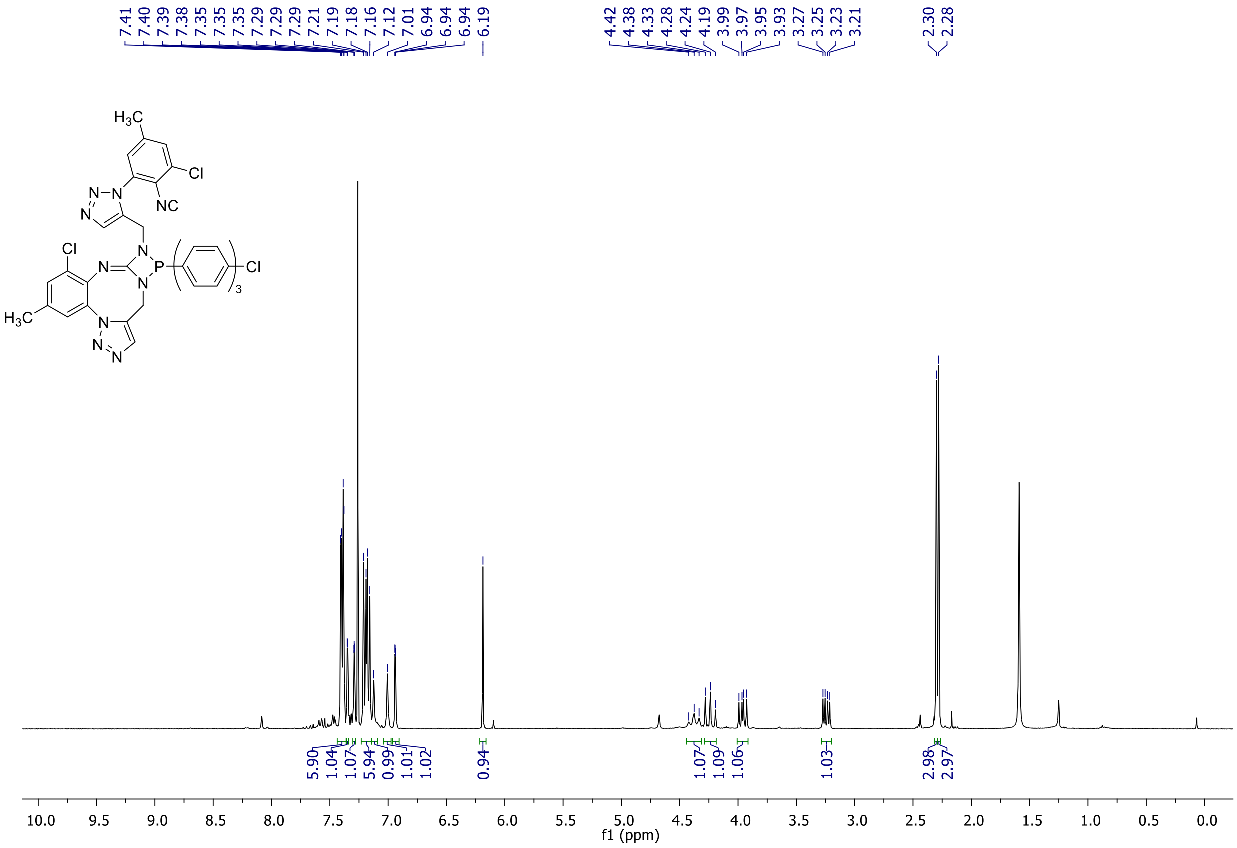

9dc (^{1}H{^{31}P} NMR, 400 MHz, $CDCl_3$, 298 K)

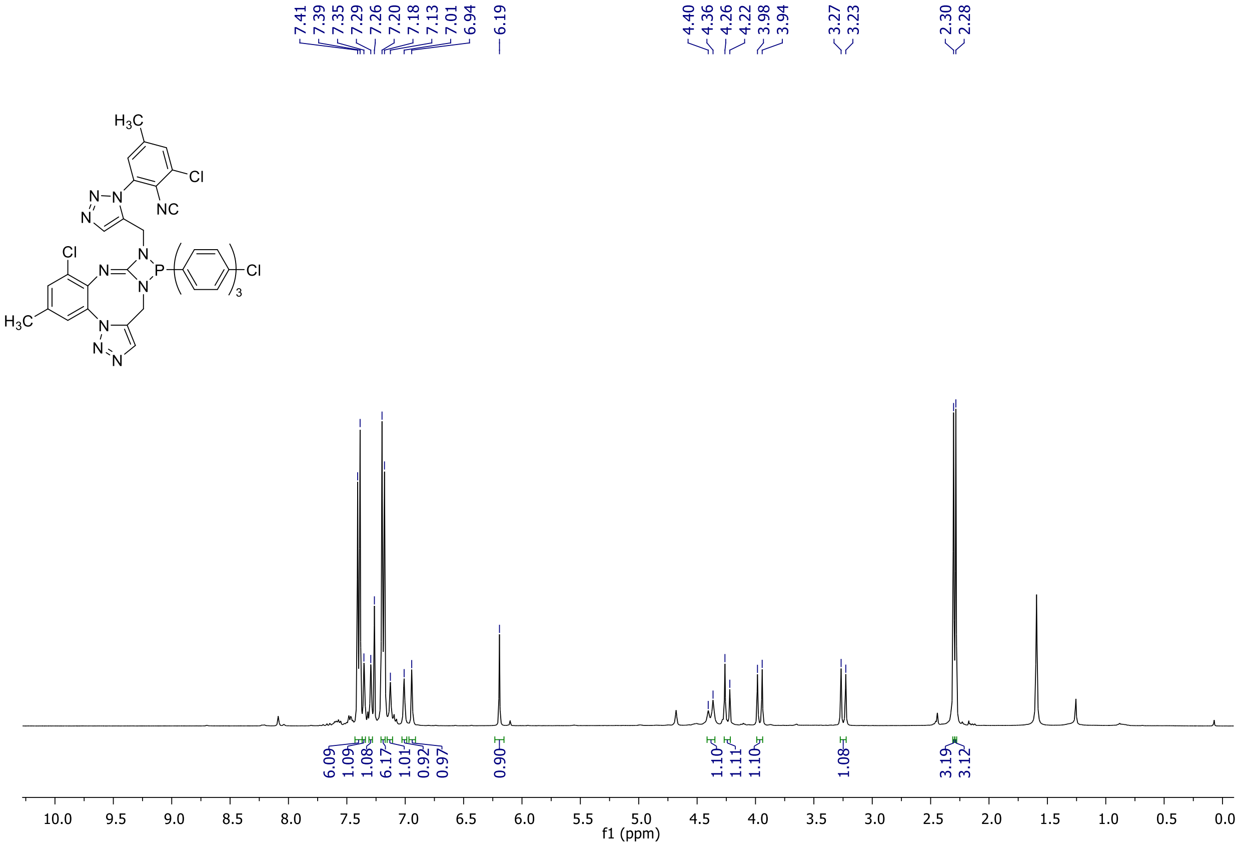

9dc (^{13}C NMR, 100 MHz, $CDCl_3$, 298 K)

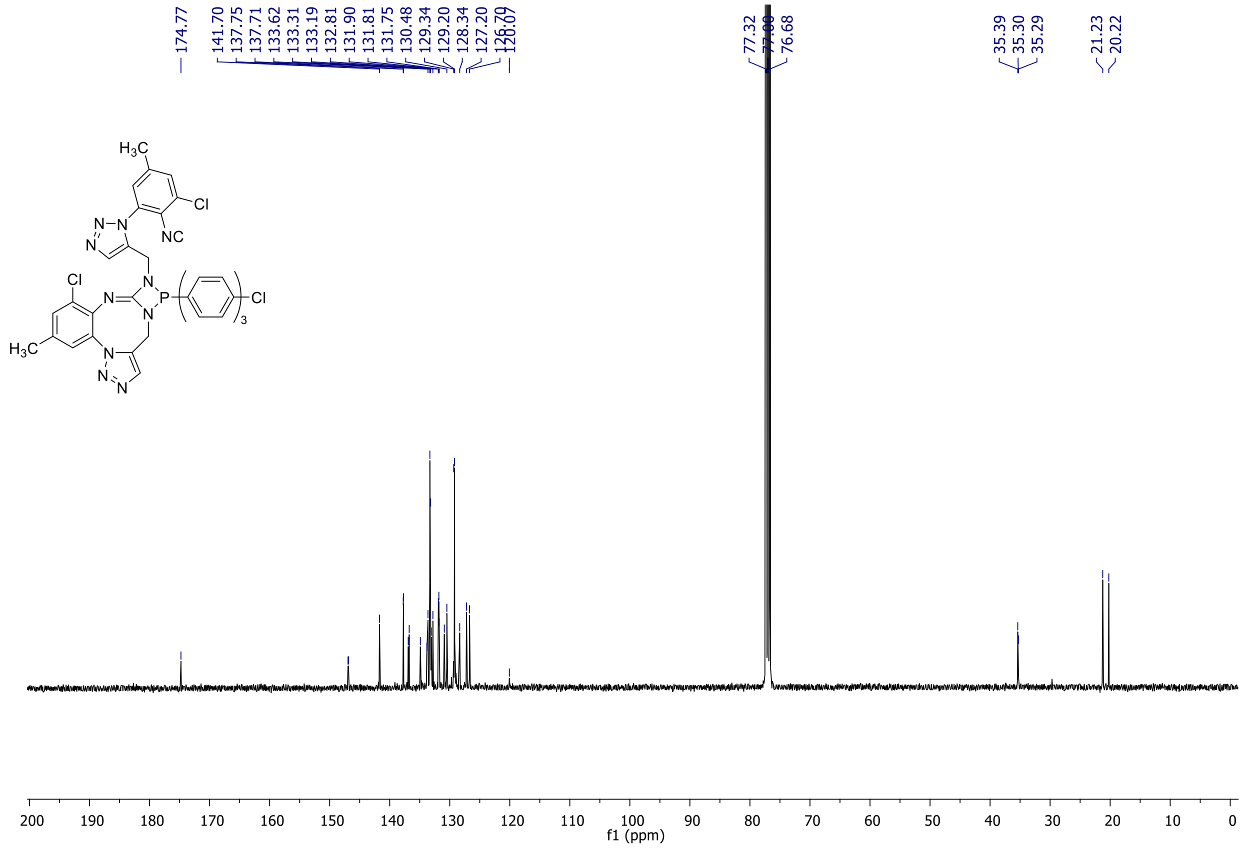

9dc (DEPT-135 NMR, 100 MHz, $CDCl_3$, 298 K)

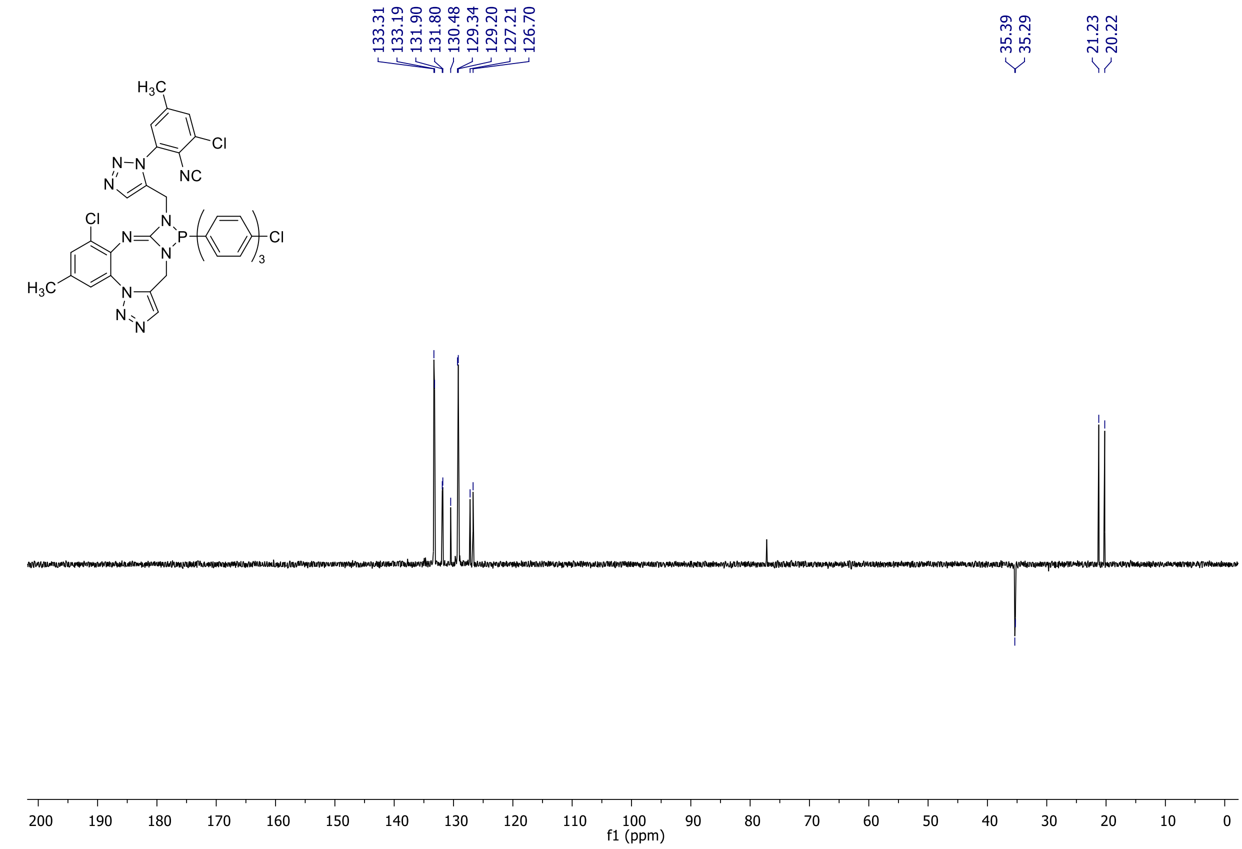

9dc (^{31}P NMR, 162.5 MHz, $CDCl_3$, 298 K)

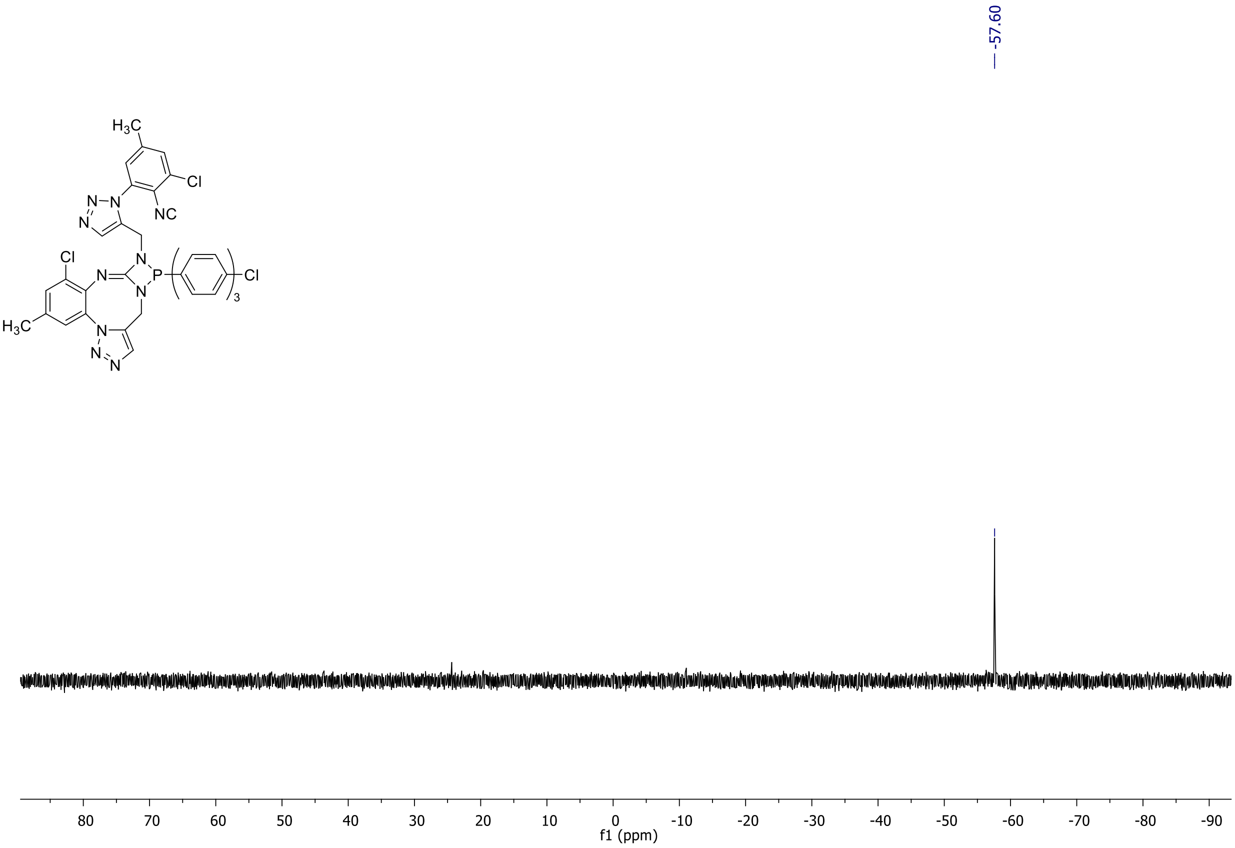

9dd (^{1}H NMR, 600 MHz, $CDCl_3$, 298 K)

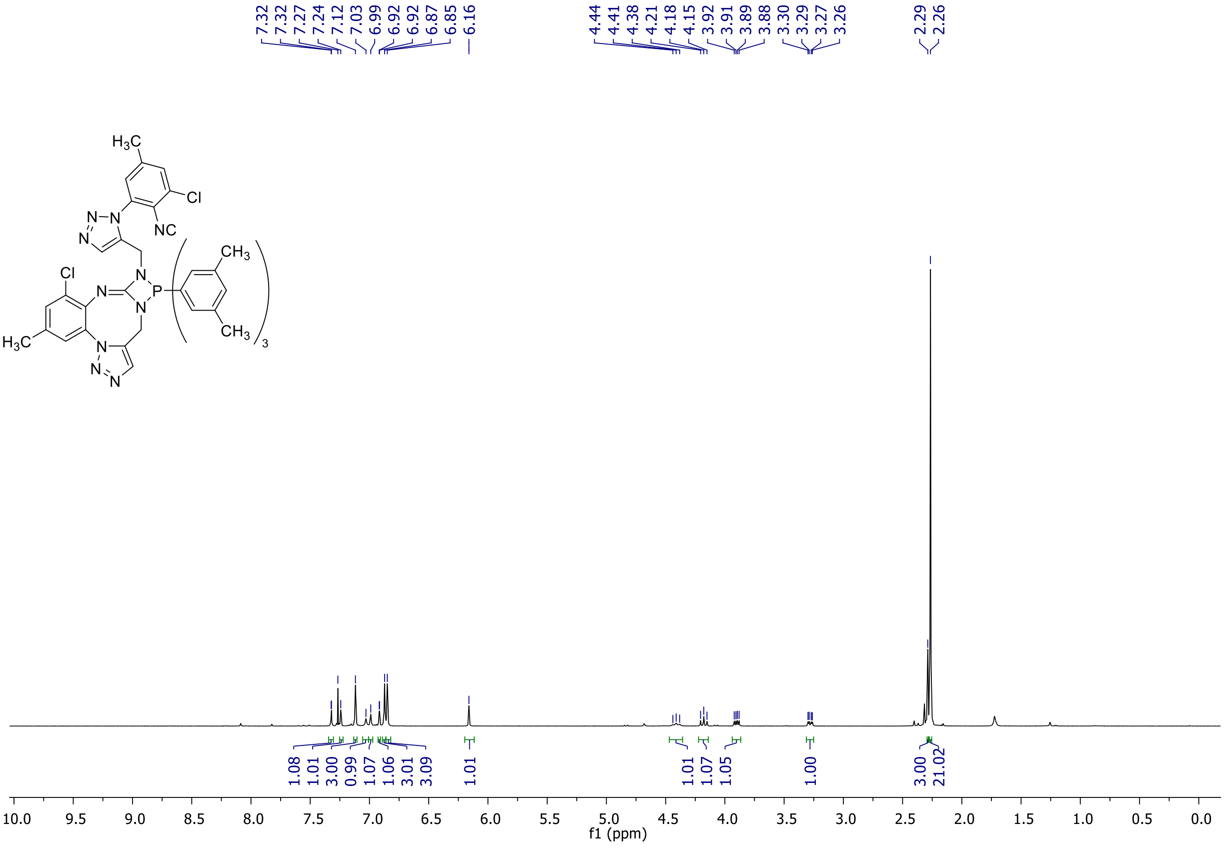

9dd ($^{1}H\{^{31}P\}$ NMR, 600 MHz, $CDCl_3$, 298 K)

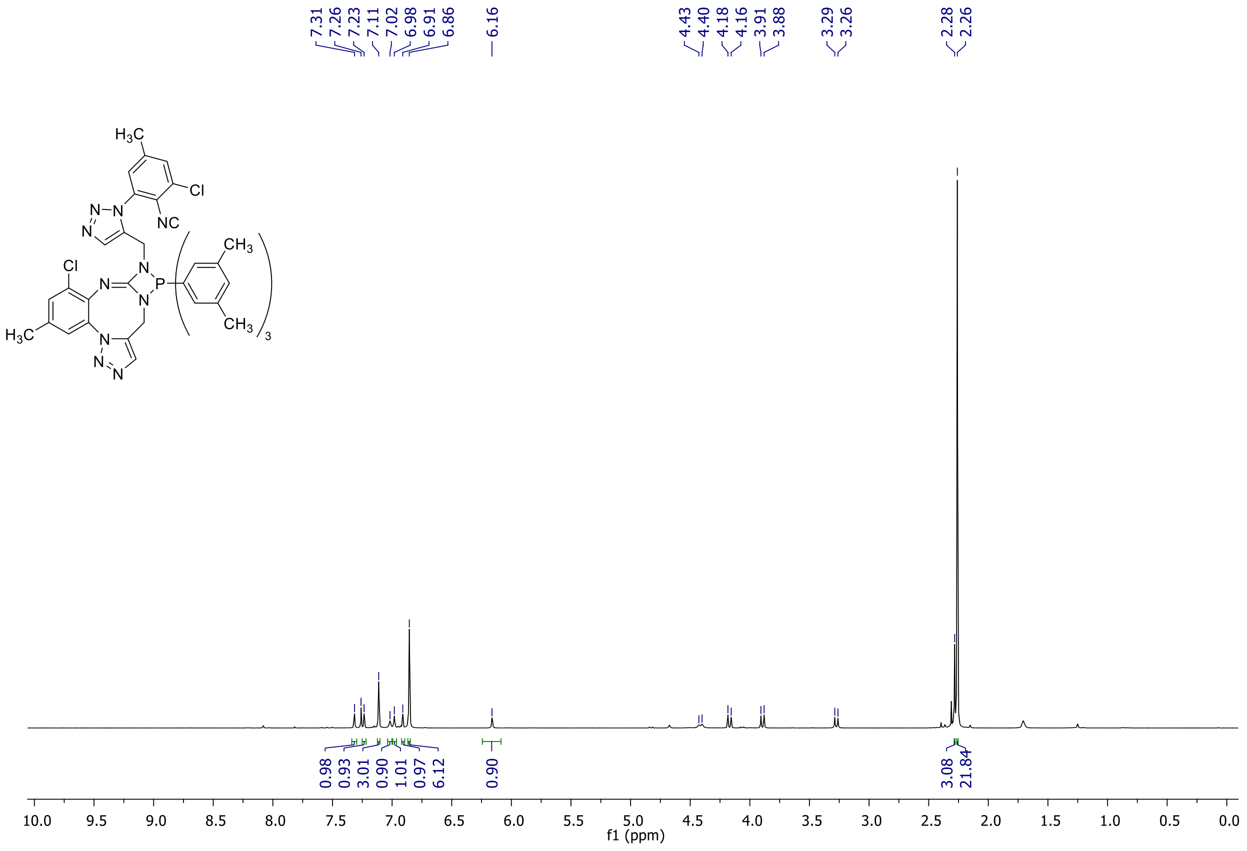

9dd (^{13}C NMR, 150 MHz, $CDCl_3$, 298 K)

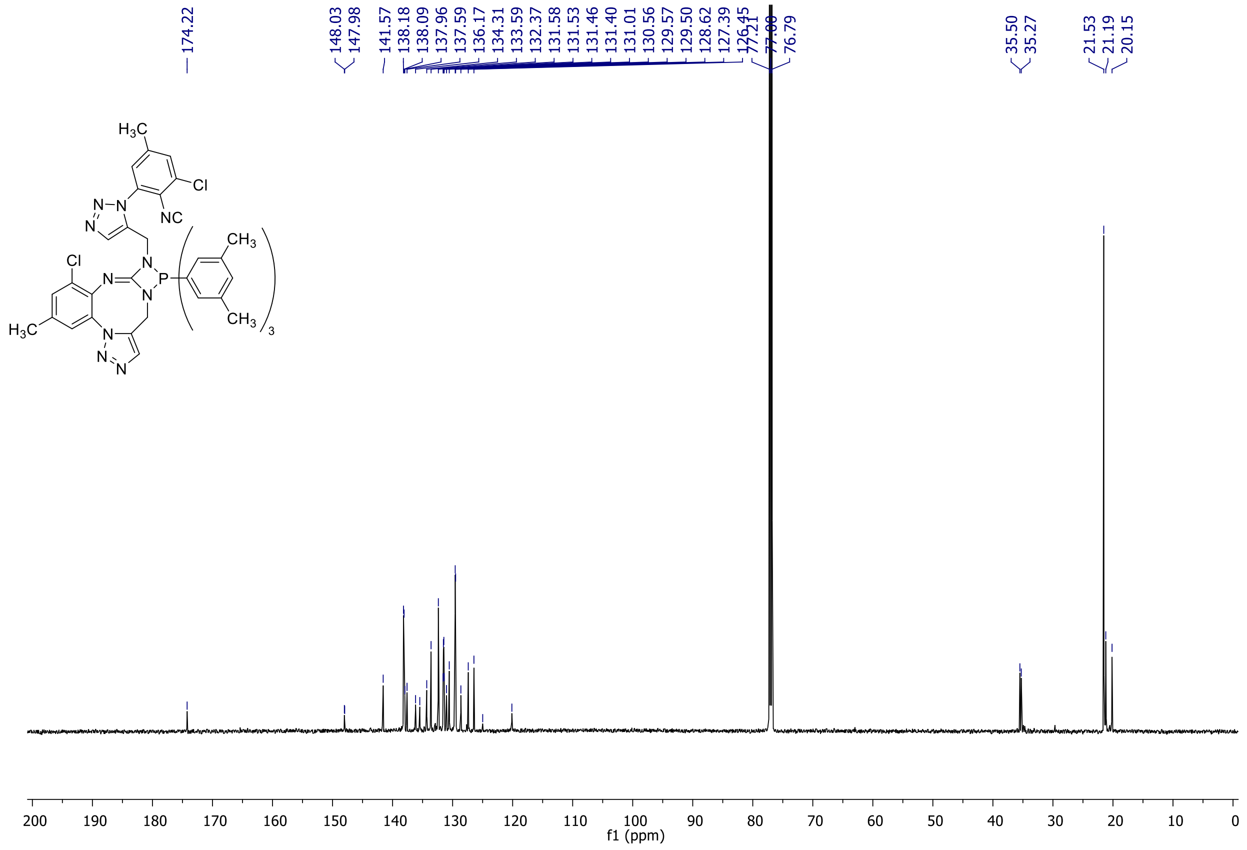

9dd (DEPT-135 NMR, 150 MHz, $CDCl_3$, 298 K)

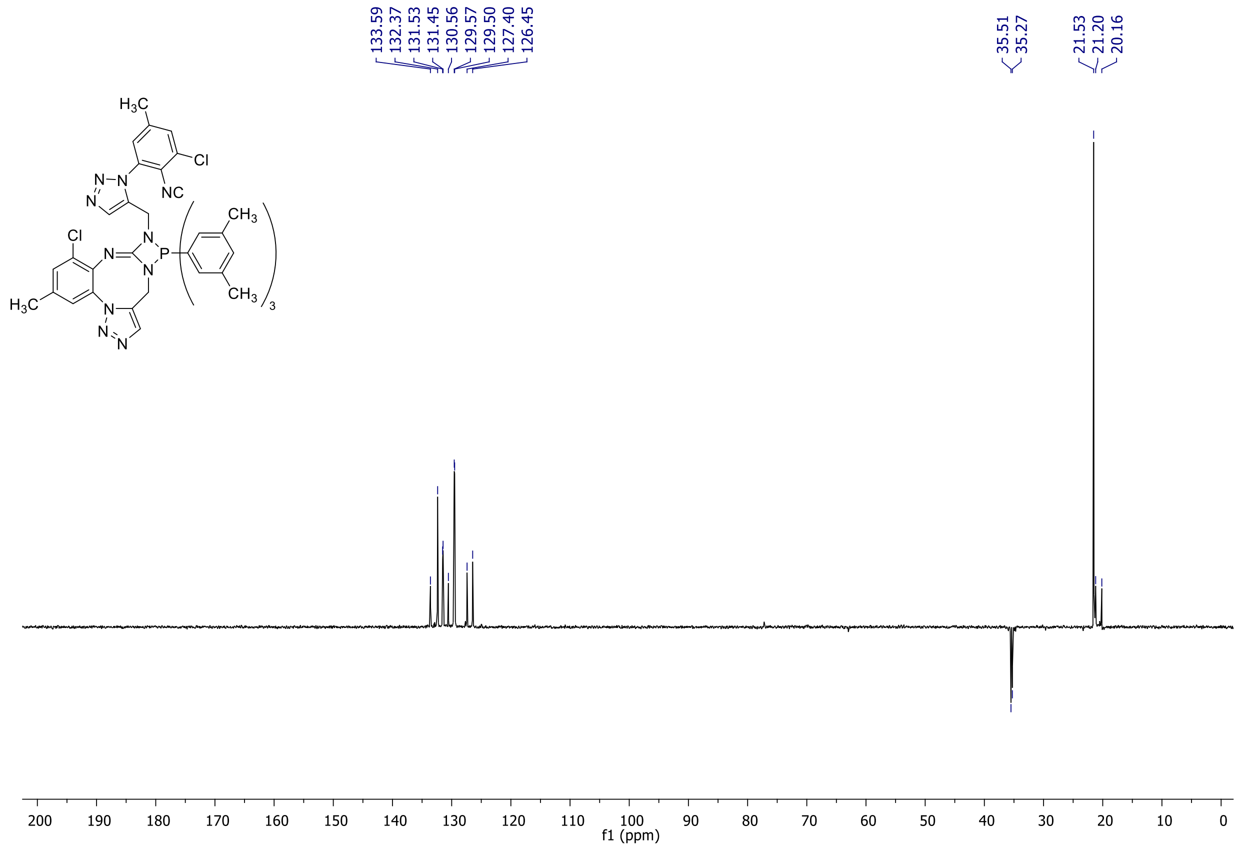

9dd (^{31}P NMR, 243 MHz, $CDCl_3$, 298 K)

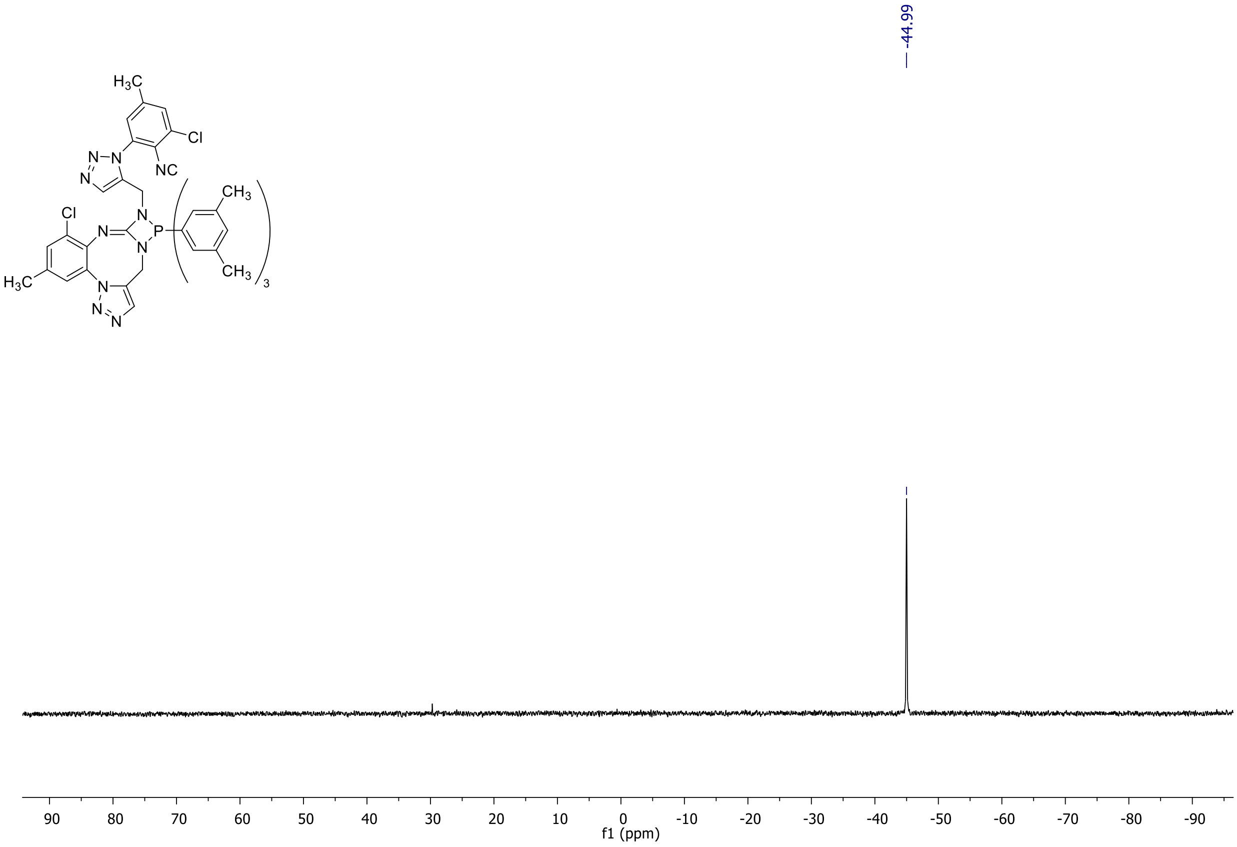

9de (^{1}H NMR, 600 MHz, $CDCl_3$, 298 K)

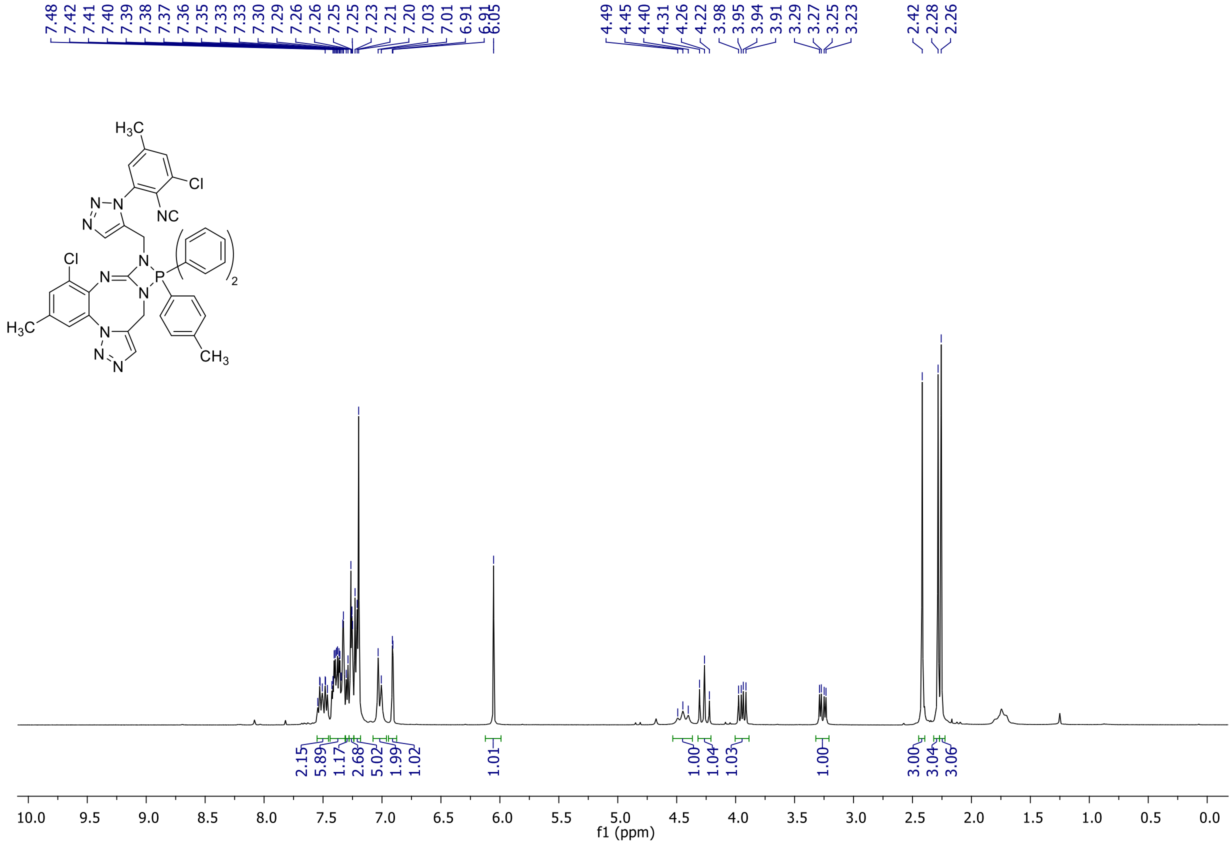

9de (^{1}H{^{31}P} NMR, 600 MHz, $CDCl_3$, 298 K)

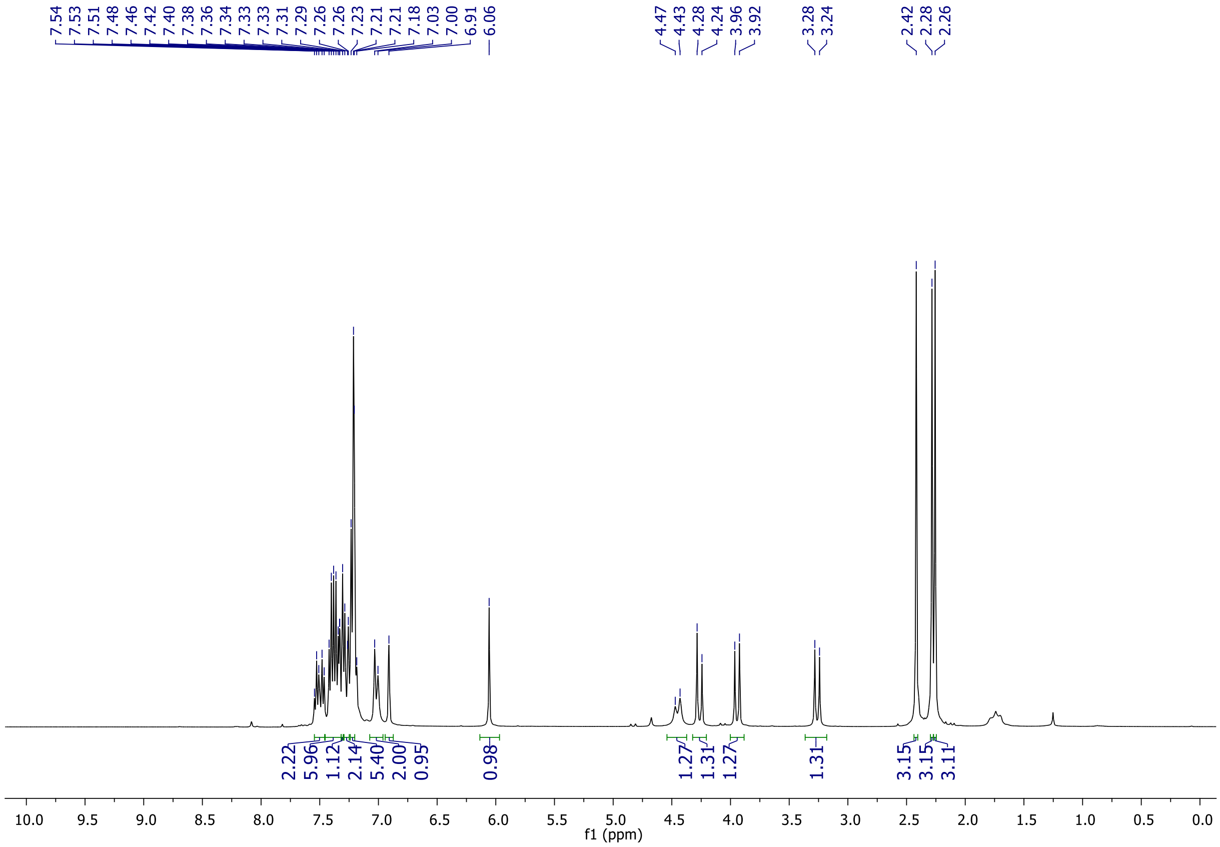

9de (^{13}C NMR, 150 MHz, $CDCl_3$, 298 K)

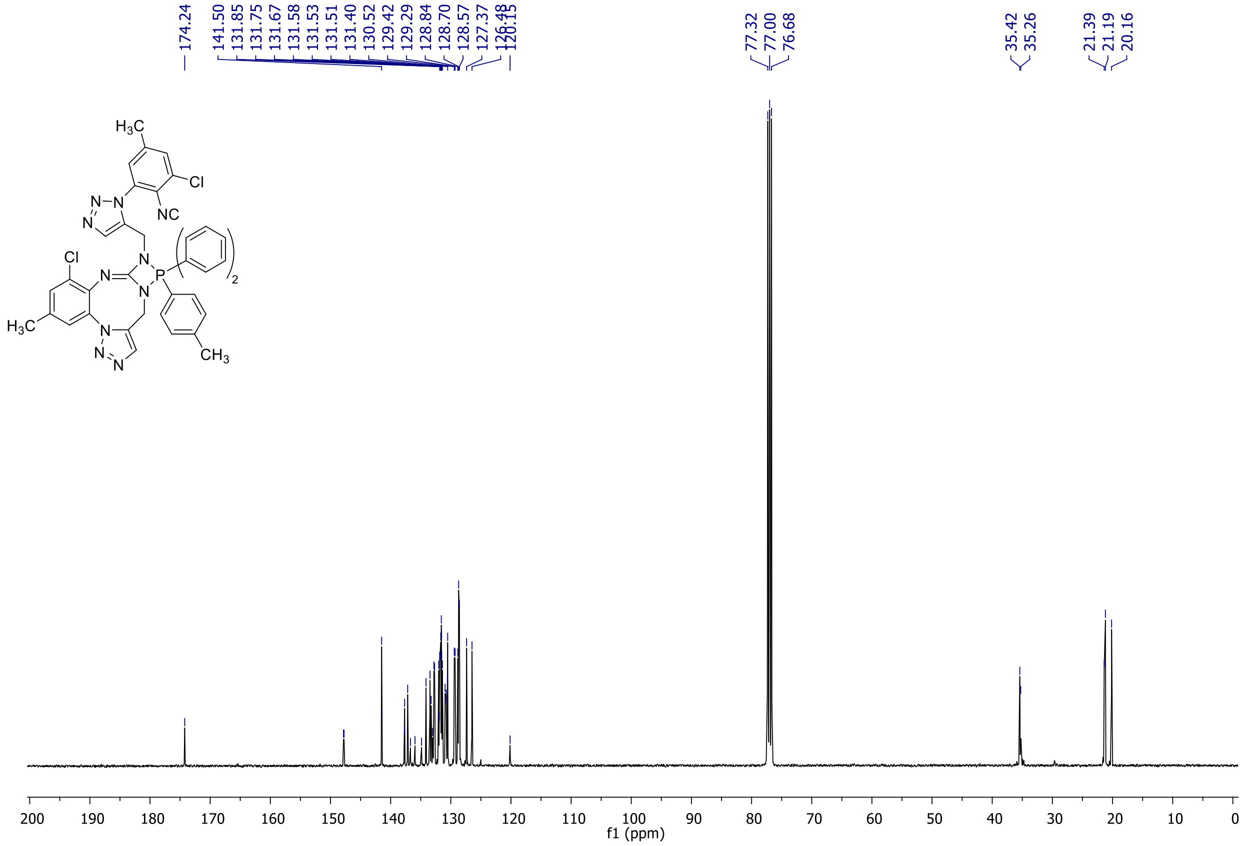

9de (DEPT-135 NMR, 150 MHz, $CDCl_3$, 298 K)

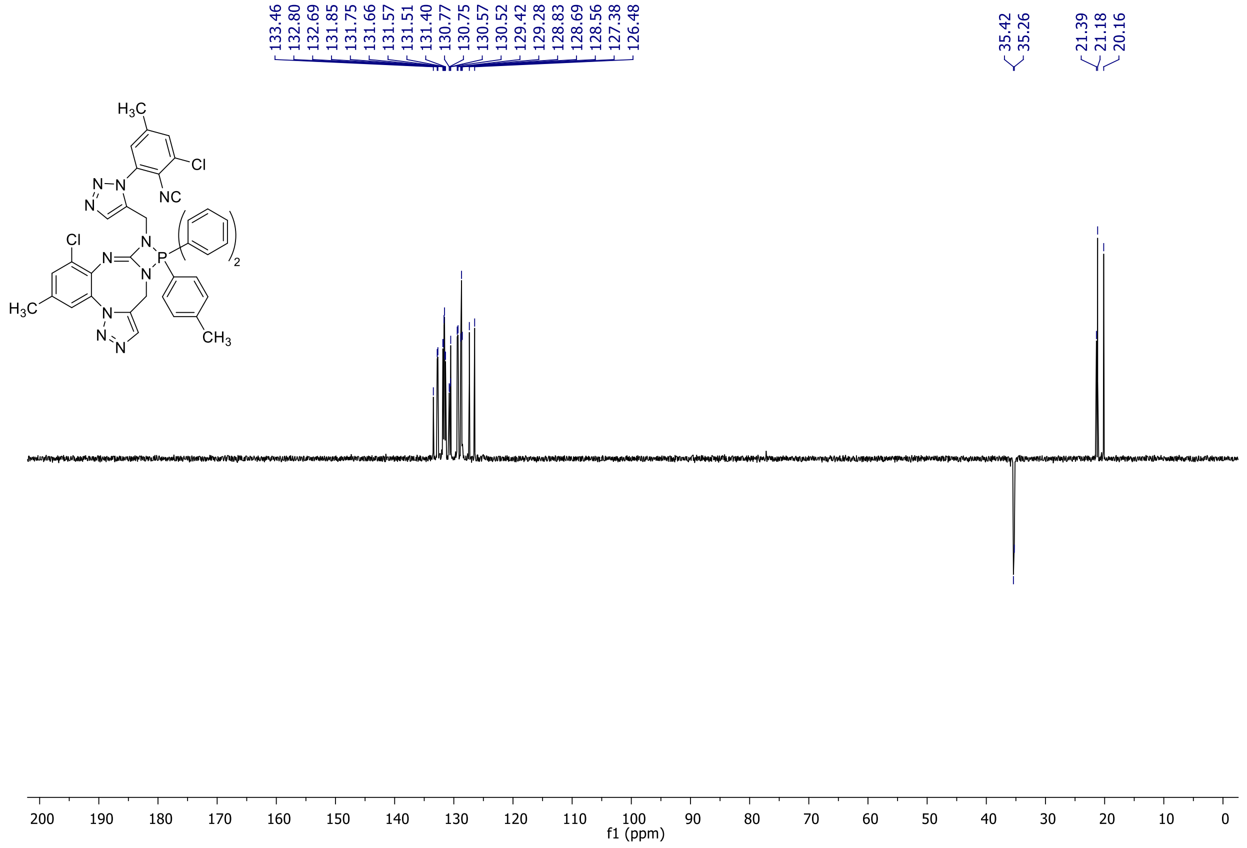

9de (^{31}P NMR, 243 MHz, $CDCl_3$, 298 K)

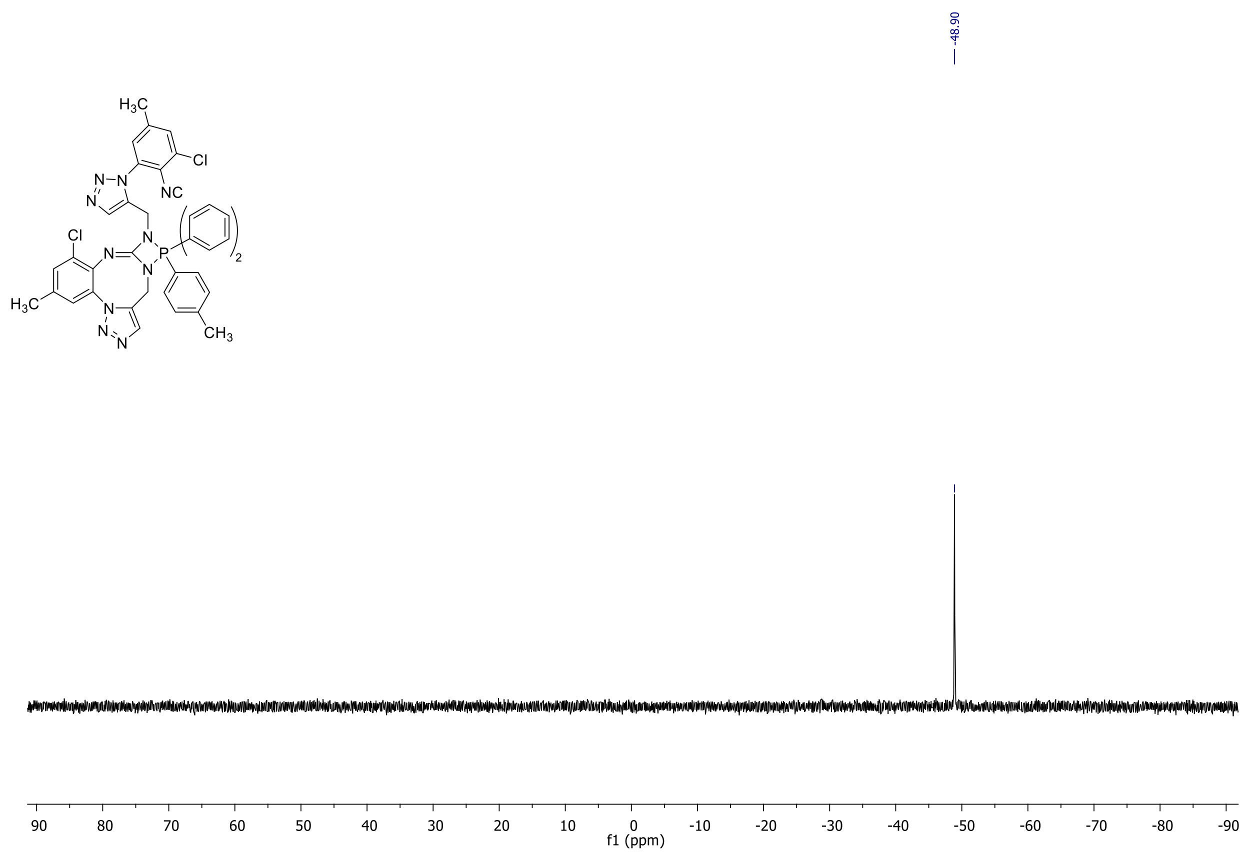

13b (^{1}H NMR, 600 MHz, $CDCl_3$, 298 K)

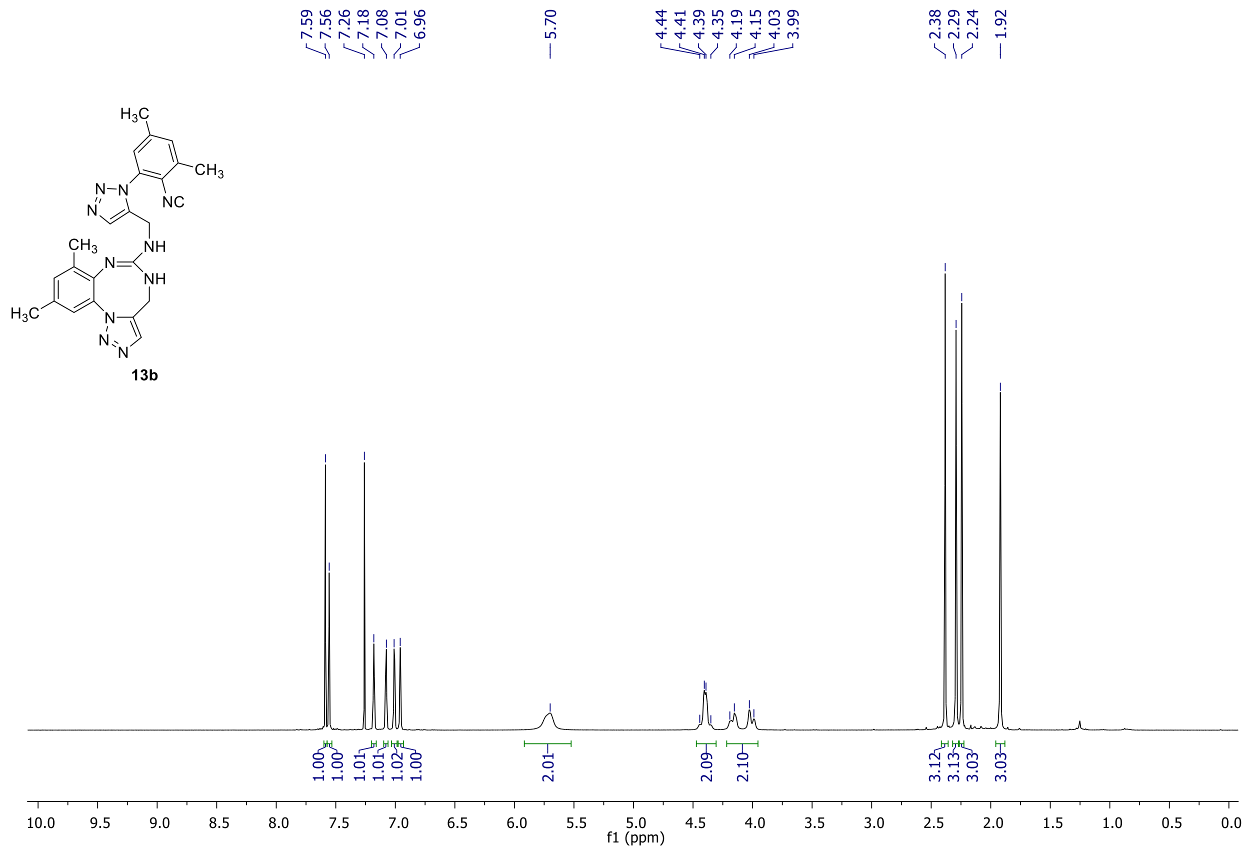

13b (^{13}C NMR, 150 MHz, $CDCl_3$, 298 K)

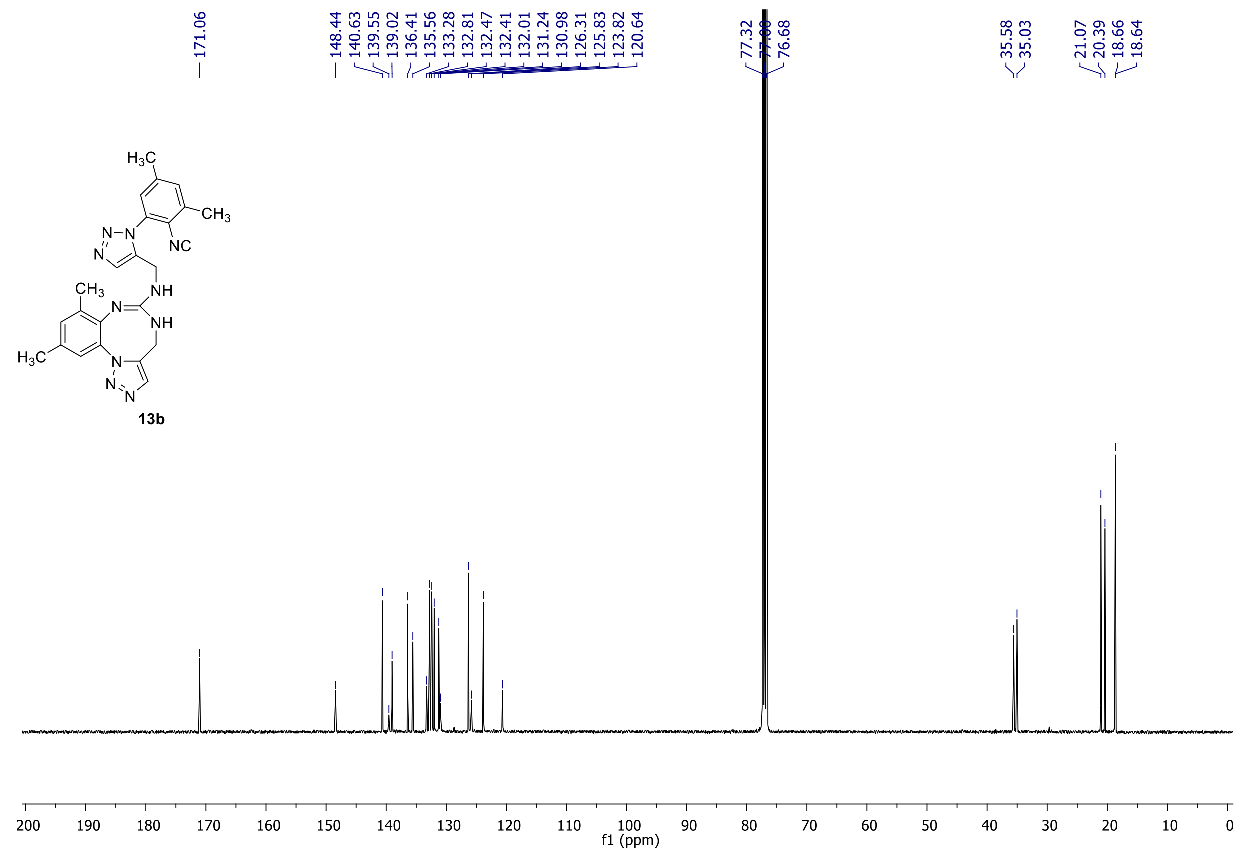

13b (DEPT-135 NMR, 150 MHz, $CDCl_3$, 298 K)

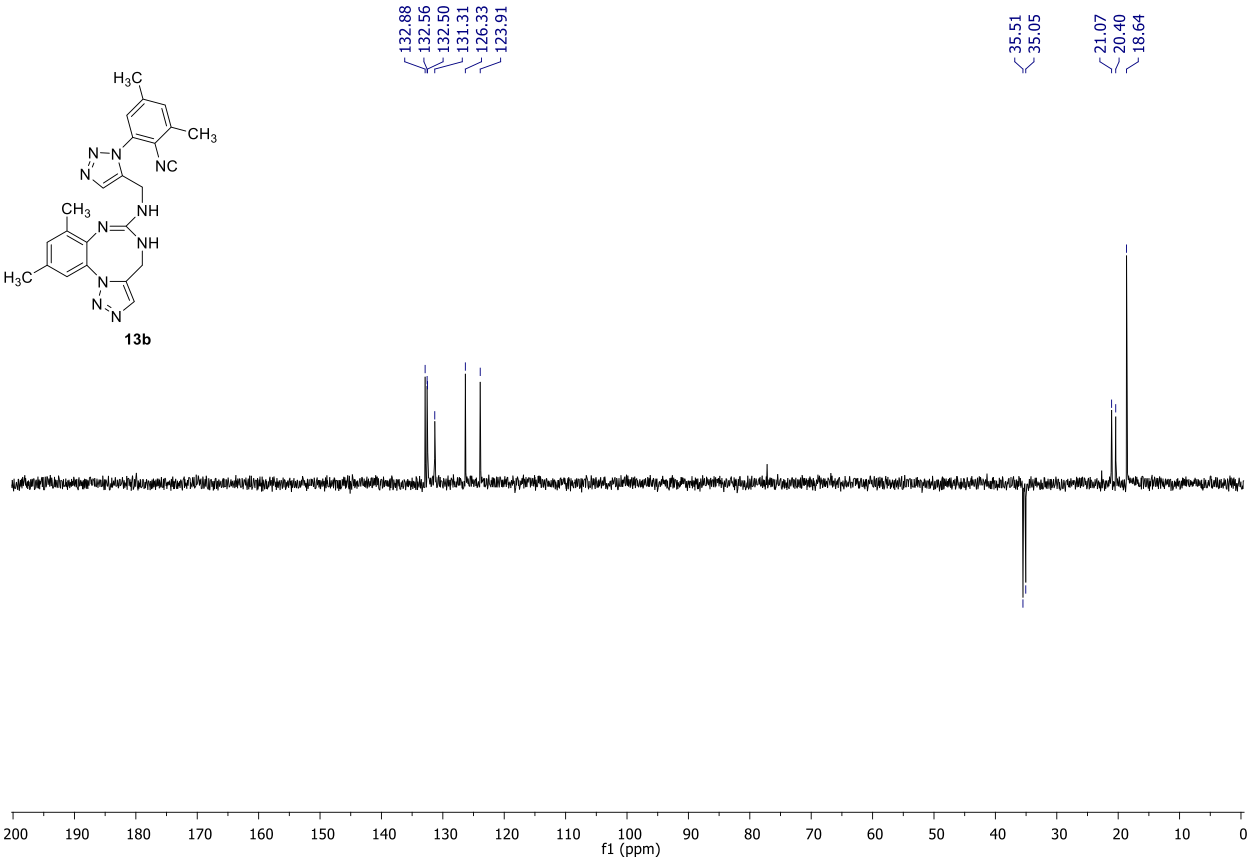

14b (^{1}H NMR, 300 MHz, CDCl$_3$, 298 K)

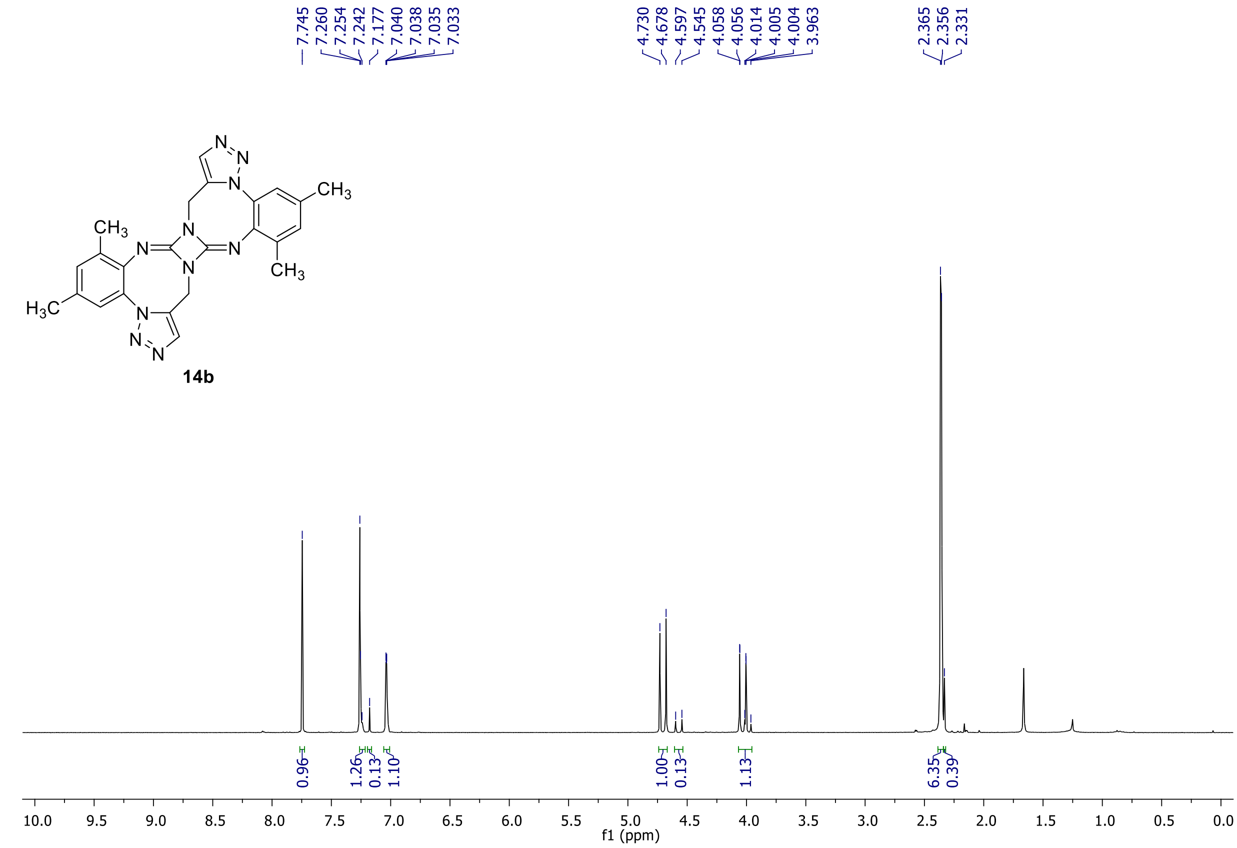

14b (^{13}C NMR, 75 MHz, CDCl$_3$, 298 K)

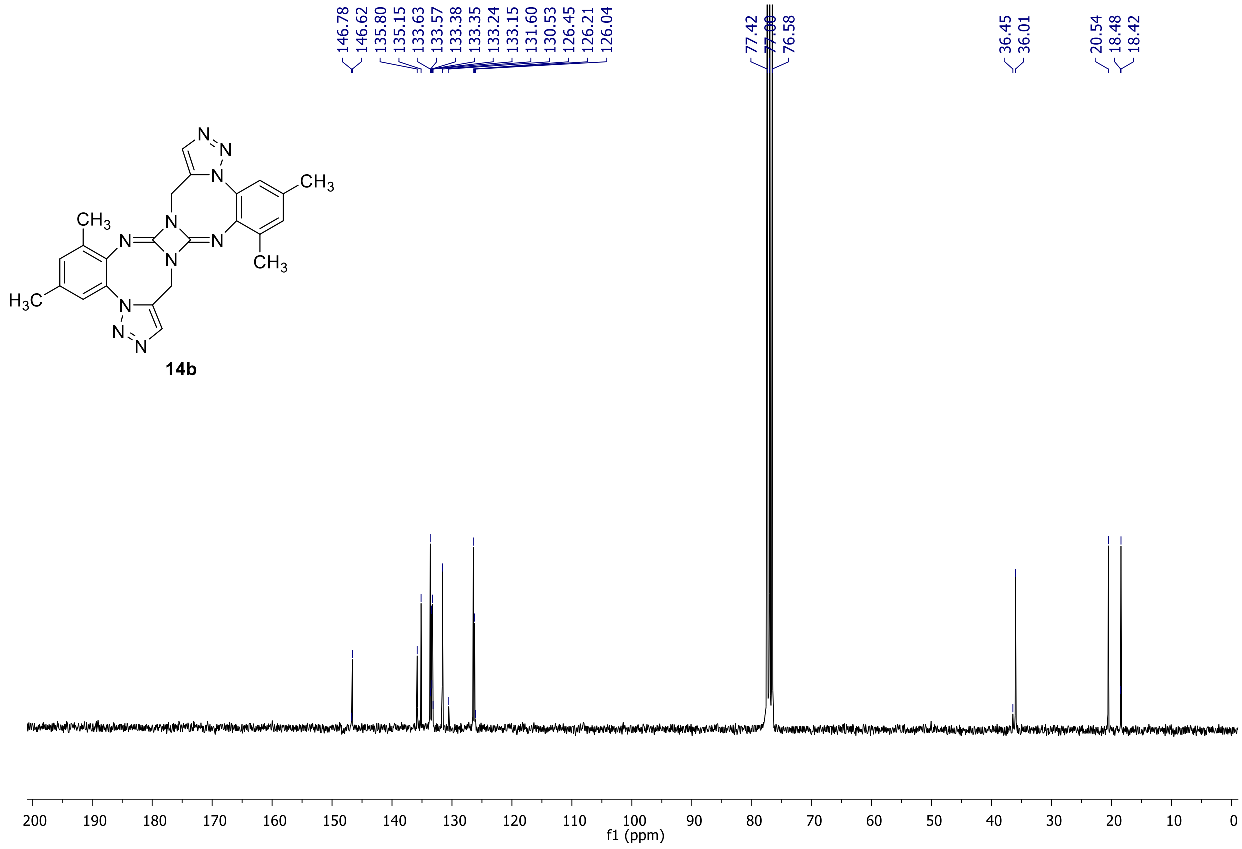

14b (DEPT-135 NMR, 75 MHz, $CDCl_3$, 298 K)

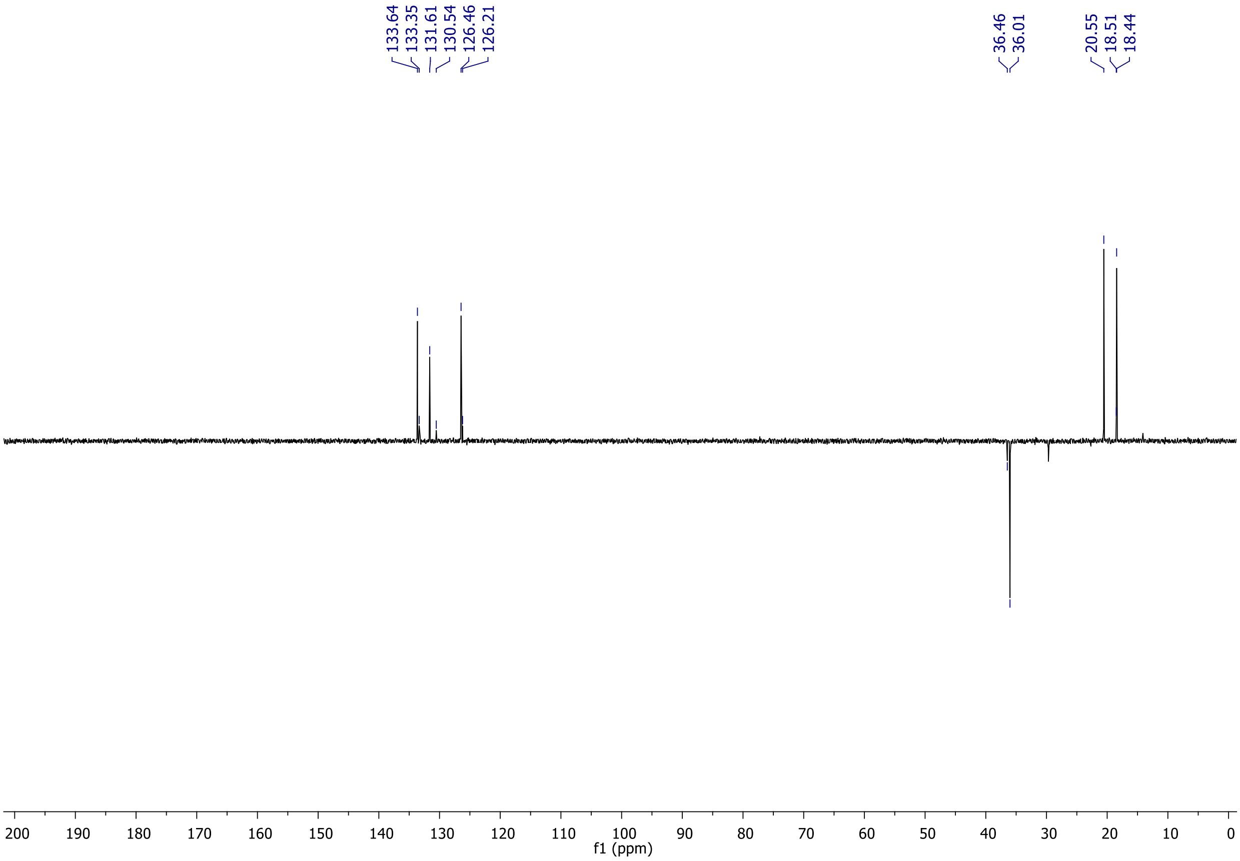

13. References

[1] *CRC Handbook of Phosphorus-31 Nuclear Magnetic Resonance Data*; Tebby, J. C., Ed.; CRC Press, Boca Raton (US), 1991.

[2] Alajarin, M.; Cutillas-Font, G.; Lopez-Leonardo, C.; Orenes, R.-A.; Marin-Luna, M.; Pastor, A. Intramolecular Cyclization of Azido-Isocyanides Triggered by the Azide Anion: An Experimental and Computational Study. *J. Org. Chem.* **2023**, *88*, 8658–8668.

[3.] Dolomanov, O. V.; Bourhis, L. J.; Gildea, R. J.; Howard, J. A. K.; Puschmann, H. OLEX2: a complete structure solution, refinement and analysis program. *J. Appl. Cryst*. **2009**, *42*, 339–341.

[4] Altomare, A.; Cascarano, G.; Giacovazzo, C.; Guagliardi, A. Completion and refinement of crystal structures with SIR92. *J. Appl. Crystallogr.***1993**, *26*, 343–350.

[5] (a) Sheldrick, G. M. F2 SHELXL-2014/7: Program for the Solution of Crystal Structures; University of Göttingen: Göttingen, Germany, **2014**; (b) Sheldrick, G. M. Crystal Structure Refinement with SHELXL. *Acta Cryst.* **2015**, C71, 3–8.

[6] Chai, J.-D.; Head-Gordon, M. Long-Range Corrected Hybrid Density Functionals with Damped Atom–Atom Dispersion Corrections, *Phys. Chem. Chem. Phys.* **2008**, *10*,6615–6620.

[7] Weigend, F. Accurate Coulomb-Fitting Basis Sets for H to Rn. *Phys. Chem. Chem. Phys.* **2006**, *8*, 1057–1065.

[8] Bauernschmitt, R.; Ahlrichs, R. Stability Analysis for Solutions of the Closed Shell Kohn–Sham Equation, *J. Chem. Phys.* **1996**, *104*, 9047–9052.

[9] Marenich. A. V.; Cramer, C. J.; Truhlar, D. G. Universal Solvation Model Based on Solute Electron Density and on a Continuum Model of the Solvent Defined by the Bulk Dielectric Constant and Atomic Surface Tensions, *J. Phys. Chem. B* **2009**, *113*, 6378–6396.

[10] Gaussian 16, Revision C.01. Frisch, M. J.; Trucks, G. W.; Schlegel, H. B.; Scuseria, G. E. et al. Gaussian. Inc., Wallingford CT. **2016**.
